# Bicyclopentadithiophene-Based Organic Semiconductor for Stable and High-Performance Perovskite Solar Cells Exceeding 22%

**DOI:** 10.1021/acsami.3c15774

**Published:** 2024-01-26

**Authors:** Arulmozhi Velusamy, Shakil N. Afraj, Yu-Sheng Guo, Jen-Shyang Ni, Hung-Lin Huang, Ting-Yu Su, Yamuna Ezhumalai, Cheng-Liang Liu, Chien-Hung Chiang, Ming-Chou Chen, Chun-Guey Wu

**Affiliations:** † Department of Chemistry, National Central University, Taoyuan 32001, Taiwan; ‡ Department of Chemical and Materials Engineering, National Kaohsiung University of Science and Technology, Kaohsiung 80778, Taiwan; § Department of Materials Science and Engineering, National Taiwan University, Taipei 10617, Taiwan

**Keywords:** perovskite solar cells, bicyclopentadithiophene, non-fullerene acceptors, grain boundary passivation, high performance

## Abstract

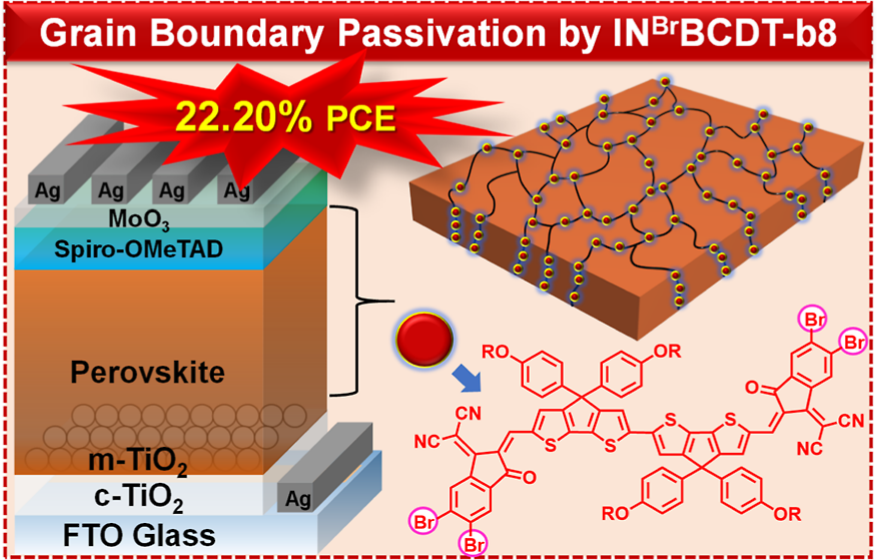

Well-performing organic–inorganic halide perovskites are susceptible to poor efficiency and instability due to their various defects at the interphases, grain boundaries (GBs), and surfaces. In this study, an in situ method is utilized for effectively passivating the under-coordinated Pb^2+^ defects of perovskite with new non-fullerene acceptors (NFAs) (**IN**^**X**^**BCDT**; X = H, Cl, and Br) through their carbonyl and cyano functional groups during the antisolvent dripping process. It reveals that the bicyclopentadithiophene (BCDT) core with highly electron-withdrawing end-capping groups passivates GBs and boosts perovskite grain growth. This effective defect passivation decreases the trap density to increase the carrier recombination lifetime of the perovskite film. As a result, bromo-substituted dicyanomethylene indanone (IN^Br^)-end-capped BCDT (**IN**^**Br**^**BCDT-b8**; **3a**)-passivated devices exhibit the highest power conversion efficiency (PCE) of 22.20% (vs those of 18.09% obtained for perovskite films without passivation) upon an optimized film preparation process. Note that devices treated with more soluble 2-ethylhexyl-substituted compounds (**1a**, **2a**, and **3a**) exhibit higher PCE than those treated with less soluble octyl-substituted compounds (**1b**, **2b**, and **3b**). It is also worth noting that BCDT is a cost-effective six-ring core that is easier to synthesize with a higher yield and therefore much cheaper than those with highly fused-ring cores. In addition, a long-term stability test in a glovebox for 1500 h reveals that the perovskite solar cells (PSCs) based on a perovskite absorber treated with compound **3a** maintain ∼90% of their initial PCE. This is the first example of the simplest high-conjugation additive for perovskite film to achieve a PCE greater than 22% of the corresponding lead-based PSCs.

## 1 Introduction

Due to the growing demand for sustainable energy, researchers mainly focus on the development of photovoltaic (PV) technologies, which convert solar energy into electrical energy. Compared to other futuristic PV technologies, perovskite solar cells (PSCs) are a potential candidate for renewable clean energy technology.^1−3^ In the last 10 years, PSCs have shown impressive advancements in conversion efficiencies from 3.8% in 2009^4^ to 26.1%^5^ today in a single-junction architecture. PSCs have therefore been regarded as the fastest-advancing solar technology in 2016.^6^ PSCs have also received widespread attention, primarily because of their exceptional optoelectronic characteristics,^7−9^ high efficiency, low weight, high flexibility, scalable low-temperature solution processability, color tunability, and simple fabrication processes, as well as low manufacturing cost.^1,10−15^ Despite their rapid advancement, the commercialization of PSC continues to confront obstacles, with inadequate stability being a prominent concern.^16^ In general, perovskite films fabricated at low-temperature result in inferior device efficiencies, mainly due to the defects at grain boundaries (GBs) and imperfect interfaces or surfaces.^17−22^ Besides, other instability problems are also caused by these defects, such as ion migration, current hysteresis, and device degradation.^7,23^ Passivation is a key strategy to obtain excellent surfaces and interfacial/GB properties while simultaneously promoting the efficiency and stability of the cells.^24^ Therefore, high-efficient and stable PSCs can be achieved through the efficient GB defect passivation of the perovskite films.^11,25^

**Figure 1:**
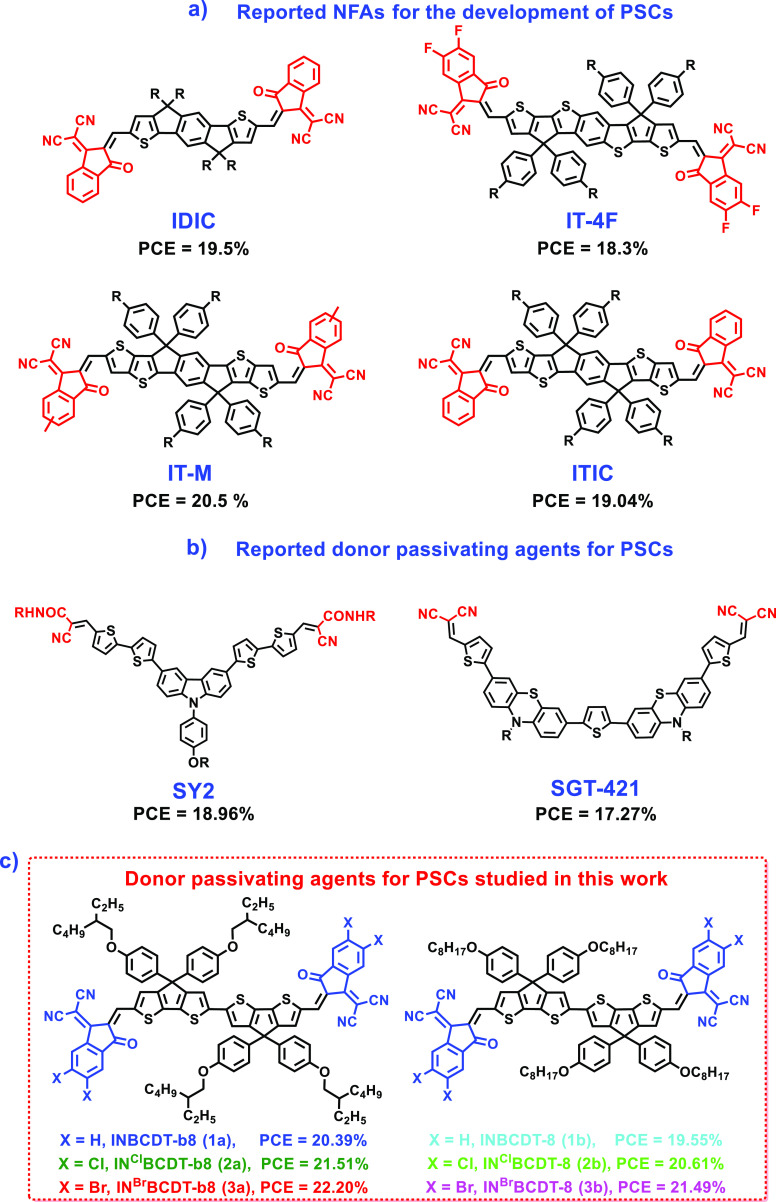
(a,b) Reported small molecules for the development of PSCs; (c) molecular structures and their performances with regard to BCDT-based compounds (**1**–**3**) of passivated lead-perovskite studied in this paper.

Post passivation treatment is successfully applied to decrease the number of defects at the perovskite framework by coordinating with under-coordinated halide or metal ions.^26−29^ The smart passivation approach involves employing various materials such as metal cations,^30,31^ organic cations,^32,33^ anions,^34,35^ zwitterions,^36,37^ Lewis acids,^38,39^ and Lewis bases,^26,40^ which possess electron-donating/accepting capabilities.^24^ Among these materials, Lewis-base passivators are well-known passivators to reduce defect densities as well as enhance the device performance of the PSCs, which requires further investigation.^29^ Lewis bases, such as compounds containing sulfur/nitrogen/oxygen, graphene oxide, n-type π-conjugated molecules, and polymers, have been used as passivating agents.^24^ However, only a few reports have been found to study GB passivation using electron acceptors/n-type π-conjugated molecules as additives/passivators in PSCs.^41−45^ Non-fullerene acceptors (NFAs) are some of the π-conjugated Lewis base n-type molecules possessing a highly electron-donating planar molecular framework with suitable side chains and strong electron-withdrawing end-capped units. For example, recently reported **IDIC**,^27^**IT-4F**,^7^**IT-M**,^46^ and **ITIC**(45) NFAs are excellent performing n-type π-conjugated materials in organic PVs. They have also been demonstrated for effective passivation in PSCs to achieve power conversion efficiency (PCE) of 19.5, 18.3, 20.5, and 19.04%, respectively (Figure 1a). In addition, our group developed a chlorinated seven thiophene ring-fused (DCDTT)-based NFA, **IN**^**Cl**^**-DCDTT**, as a passivation agent, and enhanced PCE up to 21.39% was recently achieved.^47^ These compounds contain oxygen, nitrogen, and sulfur atoms of the carbonyl, cyano, and thiophene groups were proposed to coordinate with Pb^2+^ ions, passivating the defects through their nonbonded electrons.^24^ In addition, cyano/carbonyl units containing organic small molecules, **SY2**(48) and **SGT-421**,^49^ were developed and reported as donor-passivating agents in perovskite film and exhibited enhanced PCE of 18.96 and 17.27%, respectively (Figure 1b), for the corresponding PSCs. The results suggest that NFAs are potential efficient passivators for Pb-based perovskite. However, most of these highly efficient passivating NFAs feature a large fused-ring central core (5–7 fused-rings), which usually requires a tedious multiple-step synthesis. According to our own synthetic experience, the yields of these six- to seven-ring-fused NFAs are quite low, which will preclude their practical/commercial applications. Consequently, investigating NFAs with simple synthesis and easy modification in their optoelectronic properties is essential. This exploration is crucial for the production of affordable, high-efficient, and scalable Pb-based PSCs.^13^ In the current work, as shown in Figure 1c, six newly easy-accessible bicyclopentadithiophene (**BCDT**)-based NFAs, **INBCDT-b8** (**1a**), **INBCDT-8** (**1b**), **IN**^**Cl**^**BCDT-b8** (**2a**), **IN**^**Cl**^**BCDT-8** (**2b**), **IN**^**Br**^**BCDT-b8** (**3a**), and **IN**^**Br**^**BCDT-8** (**3b**), are developed to serve as passivating agents for Pb-based perovskites applied in solar cells.

Figure S1 illustrates the molecular design and approach to efficient NFA passivators for perovskites based on the theoretical calculation and experimental results. This takes advantage of the simple synthesis of these six A–D–A-type NFAs, where acceptor (A) and donor (D) groups have been prepared individually and then coupled via Knoevenagel condensation. Even though fluorination on the acceptor group is a fruitful approach,^50−52^ the process of fluorination is generally complicated and expensive, which is adverse for commercial applications.^53^ Therefore, incorporation of cost-effective chlorinated and brominated indanones, derived from inexpensive starting materials, into the NFA enhances the intramolecular charge transfer (ICT) effect and thus helps to adjust their energy levels as well as perform comparative studies on the resulting cells. Moreover, strong electron-donating alkoxy phenyls were obtained from easier and more inexpensive synthetic procedures to replace the regular alkyl phenyl substitutions, which require catalyzed synthesis. Another important factor is solubility, which is a crucial issue for using NFAs as an additive. For better solubility, we incorporated branched alkyl chains into the structure. Among the six BCDT-based compounds, branched alkyl (2-ethylhexyl)-substituted compounds (**1a**, **2a**, and **3a**) possess higher solubility than the other three linear alkyl (octyl)-substituted compounds (**1b**, **2b**, and **3b**). Interestingly, the PCEs of PSCs based on perovskite passivated by **1a**, **2a**, and **3a** are also higher than those of the cells based on perovskite passivated by their respective linear alkyl-substituted ones (**1b**, **2b**, and **3b**). The brominated-BCDT compound **3a** exhibits suitable energy levels, and the perovskite films with the addition of **3a** demonstrate larger grain sizes and enhanced crystallinity compared to the original pristine perovskite (CB) films. Thus, the highest PCE of **22.20%** was achieved for the PSCs based on **3a** passivated perovskite. Note that a dimer of cyclopentadithiophene (CDT), BCDT, is a cost-effective six-ring core that is easier to synthesize with a higher yield compared to those highly fused-ring cores. As far as our knowledge extends, this marks the prime instance of utilizing the simplest high conjugation system of a cost-effective brominated small molecular NFA to be utilized as a donor passivation agent during an antisolvent dripping process for Pb-based perovskites. This has led to the attainment of a PCE greater than 22% in the corresponding solar cells. It is noted that PCEs of all the newly synthesized compounds, **1** (**a–b**), **2** (**a–b**), and **3** (**a–b**)-passivated devices, are significantly exceeding the efficiency of the control device (18.09%). Overall, the results suggest that exceptionally simple NFAs are involved in efficient defect passivation and reveal their important aspects in the molecular design with great future application.

## 2 Results and Discussion

**Scheme 1:**
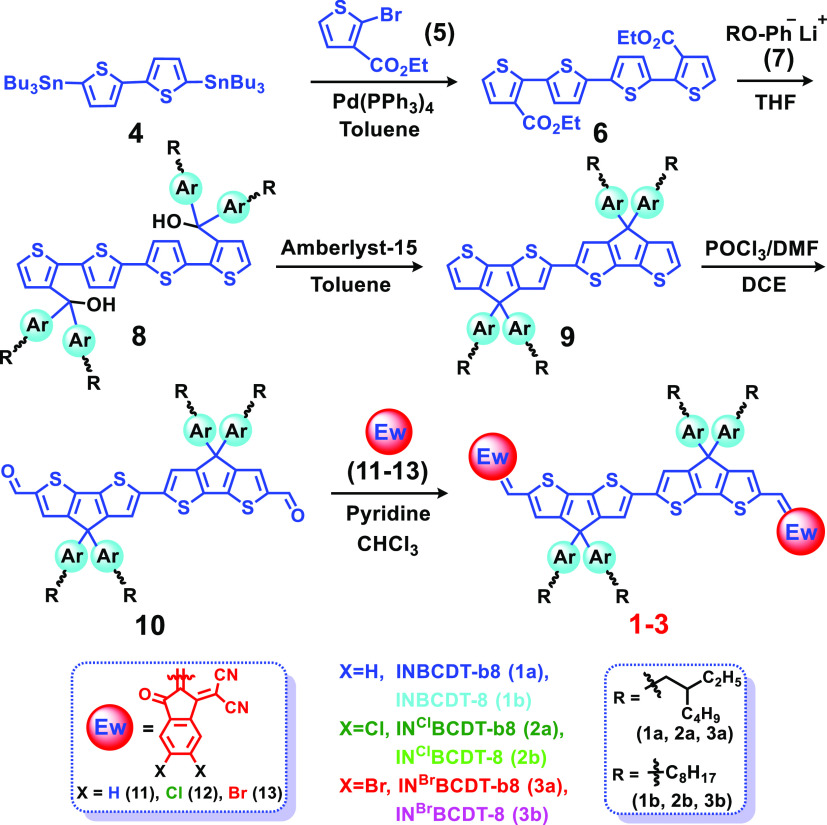
Synthetic Route to **IN**^**X**^**BCDT** Series (Compounds **1–3**)

**IN**^**X**^**BCDT** compounds were synthesized as represented in Scheme 1, and the detailed procedures are given in Schemes S1–S4. In brief, distannylated bithiophene **4** was first coupled with ethyl 2-bromothiophene-3-carboxylate (**5**) by using Pd(PPh_3_)_4_ to give diesters of tetrathiophene **6**. Then, via an alkoxyphenyl anion (**7**) addition to the diesters of **6**, benzylic alcohol **8** was produced. The latter was catalyzed by Amberlyst-15, and a BCDT core (**BCDT**; **9**) was obtained. Further, treating **9** with POCl_3_/DMF via the Vilsmeier–Haack reaction yields dialdehyde **10**. Finally, the Knoevenagel condensation of dialdehyde **10** with indanones (**11**–**13**) gives target compounds (**1**–**3**). The spectra of all characterized compounds are shown in Figures S12–S52. Note that the solubility factor of these compounds is explored by incorporating branched/linear octyl chain substituents onto the central **BCDT** core. Comparatively, all three branched C_8_H_17_-substituted compounds (**1a**, **2a**, and **3a**) possess better solubility than their linear C_8_H_17_-substituted analogues (**1b**, **2b**, and **3b**).

**Figure 2:**
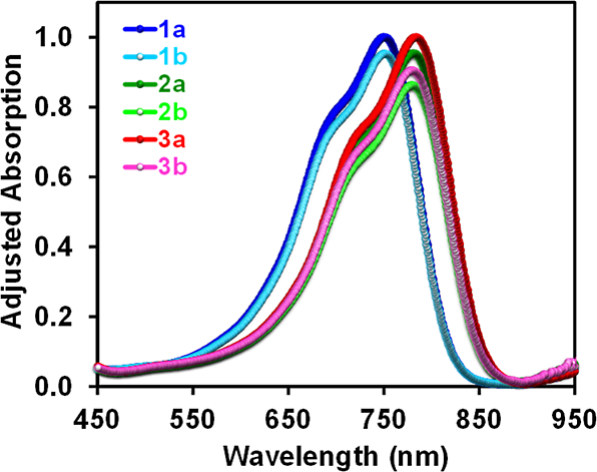
UV–vis absorption spectra of **IN**^**X**^**BCDT**s.

**Figure 3:**
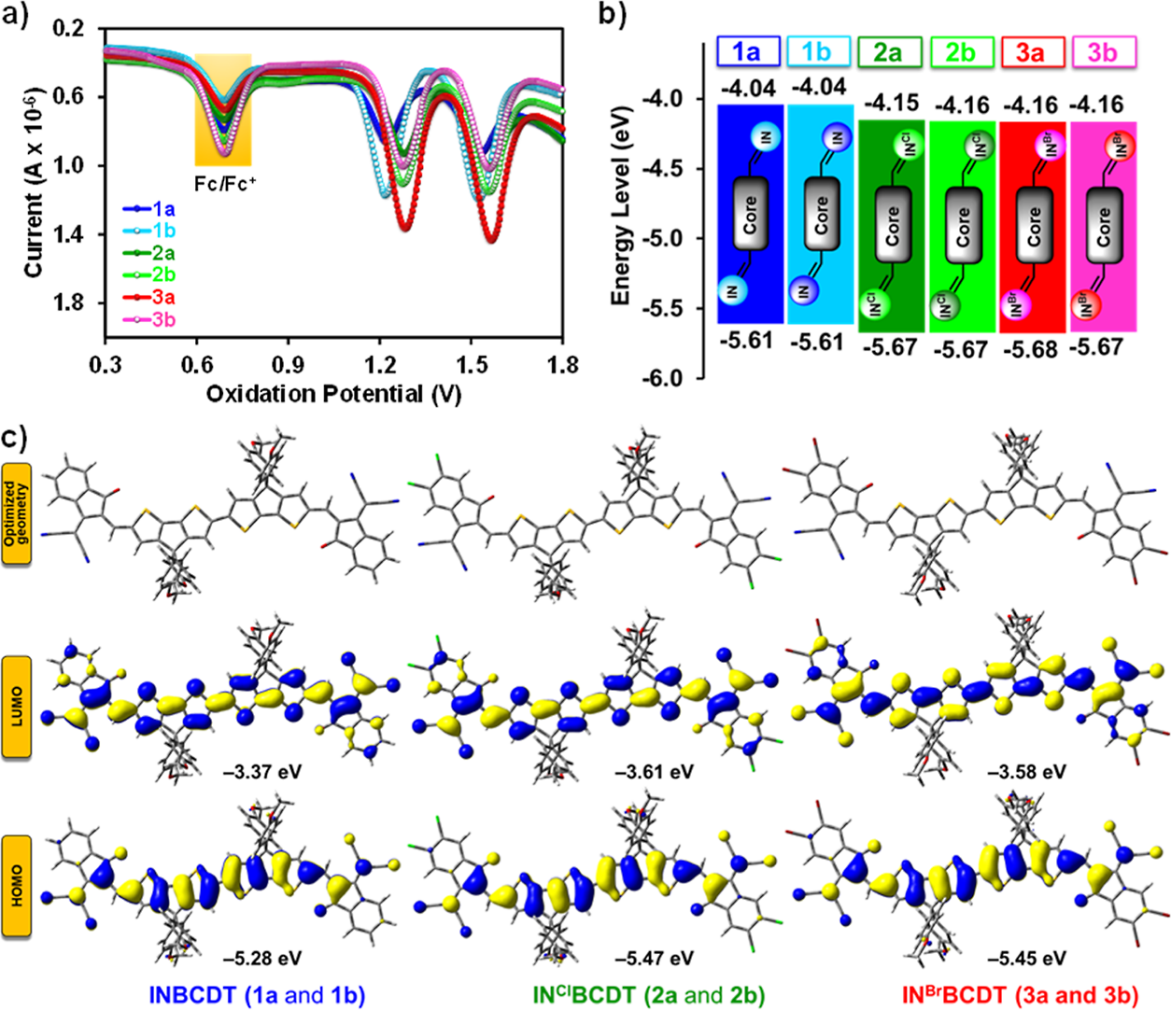
(a) DPV curves; (b) HOMO and LUMO values from DPV; and (c) DFT of **IN**^**X**^**BCDT** compounds (alkyl chains are replaced with –CH_3_ groups for simplicity).

The physicochemical properties of the **IN**^**X**^**BCDT** derivatives (**1**–**3**) are outlined in Table 1. Thermogravimetric analysis (TGA; Figure S2) showed that all six **IN**^**X**^**BCDT** derivatives are thermally stable and exhibit *T*_d_ in the range of 340–369 °C. Subsequently, the melting point for all BCDTs occurred at >320 °C, proving the exceptional thermal stability of these compounds, suitable for device fabrication and later operation. Figure 2 depicts the UV–vis of the **IN**^**X**^**BCDT** compounds in *o*-dichlorobenzene (*o*-DCB). As expected, there is no large variation in the maximum absorption (λ_max_) by changing the alkyl chain (branched octyl vs linear octyl) of NFAs in the solution state.^54,55^ A distinct bathochromic shift (∼30 nm) has been observed for the maximum absorption peaks of brominated (**3a** and **3b**; 784 and 779 nm) and chlorinated (**2a** and **2b**; ∼780 nm) NFAs in contrast to the nonhalogenated derivatives (**1a** and **1b**; 750 nm). The energy gaps [Δ*E*_g_ (opt) values] obtained for compounds **2** and **3** (∼1.43 eV) are smaller than those of **1** (1.49 eV). The trend is consistent with the values obtained from the differential pulse voltammetry (DPV) measurement (Figure 3a), in which the energy gaps of **2** and **3** (∼1.50 eV) are confirmed to be smaller than those of **1** (1.56 eV; Table 1 and Figure 3b; vide infra).^15,53,54,56,57^

**Table 1:** Photophysical and Electrochemical Data of **IN**^**X**^**BCDT**s

passivating agents	*T*_d_^a^ [°C]	*T*_m_^b^ [°C]	λ_abs_ (soln) [nm]^c^	Δ*E*_g_ (opt) [eV]^d^	*E*_ox_ [V]^e^	HOMO [eV]^f^	*E*_red_ [V]^e^	LUMO [eV]^f^	Δ*E*_g_ (DPV) [eV]^g^	Δ*E*_g_ (DFT) [eV]^h^
**INBCDT-b8** (**1a**)	369	359	750	1.49	1.17	–5.61	–0.40	–4.04	1.57	1.91
**INBCDT-8** (**1b**)	369	358	750	1.49	1.17	–5.61	–0.40	–4.04	1.56	
**IN**^**Cl**^**BCDT-b8** (**2a**)	340	335	780	1.44	1.23	–5.67	–0.29	–4.15	1.52	1.86
**IN**^**Cl**^**BCDT-8** (**2b**)	355	328	779	1.44	1.23	–5.67	–0.28	–4.16	1.51	
**IN**^**Br**^**BCDT-b8** (**3a**)	342	321	784	1.43	1.24	–5.68	–0.28	–4.16	1.51	1.87
**IN**^**Br**^**BCDT-8** (**3b**)	347	341	779	1.44	1.23	–5.67	–0.28	–4.16	1.50	

a By TGA.

b From melting point apparatus.

c Determined in *o*-DCB.

d Calculated by using the optical absorption onset, Δ*E*_g_ = 1240/λ_onset_.

e By DPV in *o*-DCB.

f HOMO/LUMO = −(4.44 + 0.64 + *E*_ox_/*E*_red_).

g Δ*E*_g_ = *E*_LUMO_ – *E*_HOMO_ from DPV.

h The energy gap was derived from DFT calculations (alkyl chains are replaced with –CH_3_ groups).

The electrochemical behavior of **IN**^**X**^**BCDT**s (**1**–**3**) was examined with Bu_4_N^+^PF_6_^–^ as an electrolyte (Figures 3a and S3).^55^ The electrochemically derived HOMO and LUMO energies (Figure 3b) and corresponding values (Table 1) are given. The LUMO and HOMO of compounds **1**–**3** are calculated according to the equation: HOMO/LUMO = −(4.44 + 0.64 + *E*_ox_*/E*_red_). The DPV data clearly confirms that the variation in the alkyl chain (branched octyl vs linear octyl) has no significant effect on the electrochemical properties of NFAs, and each pair possesses similar oxidation and reduction potentials.^54,58^ In contrast, halogenated compounds **2** and **3** feature more positive oxidation (*E*_ox_ = +1.23 V) and reduction (*E*_red_ = −0.28 V) potentials in comparison to their analogue **1** (*E*_ox_ = +1.17 V, *E*_red_ = −0.40 V), which leads to lower HOMO (−5.67 eV) and LUMO (−4.16 eV) vs seen with nonhalogenated **1** [HOMO (−5.61 eV) and LUMO (−4.04 eV)]. Due to the electron-deficient nature of the halogenated indanones, compounds **2** and **3** display a shift toward higher values in both oxidation and reduction potentials in comparison to the potentials noticed for **1**.^56,59,60^ In particular, the LUMO levels of halogenated NFAs (**2** and **3**) are much lower than those of nonhalogenated ones (**1**), comparing the difference in HOMO levels, resulting in the energy gap contractions of NFAs **2** and **3** compared to **1**. Consequently, the narrower energy gaps were confirmed for the halogenated **2**–**3** (∼1.50 eV) vs **1** (1.56 eV), which is in line with their optical energy gap values: **2**–**3** (∼1.44 eV) < **1** (1.49 eV). Moreover, this trend is also consistent with the DFT-derived energy gap values as **2**–**3** (∼1.87 eV) < **1** (1.91 eV; vide infra).

To investigate the impact of the chemical geometry structure of the six **IN**^**X**^**BCDT** molecules on passivation ability, DFT calculations were employed at the B3LYP/6-31G* level of the Gaussian 03W program. The computational results indicate that the HOMO is primarily distributed across the central BCDT, whereas the LUMO is situated on the end-group IN-derivatives or the whole backbone.^61^ Furthermore, it was found that IN^Cl^ and IN^Br^-substituted compounds possess near planar conformation with the smallest dihedral angles of 0.24 and 2.66°, respectively, between the two CDT units (whereas 8.74° for nonhalogenated derivatives). It was postulated that coplanarity in BCDTs was facilitated by noncovalent contacts (S···O), therefore extending the effective π-conjugation length (Figure S1). Indeed, these proposed interactions (S···O) were supported by DFT calculations, which showed that the dihedral angle between core and end-capping units was from −0.37 to 1.03°, which could conformationally lock the coplanar geometry for enhanced charge transport.^62−64^ The calculated HOMO/LUMO energy levels of the chloro- and bromo-substituted compounds **2** (−5.47 eV/–3.61 eV) and **3** (−5.45 eV/–3.58 eV) also exhibit lower HOMO and LUMO energy levels compared to **1** (−5.28 eV/–3.37 eV; Figure 3c). From Table 1, the optically, electrochemically, and theoretically derived energy gaps trends of these **IN**^**X**^**BCDT**s are consistent with each other in the order of **2**–**3** < **1**, as a result of the electron-deficient nature of the halogenated indanones.

**Figure 4:**
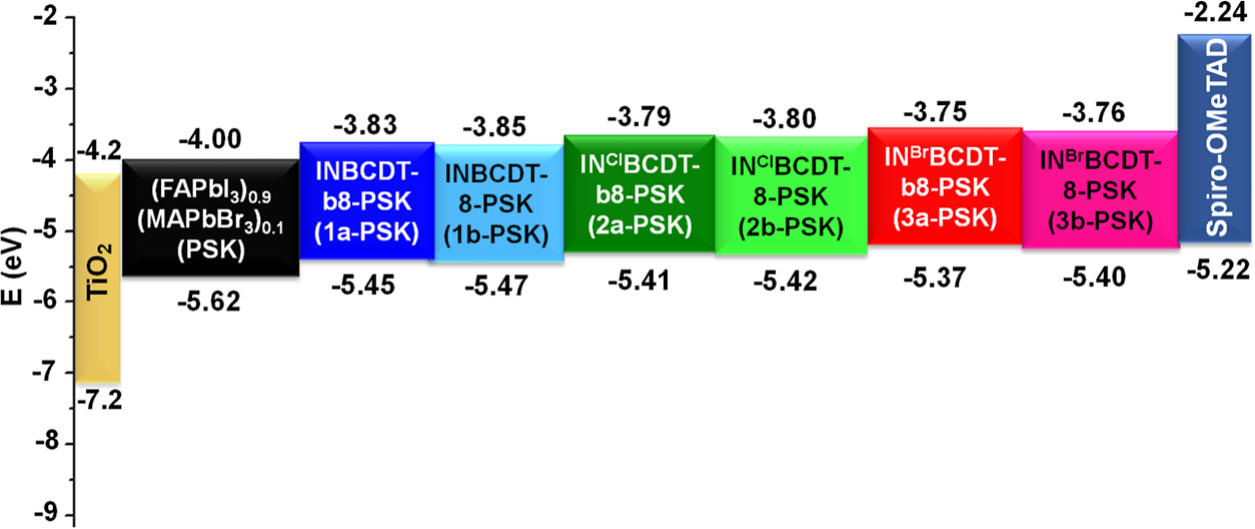
Schematic representation of the energy levels.

The physicochemical properties, morphology, photophysics, efficiency, and stability of the respective PSCs were investigated both with and without passivation. The **IN**^**X**^**BCDT-**treated perovskite films were prepared by dissolving **IN**^**X**^**BCDT** in the chlorobenzene (CB) as an antisolvent during the spin coating process (see Supporting Information). The pristine perovskite (perovskite film prepared from the pure antisolvent CB) was used as a reference for the comparison. The valence band (VB) energy levels of perovskites were assessed using ultraviolet photoelectron spectra (UPS), as depicted in Figure S4, instead of relying on the electrochemical method to determine the VB of the **IN**^**X**^**BCDT** passivators. The band gap of perovskite films was determined through a Tauc plot, as illustrated in Figure S5. The energy level diagrams for TiO_2_, pristine perovskite, **IN**^**X**^**BCDT-**passivated perovskites, and HTL (Spiro-OMeTAD) are presented in Figure 4, with corresponding numerical data provided in Table S1 of the Supporting Information for the clarity. The HOMO energy level of the perovskite film treated with **IN**^**Br**^**BCDT-b8** (named **3a**-PSK) is measured at −5.37 eV. This value closely aligns with the HOMO of the Spiro-OMeTAD, which is −5.22 eV, resulting in minimal voltage loss. The LUMO energy level of **3a**-PSK is recorded at −3.75 eV, making it conducive for the efficient transport of electrons. Moreover, the UV–vis absorption spectra of both pristine perovskite and **IN**^**X**^**BCDT**-treated perovskite films reveal that the passivated perovskite film exhibits stronger absorption in the 500–750 nm range (refer to Figure S6). This increased absorption implies an enhanced capacity to capture more light within this wavelength range, potentially attributed to the close packing, larger grain size, or greater crystallinity of the passivated perovskite film.^13^ It is worth noting that perovskite films passivated with **IN**^**X**^**BCDT** featuring branched alkyl chains (**1a**-PSK, **2a**-PSK, and **3a**-PSK) have stronger absorption in the range of 400–500 nm (see Figure S6), suggesting a reduced hydration level in the perovskite. However, the energy gaps derived from the optical Tauc plots, as depicted in Figure S5, are comparable. This similarity indicates that the observed differences in film quality are not substantial enough to significantly impact the bandgap energy of the perovskite.

**Figure 5:**
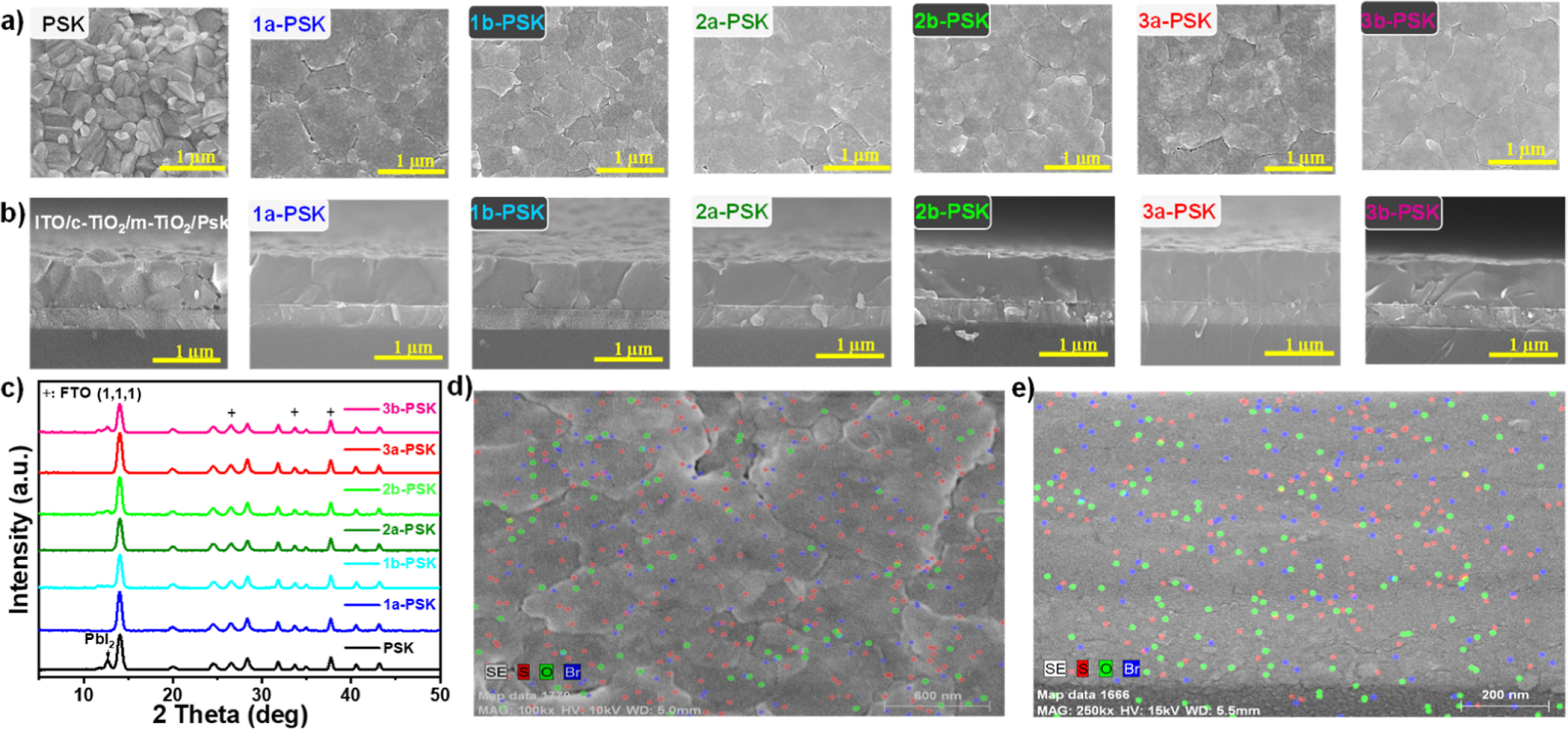
(a) Surface topography and (b) cross-section SEM images; (c) XRD profiles; (d) top view; and (e) cross-section view of SEM-EDS elements mapping for **3a**-PSK (sulfur, oxygen, and bromine atoms are in red, green, and blue colors, respectively).

The scanning electron microscopes (SEMs) depicted in Figure 5a highlight the morphological differences between the pristine and passivated **IN**^**X**^**BCDT** perovskite films. The pristine perovskite film exhibits an irregular texture characterized by roughness and an uneven grain size, resulting in distinctly visible GBs. Surprisingly, the perovskite films prepared using **IN**^**X**^**BCDT** CB solution as the antisolvent are flat films with large grain size. SEM topographies demonstrate that antisolvent containing **IN**^**X**^**BCDT** (**1a**–**1b**, **2a**–**2b**, and **3a**–**3b**) has the potential to influence the crystallization process of perovskite, leading to the formation of perovskite films of superior quality. In Figure 5b, cross-sectional SEM images reveal that the pristine perovskite film exhibits pinhole defects in the vertical direction. Additionally, the perovskite grain appears to be loosely attached to the TiO_2_ layer. In contrast, perovskite films passivated with **IN**^**X**^**BCDT**, especially **3a**-PSK, exhibit no obvious pinhole and attach closely to the TiO_2_ underlayer. The presence of pinhole defects at the interface adversely affects charge transport and collection. That is why PSCs based on **IN**^**X**^**BCDT**-treated perovskite demonstrate superior PV performance compared to those utilizing a pristine perovskite absorber. X-ray diffraction (XRD) patterns showcased in Figure 5c reveal the crystallinity and phase characteristics of both pristine perovskite and **IN**^**X**^**BCDT**-passivated perovskite films (**1a**–**1b**_(CB)_, **2a**–**2b**_(CB)_, and **3a**–**3b**_(CB)_). Notably, all perovskite films exhibit identical characteristic peaks in their XRD patterns. However, a PbI_2_ diffraction peak was observed in the pristine perovskite film. This occurrence is attributed to the film preparation process, which involves exposure to air for 2 h. Consequently, some perovskite in the pristine film decomposed to PbI_2._ Moreover, among all of the perovskite films investigated in this study, the (100) diffraction peak intensity of the **3a**-PSK film stands out as the strongest. These results proved that treatment with **IN**^**Br**^**BCDT-b8** (**3a**) distinctly enhances both the quality and stability of the perovskite film. To illustrate the distribution of compound **3a** in perovskite film, SEM X-ray energy dispersive spectroscopic (EDS) element mapping (Figure 5d,e) was conducted on both the top and cross-section of **3a**-PSK film. The uniform distribution of **IN**^**Br**^**BCDT-b8** across the entire film indicates that this compound effectively passivates the entire perovskite film. Figure 5d,e also reveals the thorough penetration of **IN**^**Br**^**BCDT-b8** (**3a**) throughout the entire perovskite layer, displaying a randomized vertical distribution. Moreover, the branched alkyl chains of compound **3a** that existed in the GBs can prevent water vapor from damaging the perovskite via the frail GB.

**Figure 6:**
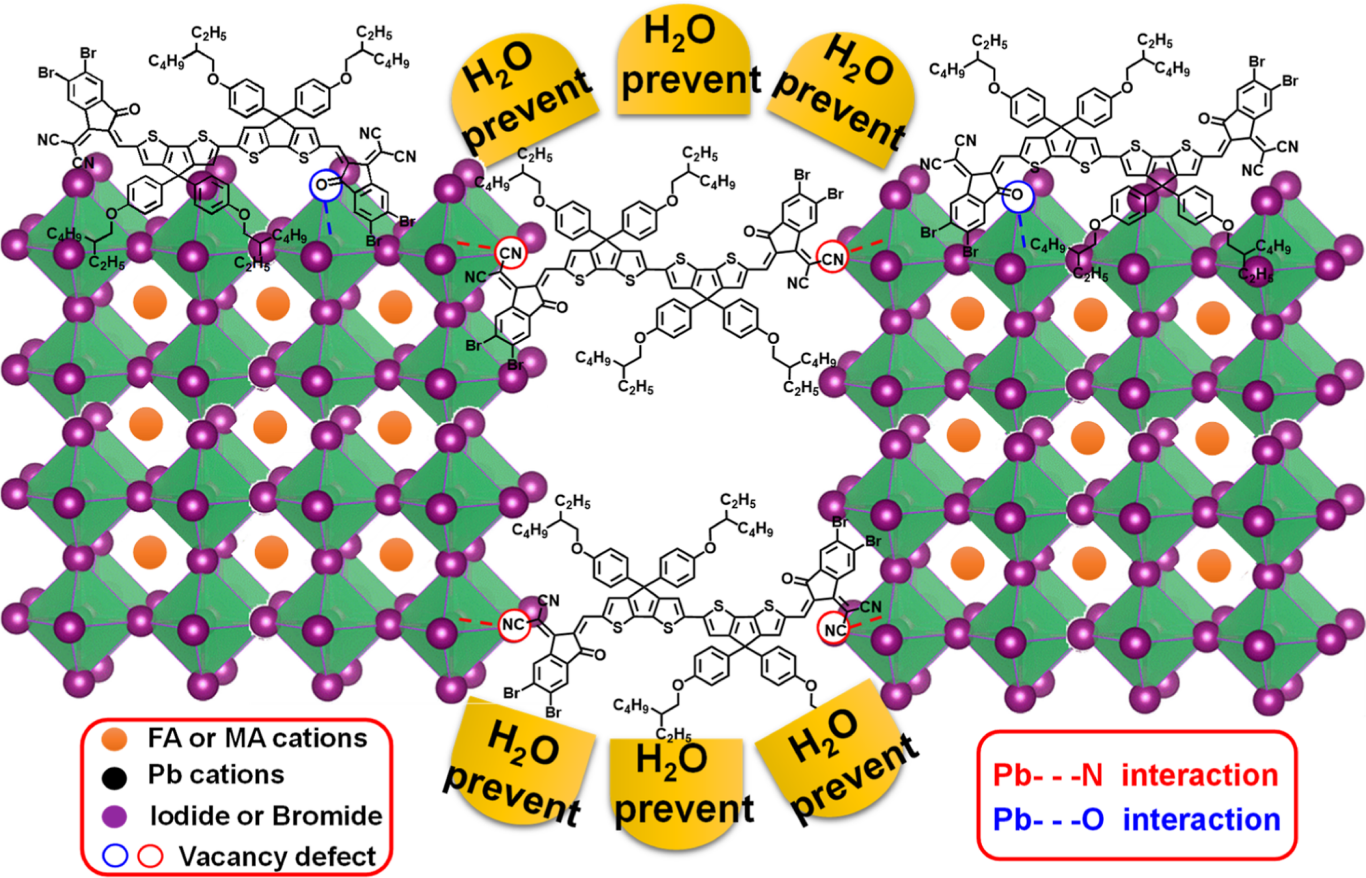
Schematic diagram of GB passivation by compound **3a**, which can prevent H_2_O from entering to degrade the perovskite.

Fourier-transform infrared (FTIR) analysis was performed for compound **3a** and the mixture of **3a** and PbI_2_, to further investigate the interactions between **IN**^**Br**^**BCDT-b8** (**3a**) and under-coordinated Pb^2+^ ions on the grain surface/interface (Figure S7). As a result, lower wavenumbers are observed for the mixture of **3a** and PbI_2_ [C=O bond (1687 cm^–1^) and C≡N bond (2210 cm^–1^)], compared to those of compound **3a** [C=O bond (1715 cm^–1^) and C≡N bond (2238 cm^–1^)]. This shift indicates that **3a** possesses the ability to passivate Pb^2+^ defects in perovskite, a finding consistent with observations in various reports.^42,65^Figure 6 illustrates the schematic representation of the interaction between the carbonyl and cyano moieties of **3a** and Pb^2+^, along with the hydrophobic nature of the branched alkyl chain of **3a** within the perovskite layer. In the **3a**-PSK system, compound **3a** acts as a passivator, contributing to the development of high-density perovskite films with large grains. This functionality is expected to lead to better performance and stability of devices based on **IN**^**X**^**BCDT**-treated perovskite absorbers.

**Figure 7:**
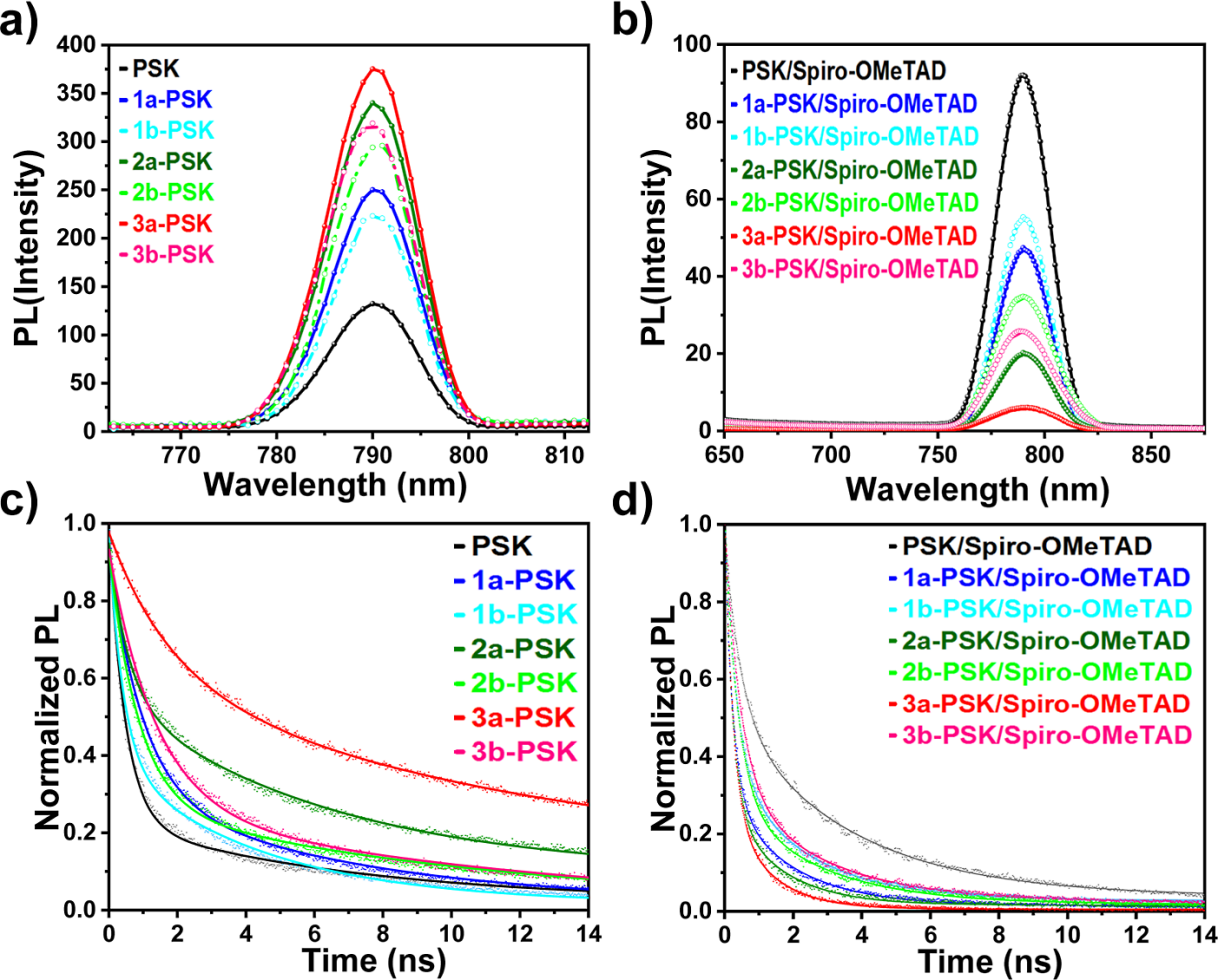
Steady-state PL (a,b) and TRPL (c,d) spectra of perovskite films (on glass/with Spiro-OMeTAD overlayer).

Steady-state (PL) and time-resolved (TRPL) were conducted for both pristine and **IN**^**X**^**BCDT**-passivated perovskite films to investigate the impact of passivation on the hole transporting efficiency. These measurements were taken on glass substrates as well as on films coated with HTL, and the outcomes are presented in Figure 7a–d. The PL intensity and estimated exciton lifetimes for all of the perovskite films are listed in Tables S2–S4, respectively. With the application of HTL, the perovskite film incorporated with compound **3a** exhibits significantly lower PL intensity compared to other **IN**^**X**^**BCDT**-treated perovskite films (Figure 7b). This reduction in PL intensity is attributed to the enhanced charge transfer efficiency from the perovskite to the HTL. As depicted in Figure 7a, in the absence of the HTL top layer, the PL intensity of the **3a**-PSK is the strongest, suggesting that the **IN**^**Br**^**BCDT-b8-**treated perovskite film exhibits superior characteristics with fewer defect sites. In accordance with previous literature, the exciton lifetime can be extracted from the normalized curves of TRPL.^66^ All of the passivated perovskite films showed a significant increase in the PL decay time. Furthermore, **3a**-PSK demonstrated the longest PL decay time of 7.52 ns, indicating the best quality (the lowest defect density) of the **IN**^**Br**^**BCDT-b8-**treated perovskite film. Conversely, the PL decay time was dramatically reduced for all passivated perovskite films overlaid with HTL. Nevertheless, a transient decay time of 0.45 ns was observed for **3a**-PSK with HTL among all of the perovskite films studied in this paper, suggesting that **3a**-PSK exhibits the most effective hole transport ability. The TRPL data support the conclusion that **IN**^**Br**^**BCDT-b8** (**3a**) is the best passivator for perovskite film among the passivators studied in this paper.

**Figure 8:**
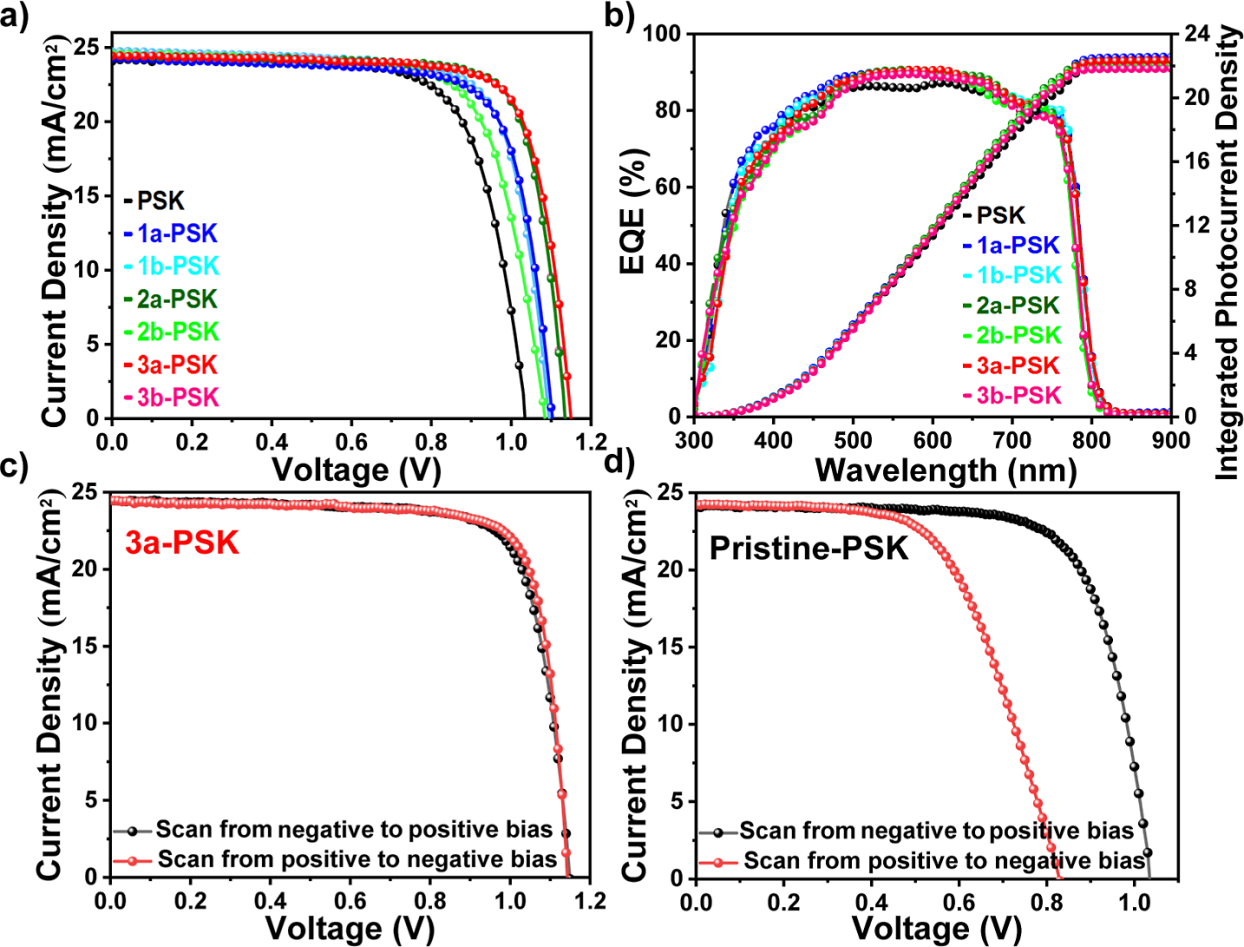
(a) *J*–*V* curves; (b) EQE spectra; (c) *J*–*V* curves of the devices with **3a**-PSK; and (d) with pristine PSK (in both forward and reverse scans).

PSCs with a regular (n–i–p) configuration were fabricated by using the architecture FTO/c-TiO_2_/m-TiO_2_/PSK/HTL/MoO_3_/Ag. Detailed device fabrication is given in the Supporting Information. In these cells, pristine and **IN**^**X**^**BCDT**s (**1a**–**1b**, **2a**–**2b**, and **3a**–**3b**) were employed as passivators. The schematic representation of the cell structure is provided in Figure S8. These cells were specifically designed to evaluate the passivation effects on the performance of the corresponding cells. Initially, the concentration of **IN**^**X**^**BCDT**s in the antisolvent was optimized to 0.2 wt % (vs solvent) (Table S5). Figure 8a displays the *J*–*V* curves of the best-performing devices, utilizing various passivators, while Figure 8b provides the corresponding IPCE curves. Figure 8c,d illustrates the *J*–*V* curves for both forward and reverse voltage scans of the cells. Table 2 summarizes the PV parameters of all the devices studied in this paper. The highest PCE among all PSC devices was achieved by **IN**^**Br**^**BCDT-b8** (**3a**) passivated perovskite, attaining a champion PCE of 22.20%. This superior performance included a *V*_oc_ of 1.15 V, a *J*_sc_ of 24.44 mA/cm^2^, and an *FF* of 79. In contrast, the pristine perovskite film showed a maximum PCE of 18.09%, featuring a *V*_oc_ of 1.03 V, a *J*_sc_ of 24.06 mA/cm^2^, and an *FF* of 73. PSCs fabricated with films treated by compounds **1a**–**1b**, **2a**–**2b**, and **3b**, which displayed maximum PCE values of 20.39, 19.55, 21.51, 20.61, and 21.49%, respectively. Note that all six passivators can increase the PCE of the corresponding cells, and among these, the **IN**^**Br**^**BCDT-b8** (**3a**)-treated cell has a PCE up to 22.20% (vs 18.09% for the reference cell). The successful GB passivation leads to the improved PCE of the PSCs.^67^ It is worth emphasizing that devices employing **3a**-PSK show no hysteresis (0%), in contrast to the pristine PSK, which exhibits a hysteresis index of 37% (refer to Figure 8c,d and Tables S6 and S7). Therefore, a more symmetrical distribution of ions in both scan directions leads to a higher efficiency of **3a**-PSK. Furthermore, Figure 8b (and Table S8) presents the *J*_sc_ values derived from integrated EQE measurements: 21.92 mA/cm^2^ for the reference device and 22.55, 22.44, 22.19, 22.01, 22.34, and 21.82 mA/cm^2^ for the six passivated PSCs using **IN**^**X**^**BCDT**s. These *J*_sc_ values obtained from the IPCE curves are in agreement with the *J*_sc_ values determined from the *J*–*V* curves.

**Table 2:** PV Parameters of High-Performing PSCs Using Pristine and **IN**^**X**^**BCDT** Passivators

passivating agents	*J*_sc_ (mA/cm^2^)	*V*_oc_ (V)	FF	max. PCE (%)	average PCE (%)
pristine	24.06	1.03	73	18.09	17.13 ± 0.88
**INBCDT-b8** (**1a**)	24.71	1.10	75	20.39	19.36 ± 0.70
**INBCDT-8** (**1b**)	24.57	1.09	73	19.55	18.58 ± 0.78
**IN**^**Cl**^**BCDT-b8** (**2a**)	24.41	1.13	78	21.51	20.41 ± 0.52
**IN**^**Cl**^**BCDT-8** (**2b**)	24.21	1.12	76	20.61	19.63 ± 0.82
**IN**^**Br**^**BCDT-b8** (**3a**)	24.44	1.15	79	22.20	21.37 ± 0.45
**IN**^**Br**^**BCDT-8** (**3b**)	24.17	1.14	78	21.49	20.01 ± 0.78

**Figure 9:**
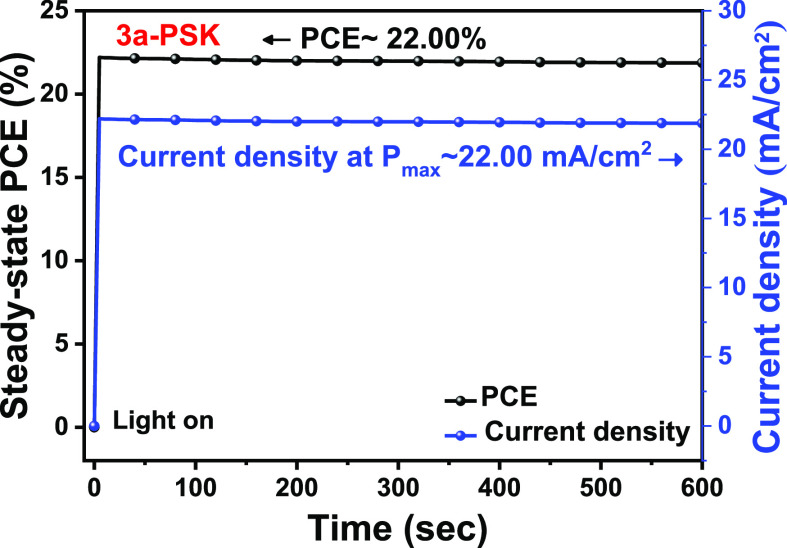
Steady-state output of PCE and current density at maximum power point (at 1.00 V) as a function of time for the champion cell (**IN**^**Br**^**BCDT-b8-PSK/3a-PSK**) under simulated 1 sun illumination.

Moreover, the photocurrent of the best-performing **3a**-PSK devices was measured to evaluate the actual power output at the maximum power point (1.00 V), revealing stabilized efficiencies of 22.00% (Figure 9). Further, the PCE distribution of the PSC device metrics based on **IN**^**Br**^**BCDT-b8** was assessed, as depicted in Figure S9. Notably, the PCE of the **IN**^**Br**^**BCDT-b8-**treated cells demonstrates an average value of 21.37 ± 0.45% for 40 cells, indicating the reproducibility of the high efficiency.

The hole mobility was measured for all the PSCs with the device structure of ITO/PEDOT:PSS/perovskite/MoO_3_/Ag, and their corresponding *I–V* characteristics are provided in Figure S10, SI. The measured hole mobilities are 1.70 × 10^–3^ and 5.26 × 10^–3^ cm^2^ V^–1^ s^–1^ for pristine and **3a**-PSK films, respectively. Concurrently, the calculated trap densities are 5.19 × 10^15^ and 3.00 × 10^15^ cm^–3^ for pristine and **3a**-PSK films, respectively. In general, the trap density is inversely proportional to hole mobility. Therefore, the higher mobility and lower trap density observed in **3a**-PSK films are advantageous for charge collection, contributing to enhanced PV performance compared to the pristine films.

**Figure 10:**
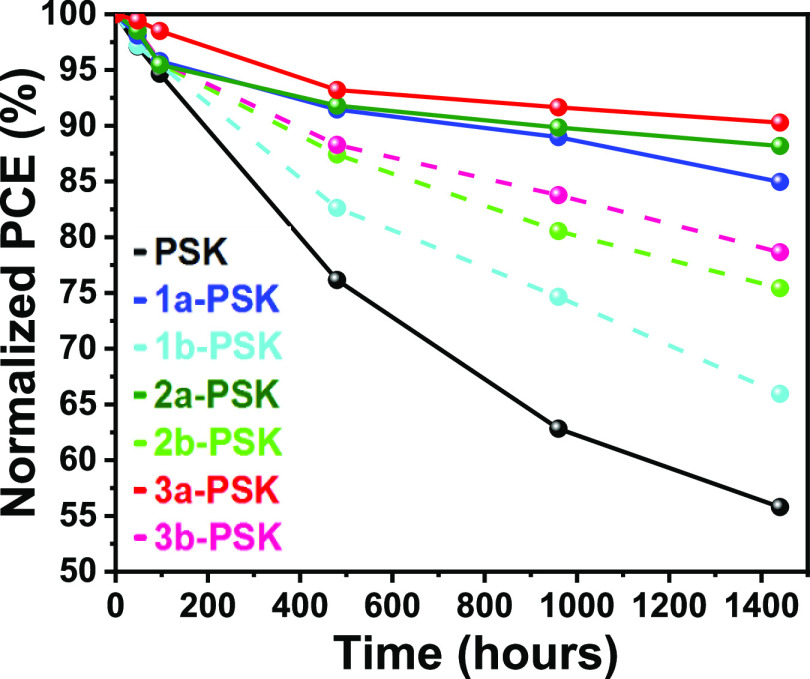
PCE decay of the PSCs based on various perovskite absorbers (stored at RT; N_2_).

The long-term stability plays a vital role in the development of PSCs.^68^ The stability test was carried out for the nonencapsulated devices within a N_2_-filled glovebox. The pristine film exhibited rapid PCE decay, losing 45% of its original PCE after 1500 h. In contrast, perovskite films passivated with **1a**–**1b**, **2a**–**2b**, and **3a**–**3b** maintained good stability, retaining 87, 67, 91, 77, 93, and 80% of their initial efficiency, respectively, as depicted in Figure 10. It is interesting to note that perovskite passivation with branched alkyl chains of **IN**^**X**^**BCDT** (**1a**-PSK, **2a**-PSK, and **3a**-PSK) has better long-term stability than those cells based on linear-chain passivated absorbers (**1b**-PSK, **2b**-PSK, and **3b**-PSK), due to the branched alkyl chain having better coverage on the perovskite surface. Furthermore, as a representative of the best-performing NFA, the devices using compound **3a** were tested for thermal stability at high temperatures (85 °C) and moisture resistivity at 50% relative humidity (Figure S11). According to Figure S11a, after 216 h of heating at 85 °C, the PCE of the reference devices loses 62% of its initial PCE, whereas the devices based on **3a**-PSK lose just 38% of their initial PCE. The reference devices decayed completely (PCE ∼ 0) after 216 h of storage in an ambient atmosphere at 50% relative humidity, whereas the devices based on **3a**-PSK retained 40% of their initial PCE (Figure S11b). These results demonstrate that the new NFA-treated PSK (**3a**-PSK) has increased long-term stability, thermal stability, and moisture resistance, which could be beneficial for high-performance and stable PSCs.

**Figure 11:**
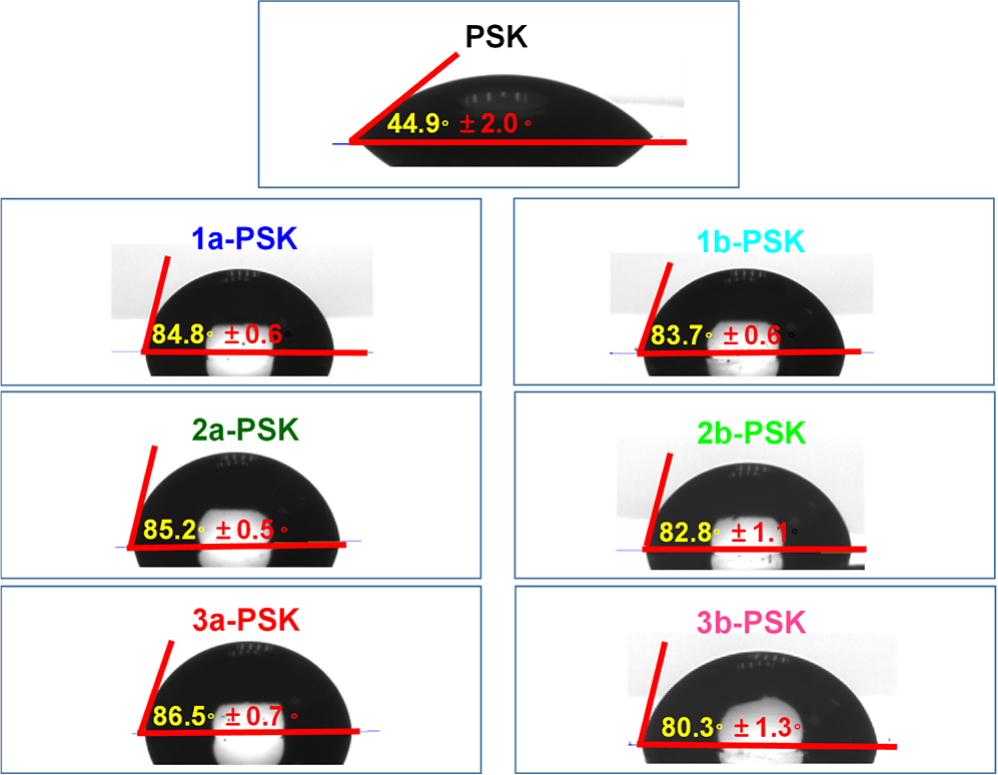
Water contact angles of pristine, **1a**–**1b**, **2a**–**2b**, and **3a**–**3b**-treated perovskite films.

It is known^69^ that defects at the GBs of perovskite materials initiate degradation when exposed to moisture and oxygen. Compounds **1a**–**1b**, **2a**–**2b**, and **3a**–**3b**-treated perovskite films are much more hydrophobic than the pristine perovskite film, as revealed by the water contact angles illustrated in Figure 11. The water contact angle of the pristine perovskite film is 44.9°, while the contact angles of the compounds **1a**–**1b**, **2a**–**2b**, and **3a**–**3b**-treated perovskite films are 84.8, 83.7, 85.2, 82.8, 86.5, and 80.3°, respectively. Among all the devices, **3a**-PSK films showed the largest water contact angle, leading to the improved stability of the devices. This improvement is ascribed to the superior quality of the perovskite film, which is characterized by a larger grain size and greater crystallinity.^70^

## 3 Conclusions

In conclusion, the study effectively showcased a straightforward and effective method to enhance both the efficiency and the stability of PSCs. This was achieved by incorporating six new organic small molecules bearing three kinds of end groups, serving as effective GB passivators in perovskite films. Specifically, A–D–A-type small molecule **IN**^**X**^**BCDT**s (compounds **1**–**3**) were designed and synthesized, featuring various end-capping units (**IN**, **IN**^**Cl**^, and **IN**^**Br**^). These compounds were strategically introduced into perovskite films during antisolvent dripping. Results from **SEM** and **XRD** measurements indicated that compound **3a-**incorporated perovskite films exhibited the largest grain size and the highest crystallinity relative to those of pristine perovskite films and those passivated with other compounds. **EDS** and **FTIR** data suggest that the presence of C=O and C≡N units in compound **3a** facilitates coordination with the under-coordinated Pb^2+^ ions, effectively passivating the defect sites within the perovskite films. **TRPL** results showed that compound **3a** promotes the transport of the holes to HTL. Overall, PSCs based on the optimized **3a**-treated perovskite absorber demonstrated outstanding performance, achieving a remarkable PCE of **22.20%** along with an **FF** of **79%**, a ***J***_**sc**_ of **24.44 mA cm**^**–2**^, and a ***V***_**oc**_ of **1.15 V**. This represents a substantial improvement compared to **18.09%** obtained with the control device utilizing a pristine perovskite absorber. The solubility factor involves an increase in the PCE of compounds **1a**, **2a**, and **3a** (2-ethylhexyl-substituted) compared to **1b**, **2b**, and **3b** (octyl-substituted). Perovskite devices treated with **IN**^**Br**^**BCDT-b8** (**3a**) maintain approximately 90% of their original PCE after 1500 h of storage inside a glovebox without packing. In contrast, the control device experiences a 45% reduction in its original PCE under identical test conditions. This work shows that GB and interface passivation by small molecular NFA are excellent approaches to achieving efficient and stable PSCs.

## 4 Experimental Section

### 4.1 Synthesis of Compound **INBCDT-b8** (**1a**)

Under anhydrous conditions, diCHO-BCDT-b8 (**10a**) (100 mg, 0.081 mmol) and 2-(3-oxo-2,3-dihydro-1*H*-inden-1-ylidene)malononitrile (11) (63 mg, 0.326 mmol) were dissolved in 30 mL CHCl_3_, 1 mL pyridine was added, and then heated to reflux for 24 h. The reaction mixture was cooled to room temperature, and 2 M HCl_(aq)_ was added slowly at 0 °C. The reaction mixture was extracted with dichloromethane and washed with water. The organic layer was dried over MgSO_4_, concentrated, and purified by column chromatography (DCM/hexane) to give a deep blue-red solid **1a** (yield = 47%). ^1^H NMR (500 MHz, CDCl_3_): δ (ppm) 8.64 (m, 2H), 8.45 (s, 2H), 8.03 (m, 2H), 7.83–7.81 (m, 4H), 7.63 (s, 2H), 7.09–6.82 (m, 18H), 3.81–3.76 (m, 8H), 1.71–1.66 (m, 4H), 1.49–1.35 (m, 16H), 1.29–1.27 (m, 16H), 0.90–0.87 (m, 24H); ^13^C NMR (125 MHz, CDCl_3_): δ (ppm) 187.96, 164.14, 159.31, 158.63, 155.53, 145.34, 140.63, 139.71, 139.45, 138.04, 136.50, 135.75, 134.42, 133.84, 128.59, 124.98, 123.66, 119.91, 119.07, 114.87, 114.67, 114.61, 70.55, 67.14, 61.40, 39.40, 30.53, 30.48, 29.09, 23.87, 23.00, 14.03, 11.12, 11.09; HRMS (MALDI, [M]^+^) calcd. for C_100_H_98_N_4_O_6_S_4_, 1578.6369; found, 1578.6364.

### 4.2 Synthesis of Compound **INBCDT-8** (**1b**)

Followed the procedure for preparing **1a** using **10b** and **11**. The crude product was purified by column chromatography (DCM/hexane) to give a deep blue-red solid **1b** (yield = 50%). ^1^H NMR (500 MHz, CDCl_3_): δ (ppm) 8.64 (m, 2H), 8.41 (s, 2H), 8.02 (m, 2H), 7.85–7.79 (m, 4H), 7.63 (s, 2H), 7.14–6.81 (m, 18H), 3.93–3.86 (m, 8H), 1.77–1.71 (m, 8H), 1.45–1.39 (m, 8H), 1.30–1.25 (m, 32H), 0.87–0.84 (m, 12H); ^13^C NMR (125 MHz, CDCl_3_): δ (ppm) 187.96, 164.14, 159.04, 158.46, 155.54, 145.26, 140.70, 139.69, 139.44, 138.11, 136.50, 135.70, 134.44, 133.96, 128.75, 124.98, 123.62, 120.02, 119.10, 114.91, 114.68, 114.61, 68.18, 67.16, 61.41, 31.82, 29.36, 29.26, 29.23, 26.05, 22.62, 14.05; HRMS (MALDI, [M]^+^) calcd. for C_100_H_98_N_4_O_6_S_4_, 1578.6369; found, 1578.6364.

### 4.3 Synthesis of Compound **IN**^**Cl**^**BCDT-b8** (**2a**)

Under anhydrous conditions, diCHO-BCDT-b8 (**10a**) (100 mg, 0.081 mmol) and 2-(5,6-dichloro-3-oxo-2,3-dihydro-1*H*-inden-1-ylidene)malononitrile (12) (86 mg, 0.326 mmol) were dissolved in 30 mL of CHCl_3_, 1 mL of pyridine was added, and then heated to reflux for 24 h. The reaction mixture was cooled to room temperature, and 2 M HCl_(aq)_ was added slowly at 0 °C. The reaction mixture was extracted with dichloromethane and washed with water. The organic layer was dried over MgSO_4_, concentrated, and purified by column chromatography (DCM/hexane) to give a deep blue solid **2a** (yield = 55%). ^1^H NMR (500 MHz, CDCl_3_): δ (ppm) 8.78 (s, 2H), 8.66 (s, 2H), 7.95 (s, 2H), 7.65 (s, 2H), 7.18 (m, 8H), 6.85 (m, 10H), 3.83–3.77 (m, 8H), 1.71–1.66 (m, 4H), 1.47–1.36 (m, 16H), 1.30–1.28 (m, 16H), 0.91–0.87 (m, 24H); ^13^C NMR (125 MHz, CDCl_3_): δ (ppm) 185.77, 165.48, 159.52, 159.28, 156.79, 145.96, 140.73, 139.97, 139.52, 138.49, 138.20, 136.43, 135.66, 133.37, 128.59, 126.67, 124.98, 119.80, 119.41, 114.92, 114.27, 113.98, 70.58, 67.96, 61.49, 39.38, 30.52, 30.49, 29.08, 23.86, 23.0, 14.03, 11.11, 11.09; HRMS (MALDI, [M]^+^) calcd. for C_100_H_94_Cl_4_N_4_O_6_S_4_, 1714.4810; found, 1714.4805.

### 4.4 Synthesis of Compound **IN**^**Cl**^**BCDT-8** (**2b**)

Followed the procedure for preparing **2a** using **10b** and **12**. The crude product was purified by column chromatography (DCM/hexane) to give a deep blue solid **2b** (yield = 56%). ^1^H NMR (500 MHz, CDCl_3_): δ (ppm) 8.73 (s, 2H), 8.58 (s, 2H), 7.96 (s, 2H), 7.66 (s, 2H), 7.23 (m, 8H), 6.87 (m, 10H), 3.93–3.87 (m, 8H), 1.77–1.72 (m, 8H), 1.43–1.39 (m, 8H), 1.30–1.26 (m, 32H), 0.87–0.85 (m, 12H); ^13^C NMR (125 MHz, CDCl_3_): δ (ppm) 185.80, 165.50, 159.51, 159.02, 156.75, 145.87, 140.70, 139.95, 139.52, 138.54, 138.24, 136.39, 135.69, 133.44, 128.64, 126.70, 124.95, 119.89, 119.47, 114.92, 114.31, 114.01, 68.18, 67.97, 61.49, 31.81, 30.90, 29.36, 29.23, 26.05, 25.61, 22.63, 14.07; HRMS (MALDI, [M]^+^) calcd. for C_100_H_94_Cl_4_N_4_O_6_S_4_, 1714.4810; found, 1714.4805.

### 4.5 Synthesis of Compound **IN**^**Br**^**BCDT-b8** (**3a**)

Under anhydrous conditions, diCHO-BCDT-b8 (**10a**) (100 mg, 0.081 mmol) and 2-(5,6-dibromo-3-oxo-2,3-dihydro-1*H*-inden-1-ylidene)malononitrile (13) (115 mg, 0.326 mmol) were dissolved in 30 mL of CHCl_3_, 1 mL of pyridine was added, and then heated to reflux for 24 h. The reaction mixture was cooled to room temperature, and 2 M HCl_(aq)_ was added slowly at 0 °C. The reaction mixture was extracted with dichloromethane and washed with water. The organic layer was dried over MgSO_4_, concentrated, and purified by column chromatography (DCM/hexane) to give a deep blue-green solid **3a** (yield = 52%). ^1^H NMR (500 MHz, CDCl_3_): δ (ppm) 8.78 (m, 4H), 8.10 (s, 2H), 7.65 (s, 2H), 7.17 (m, 8H), 6.85 (m, 10H), 3.81–3.78 (m, 8H), 1.71–1.66 (m, 4H), 1.48–1.36 (m, 16H), 1.29–1.27 (m, 16H), 0.90–0.87 (m, 24H); ^13^C NMR (125 MHz, CDCl_3_): δ (ppm) 185.73, 165.40, 159.32, 156.89, 156.65, 146.01, 140.85, 140.19, 138.73, 138.56, 136.50, 135.98, 133.43, 132.87, 132.29, 129.67, 128.61, 128.02, 119.73, 119.21, 114.95, 114.34, 114.02, 70.60, 67.97, 61.50, 39.40, 30.54, 30.51, 29.10, 23.88, 23.01, 14.05, 11.14, 11.11; HRMS (MALDI, [M]^+^) calcd. for C_100_H_94_Br_4_N_4_O_6_S_4_, 1890.2790; found, 1890.2784.

### 4.6 Synthesis of Compound **IN**^**Br**^**BCDT-8** (**3b**)

Followed the procedure for preparing **3a** using **10b** and **13**. The crude product was purified by column chromatography (DCM/hexane) to give a deep blue-green solid **3b** (yield = 58%). ^1^H NMR (500 MHz, CDCl_3_): δ (ppm) 8.73 (m, 4H), 8.10 (s, 2H), 7.66 (s, 2H), 7.21 (m, 8H), 6.87 (m, 10H), 3.93–3.85 (m, 8H), 1.77–1.72 (m, 8H), 1.43–1.39 (m, 8H), 1.30–1.26 (m, 32H), 0.87–0.85 (m, 12H); ^13^C NMR (125 MHz, CDCl_3_): δ (ppm) 185.53, 165.19, 159.10, 157.07, 156.26, 146.04, 140.90, 140.32, 138.53, 136.57, 135.80, 133.53, 132.93, 132.30, 129.55, 128.65, 127.85, 119.48, 118.90, 114.94, 114.27, 113.90, 68.16, 67.76, 61.42, 31.81, 29.67, 29.36, 29.23, 26.05, 22.62, 14.07; HRMS (MALDI, [M]^+^) calcd. for C_100_H_94_Br_4_N_4_O_6_S_4_, 1890.2790; found, 1890.2784.

## Supporting Information Available

Experimental details, TGA, DPV curves, UPS, optical Tauc plots, and other characterization data (PDF)
