# Supplementary material for: Bicyclopentadithiophene-Based Organic Semiconductor for Stable and High-Performance Perovskite Solar Cells Exceeding 22%
Source: ACS Appl Mater Interfaces. 2024 Jan 26;16(5):6162–75. doi: 10.1021/acsami.3c15774 (PMC10859901; doi:10.1021/acsami.3c15774)
Supplement: Supplementary file 1 — am3c15774_si_001.pdf [file am3c15774_si_001.pdf]

Supporting Information

Bicyclopentadithiophene-Based Organic Semiconductor for Stable and High-Performance Perovskite Solar Cells Exceeding 22%

Arulmozhi Velusamy, [a,‡] *Shakil N. Afraj,* [a,‡] *Yu-Sheng Guo,* [a] *Jen-Shyang Ni,* [b] *Hung-Lin Huang,* [a] *Ting-Yu Su,* [a] *Yamuna Ezhumalai,* [a] *Cheng-Liang Liu,* [c] *Chien-Hung Chiang,* *[a] *Ming-Chou Chen* *[a] *and Chun-Guey Wu* *[a]

[a] Department of Chemistry, National Central University, Taoyuan 32001, Taiwan.

[b] Department of Chemical and Materials Engineering, National Kaohsiung University of Science and Technology, Kaohsiung 80778, Taiwan.

[c] Department of Materials Science and Engineering, National Taiwan University, Taipei 10617, Taiwan.

‡A. Velusamy and S. N. Afraj contributed equally to this work.

*Chien-Hung Chiang. E-mail: chiang95@cc.ncu.edu.tw

*Ming-Chou Chen. E-mail: mcchen@ncu.edu.tw

*Chun-Guey Wu. E-mail: t610002@ncu.edu.tw

Table of Contents

Materials

Characterization

Device fabrication

Synthesis of intermediates

Scheme S1. Synthesis of compound **6**

Scheme S2. Synthesis of compound **8a-b**

Scheme S3. Synthesis of compounds **9a-b** and **10a-b**

Scheme S4. Synthesis of compound **13**

Figure S1. Chemical structure of **IN^{X}BCDT** non-fullerene acceptors (NFAs) explored as an anti-solvent additive in the fabrication of perovskite solar cells (PSCs).

Figure S2. TGA curves of **IN^{X}BCDT** compounds (**1-3**)

Figure S3. DPV curves of **IN^{X}BCDT** (**1-3**) in *o*-dichlorobenzene

Figure S4. The UPS of the perovskite made by using pure CB (pristine perovskite), 0.2 wt% compounds **1-3** treated perovskite

Figure S5. The optical Tauc plots of the pristine perovskite and perovskite films treated with 0.2 wt% compounds **1-3**

Table S1. The energy level of the pristine perovskite and perovskite films treated with 0.2 wt% compounds **1-3**

Figure S6. UV-Vis absorption spectra of pristine perovskite and perovskite films treated with 0.2 wt% compounds **1-3**

Figure S7. FTIR spectra of compound **3a** and **3a**+PbI_2

Table S2. Steady-state PL spectra of perovskite films on glass and with Spiro-OMeTAD

Table S3. TRPL spectra of perovskite films on glass.

Table S4. TRPL spectra of perovskite films with Spiro-OMeTAD overlayer

Table S5. The device performance parameters of PSCs based on various concentration of **IN^{Br}BCDT-b8**.

Figure S8. (a) Device architecture of PSCs and (b) Schematic diagram of grain boundary passivation by **IN^{Br}BCDT-b8**

Table S6. The *I-V* curves of the cell based on IN^{Br}**BCDT-b8** (**3a**) treated perovskite measured with both voltage scan directions

Table S7. The *I-V* curves of the cell based on **pristine** perovskite measured with both voltage scan directions

Table S8. The integrated photocurrent density from IPCE and short-circuit current density from *J-V* of the reference cell and cells based on compounds **1-3**-treated perovskite films

Figure S9. The efficiency distribution for perovskite solar cells based on **IN^{Br}BCDT-b8** (**3a**) treated perovskite absorbers (40 devices, 21.37 ± 0.45%)

Figure S10. Hole-only device and current-voltage measurement curves of the (a) Pristine perovskite and (b) **IN^{Br}BCDT-b8-PSK** (**3a**-PSK) films

Figure S11. The PCE decay of the PSCs based on PSK and **3a**-PSK absorbers a) By storing at 85 °C in a glove box without encapsulation, b) By storing at 50% relative humidity in air with encapsulation.

Figure S12-S52. ^{1}H NMR, ^{13}C NMR, and HRMS spectra of synthesized compounds

Materials

All chemical reagents were used as received, including dimethyl sulfoxide (DMSO; 99.5%, Sigma-Aldrich), N,N-dimethylformamide (DMF; 99.99%, Sigma-Aldrich), PbI_2 (99.999%, Alfa-aesar), isopropanol (99.5%, Sigma-Aldrich), formamidinium iodide, methylammonium bromide, and methylammonium iodide (FAI, MABr, and MAI; Ossila), chlorobenzene (99.99%, Sigma-Aldrich), Spiro-OMeTAD (Lumtec), acetonitrile (99.9%, Sigma-Aldrich), lithium bis(trifluoromethylsulfonyl)imide (Li-TFSI; 99.95%, Sigma-Aldrich), 4-tertbutylpyridine (*t*BP: 99.9%, Sigma-Aldrich), acetone, ethanol (AR Beijing Chemical Works), and, MoO_3 (99.999%, Alfa-aesar). Reaction solvents (toluene, ether, and THF) were distilled under nitrogen from sodium/benzophenone ketyl, and halogenated solvents were distilled from CaH_2. 1-bromo-4-((alkyl)oxy)benzene (**a** and **b**)[1] and compounds **4**,[2] **5**,[3] **11**,[4] **12**,[5] and **14**[6] were synthesized by the reported procedures.

Characterization

^{1}H and ^{13}C NMR spectra were recorded using a Bruker 500 or a 300 instrument, with reference to solvent signals. Differential scanning calorimetry (DSC) was carried out on a Mettler DSC 822 instrument at a scan rate of 10 K/min. Thermogravimetric analysis (TGA) was performed on a Perkin Elmer TGA-7 thermal analysis system using dry nitrogen as a carrier gas at a flow rate of 40 mL/min. UV–Vis absorption and fluorescence spectra were obtained in the indicated solvents

at room temperature using JASCO V-530 and Hitachi F-4500 spectrometers, respectively. Differential pulse voltammetry experiments were performed with a conventional three-electrode configuration (a platinum disk working electrode, an auxiliary platinum wire electrode, and a non-aqueous Ag reference electrode, with a supporting electrolyte of 0.1 M tetrabutylammonium hexafluorophosphate (dry $TBAPF_6$) in the specified dry solvent, using a CHI621C Electrochemical Analyzer (CH Instruments). Under N_2, the anhydrous OSC material was dissolved in above 0.1 M *o*-$C_6H_4Cl_2$ solution to prepare a 10^{-3} M test solution. In each DPV experiment, 5 mL of the test solution was scanned together with Fc/Fc^+ (also 10^{-3} M; as internal standard) under N_2. Electrochemical potentials were referenced to an Fc/Fc^+ internal standard (at +0.64 V *versus* NHE). Mass spectrometric data were obtained with a JMS-700 HRMS instrument. Grazing incidence XRD data were collected in the 2θ range of 5−50 degree on a Bruker powder diffractometer (D8 Discover) using Cu $K\alpha_1$ radiation equipped with a 2D detector. UPS and X-ray photoelectron spectroscopy spectra were obtained from a Thermo VG-Scientific/VG-Sigma Probe spectrometer. UV/Vis and PL spectra were recorded using a Cary 300 Bio spectrometer and a Hitachi F-7000 fluorescence spectrophotometer, respectively, at room temperature. The nanosecond TRPL spectra were recorded with an optical microscope-based system (UniRAM, Protrustech with the custom designed light path). The average power, wavelength, pulse duration, and repetition rate of the excitation are 20 μW, 405 nm, 150 ps, and 20 MHz, respectively. SEM was performed with a Hitachi S-800 microscope at 15 KV. Samples for SEM imaging were mounted on a metal stub with a piece of conducting tape and then coated with a thin layer of gold film to avoid charging. The cross-sectional SEM image was used for estimating the film thickness and observing the interface contact between each layer of the cell. The contact angle was measured with a home-made set-up (Grandhand Ctag01, Taiwan) using water as a probe solvent. The contact angle is

determined using the image of a sessile drop at the points of the intersection between the drop contour and the projection of the surface. Fourier transform infrared (FTIR) spectra were recorded for IN^{X}BCDT derivatives and IN^{X}BCDT derivatives+PbI_2 film in KBr using a Bio-Rad 155 FTIR spectrometer at ambient temperature with a resolution of 4 cm^{-1}.

Device fabrication

Regular (n-i-p) mesoporous PSC devices with the cell architecture of FTO (150 nm)/c-TiO_2/m-TiO_2/PSK (ca. 720-740 nm)/Spiro-OMeTAD (ca. 200 nm)/MoO_3 (5 nm)/Ag (100 nm) were fabricated. Fluorine-doped tin oxide (FTO) glass was washed in a detergent solution for 30 min with sonication, followed by consecutive rinsing with water, ethanol, and acetone. A dense compact layer of TiO_2 (30 nm, c-TiO_2) was deposited onto the FTO by pyrolysis using a 0.25 M TTIP (titanium isopropoxide) EtOH solution (Aldrich) at 180 ^{o}C. To prepare the mp-TiO_2 layer, the TiO_2 nanoparticles (30-NRT, Dyesol) paste diluted in ethanol (1:5, weight ratio) was spin-coated onto the substrate at 2000 rpm for 10 s and calcined at 550 ^{o}C for 30 min, which leads to an around 200 nm thick mp-TiO_2 mesoporous film. A perovskite absorber layer was deposited on top of ETL using a one-step, anti-solvent dripping method from its precursor solution. The PSK precursor solution was prepared by mixing FAI, MABr, PbI_2, and $PbBr_2$ in DMF/DMSO (6:4, V/V) to form a 1.3 M $(FAPbI_3)_{0.85}(MAPbBr_3)_{0.15}$ solution. A 40 μL PSK precursor solution was coated on the ETL with a spin program of 1000 rpm for 10 s, then 7000 rpm for 15 s; in the last 3 s, 100 μL chlorobenzene (CB) or **IN^{X}BCDT**s;**1-3** containing CB solution with various concentrations were dripped on the perovskite precursor film to induce the crystallization. The resulting $(FAPbI_3)_{0.85}(MAPbBr_3)_{0.15}$ (pristine PSK or **IN^{X}BCDT** passivated PSK) film was heated at 150 ^{o}C for 10 min. Subsequently, doped Spiro-OMeTAD HTL was spin-coated on perovskite

film and exposure to air for 2 hours, followed by the vacuum deposition of the 5 nm MoO_3 hole blocking layer and 100 nm Ag electrode. The deposition of the perovskite active layer and HTL was performed in an N_2-filled glove box. The cells were further optimized by depositing an approximately 100 nm-thick LiF film on the back of the FTO glass as an anti-reflection coating. The physicochemical properties and photovoltaic performance of the PSCs were measured using the same methods as those previously reported.[7, 8]

Synthesis of intermediates

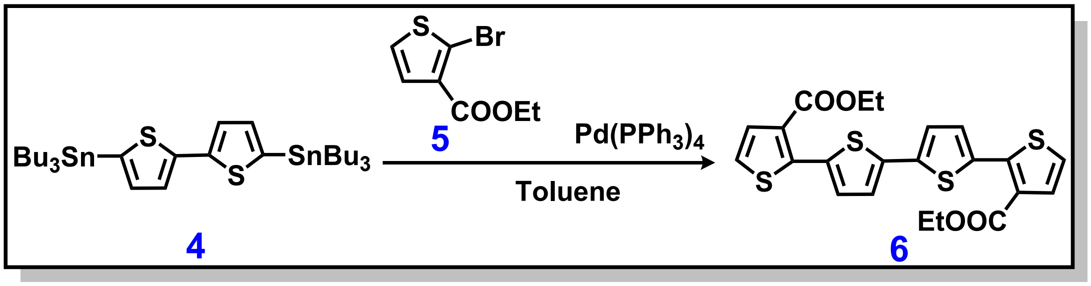

Scheme S1. Synthesis of Compound **6**.

Synthesis of diethyl [2,2':5',2'':5'',2'''-quaterthiophene]-3,3'''-dicarboxylate (6): Under anhydrous and deoxygenated conditions $Pd(PPh_3)_4$ (0.15 g, 0.13 mmol) was added to the solution of 5,5'-bis(tributylstannyl)-2,2'-bithiophene (4) (2.0 g, 2.7 mmol) and ethyl 2-bromothiophene-3-carboxylate (5) (1.88 g, 8.1 mmol) in toluene (20 ml). The resulting mixture was refluxed for two days under nitrogen. After cooling to room temperature, the solvent was evaporated, and the crude material was purified by column chromatography (15% ethyl ecetate/hexane) to give product **6** as a yellow-orange solid, (yield = 75 %). ^{1}H NMR (500 MHz, $CDCl_3$): δ (ppm) 7.49 (d, J = 5.5 Hz, 2H), 7.39 (d, J = 4 Hz, 2H), 7.19 (d, J = 5.5 Hz, 2H), 7.17 (d, J = 4 Hz, 2H), 4.33 (q, J = 7 Hz, 4H), 1.34 (t, J = 7.3 Hz, 6H); ^{13}C NMR (125 MHz, $CDCl_3$): δ (ppm) 163.14, 142.75, 138.96,

133.23, 130.63, 130.07, 127.85, 124.02, 123.82, 60.85, 14.26; HRMS (MALDI, [M]$^+$) calcd. for $C_{22}H_{18}O_4S_4$: 474.0088, Found: 474.0082.

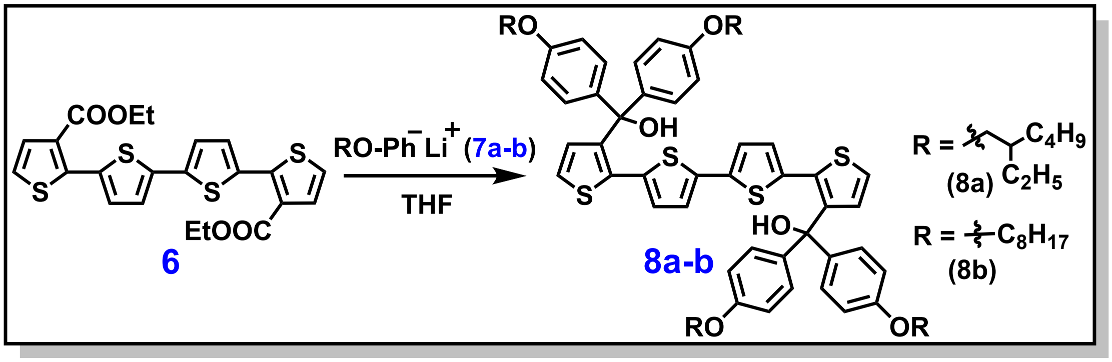

Scheme S2. Synthesis of Compound **8a-b**.

Synthesis of [2,2':5',2'':5'',2'''-quaterthiophene]-3,3'''-diylbis(bis(4-((2-ethylhexyl)oxy)phenyl)methanol) BCDT-OH-b8 (8a): 2.5 M *n*-BuLi (3.4 mL in Hexanes, 8.2 mmol) was slowely added to 1-bromo-4-((2-hexyldecyl)oxy)benzene (2.39 g, 8 mmol) at -78 °C in THF (30 ml) under anhydrous condition, the mixture was stirred for one hour and then it was warmed to 0 °C to remove the formed C_4H_9Br in the reaction by vacuum, then THF (30 mL) was added and transferred slowly to reaction flask containing the solution of diethyl [2,2':5',2'':5'',2'''-quaterthiophene]-3,3'''-dicarboxylate (6) (0.5 g, 1 mmol) in THF (70 mL) under anhydrous condition at -78 ℃. After the addition, the reaction mixture was slowly warmed to room temperature and stirred for 12 hours at same temperature. Reaction was quenched by slow addition of deionized water and, THF was removed by rotary evaporator and reaction mixture was extracted by diethyl ether and washed with water. Organic layer was dried over $MgSO_4$ and concentrated to give crude reaction mixture which is then purified by column chromatography (7% ethyl ecetate/hexane) to give yellow solid, (yield = 60%). ^{1}H NMR (300 MHz, $CDCl_3$): δ (ppm) 7.15 (d,

J = 8.7 Hz, 8H), 7.10 (d, *J* = 5.1 Hz, 2H), 6.84-6.80 (m, 10H), 6.52 (d, *J* = 3.6 Hz, 2H), 6.43 (d, *J* = 5.1 Hz, 2H), 3.84 (d, *J* = 5.7 Hz, 8H), 3.30 (s, 2H), 1.77-1.69 (m, 4H), 1.54-1.39 (m, 16H), 1.37-1.30 (m, 16H), 0.95-0.89 (m, 24H); ^{13}C NMR (125 MHz,$CDCl_3$): δ (ppm) 158.65, 146.17, 139.70, 138.31, 134.67, 131.69, 131.05, 129.17, 128.75, 123.91, 123.48, 113.84, 80.12, 70.58, 39.42, 30.56, 29.12, 23.89, 23.05, 14.08, 11.13; HRMS (MALDI, $[M]^+$) calcd. for $C_{74}H_{94}O_6S_4$: 1206.5933, Found: 1206.5928.

Synthesis of [2,2':5',2'':5'',2'''-quaterthiophene]-3,3'''-diylbis(bis(4-(octyloxy)phenyl)methanol) BCDT-OH-8 (8b): Followed the procedure as compound **8a.** The crude reaction mixture was purified by column chromatography (7% ethyl acetate/hexane) to give yellow solid, (yield = 64%). ^{1}H NMR (500 MHz, $CDCl_3$): δ (ppm) 7.14 (d, *J* = 9 Hz, 8H), 7.10 (d, *J* = 5.5 Hz, 2H), 6.82 (d, *J* = 8.5 Hz, 8H), 6.80 (d, *J* = 3.5 Hz, 2H), 6.50 (d, *J* = 4 Hz, 2H), 6.41 (d, *J* = 5.5 Hz, 2H), 3.94 (t, *J* = 6.8 Hz, 8H), 3.31 (s, 2H), 1.80-1.74 (m, 8H), 1.48-1.43 (m, 8H), 1.32-1.26 (m, 32H), 0.90-0.87 (m, 12H); ^{13}C NMR (125 MHz,$CDCl_3$): δ (ppm) 158.29, 146.03, 139.66, 138.19, 134.55, 131.60, 131.02, 129.09, 128.70, 123.82, 123.38, 113.69, 79.98, 67.91, 31.76, 29.32, 29.24, 29.18, 26.02, 22.60, 14.05; HRMS (MALDI, $[M]^+$) calcd. for $C_{74}H_{94}O_6S_4$: 1206.5933, Found: 1206.5928.

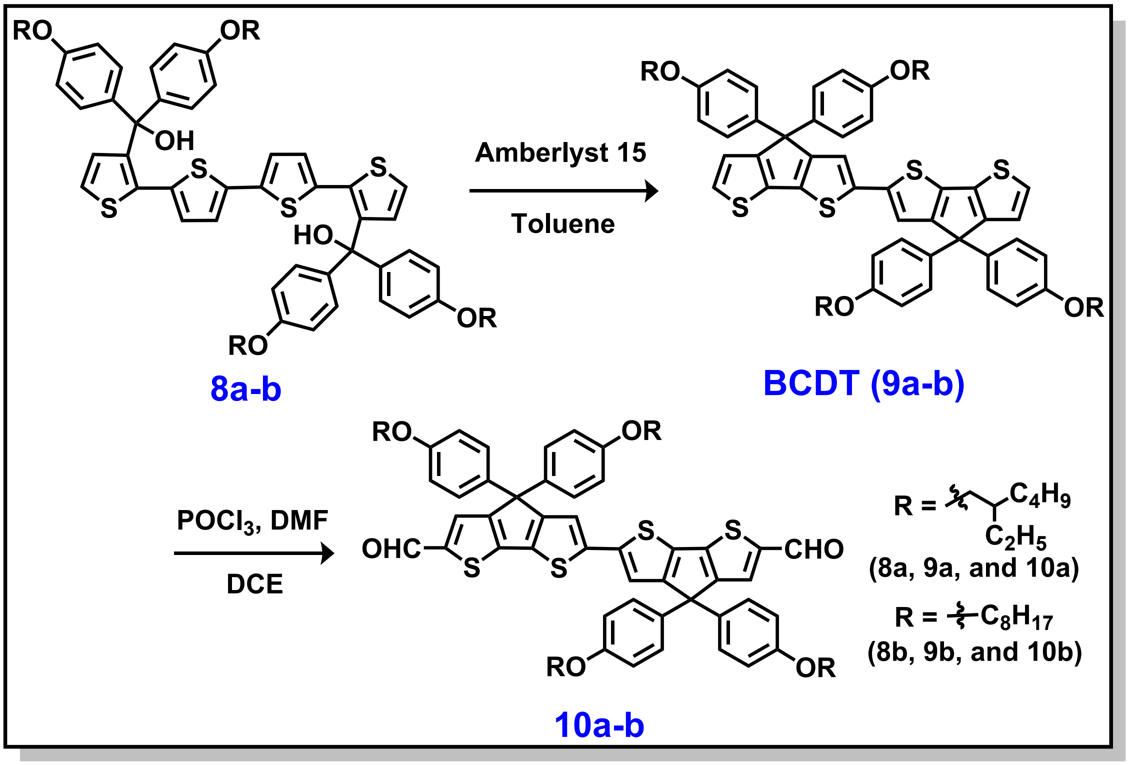

Scheme S3. Synthesis of Compounds **9a-b** and **10a-b**.

Synthesis of 4,4,4',4'-tetrakis(4-((2-ethylhexyl)oxy)phenyl)-4H,4'H-2,2'-bi(cyclopenta[2,1-b:3,4-b']dithiophene) BCDT-b8 (9a): BCDT-OH-b8 (8a) (0.76 g, 0.6 mmol) was added to reaction flask containing Amberlyst-15 (0.8 g, 2.4 mmol) and toluene (30 ml) under anhydrous condition and refluxed for 4 hours. After completion, the reaction mixture was cooled down to room temperature and filtered through suction and liquid was collected and removed by a rotary evaporator. The crude reaction mixture was purified by column chromatography (20% dichloromethane/hexane) to give the product as orange liquid (yield = 30%). ^{1}H NMR (500 MHz, $CDCl_3$): δ (ppm) 7.19 (d, J = 5 Hz, 2H), 7.16 (d, J = 9 Hz, 8H), 7.07 (s, 2H), 7.01 (d, J = 4.5 Hz, 2H), 6.78 (d, J = 8.5 Hz, 8H), 3.80 (d, J = 5.5 Hz, 8H), 1.72-1.67 (m, 4H), 1.53-1.39 (m, 16H), 1.32-1.28 (m, 16H), 0.93-0.89 (m, 24H); ^{13}C NMR (125 MHz,$CDCl_3$): δ (ppm) 158.87, 158.33, 158.09, 138.65, 136.08, 135.34, 134.65, 128.68, 125.82, 123.26, 119.13, 114.33, 70.38, 61.23,

39.42, 31.61, 30.55, 29.72, 29.10, 23.88, 23.05, 22.67, 14.11, 14.07, 11.43, 11.11; HRMS (MALDI, $[M]^+$) calcd. for $C_{74}H_{90}O_4S_4$: 1170.5722, Found: 1170.5716.

Synthesis of 4,4,4',4'-tetrakis(4-(octyloxy)phenyl)-4H,4'H-2,2'-bi(cyclopenta[2,1-b:3,4-b']dithiophene) BCDT-8 (9b): Followed the procedure as compound **9a.** The crude reaction mixture was purified by column chromatography (25% dichloromethane/hexane) to give product as orange liquid (yield = 31%). ^{1}H NMR (300 MHz, $CDCl_3$): δ (ppm) 7.18 (d, J = 5.1 Hz, 2H), 7.14 (d, J = 8.7 Hz, 8H), 7.06 (s, 2H), 7.01 (d, J = 5.1 Hz, 2H), 6.77 (d, J = 8.7 Hz, 8H), 3.90 (t, J = 6.5 Hz, 8H), 1.79-1.70 (m, 8H), 1.44 (m, 8H), 1.28-1.27 (m, 32H), 0.92-0.87 (m, 12H); ^{13}C NMR (125 MHz,$CDCl_3$): δ (ppm) 158.77, 158.04, 138.59, 136.03, 135.37, 134.63, 128.66, 125.78, 123.22, 119.08, 114.28, 67.90, 61.17, 34.64, 31.90, 31.78, 31.56, 29.68, 29.32, 29.26, 29.20, 26.04, 25.26, 22.62, 14.05; HRMS (MALDI, $[M]^+$) calcd. for $C_{74}H_{90}O_4S_4$: 1170.5722, Found: 1170.5716.

Synthesis of 4,4,4',4'-tetrakis(4-((2-ethylhexyl)oxy)phenyl)-4H,4'H-[2,2'-bi(cyclopenta[2,1-b:3,4-b']dithiophene)]-6,6'-dicarbaldehyde diCHO-BCDT-b8 (10a): $POCl_3$ was added dropwise (0.69 mL, 7.5 mmol) to the solution of DMF (1.55 mL, 19 mmol) in DCE at 0 °C and stirred for one hour at same temperature. To this solution, BCDT-b8 (9a) (0.22 g, 0.19 mmol) in DCE was added and refluxed for 16 hours. The reaction mixture was cooled to room temperature and a slowly saturated solution of $NaHCO_3$ was added at 0 °C and warmed to RT. The reaction mixture was extracted with ethyl acetate and washed with water, organic layer was concentrated and crude residue was purified by column chromatography (10% ethyl acetate/hexane), and washed with methanol/hexane to give product as red solid, (yield = 85%); ^{1}H NMR (500 MHz, $CDCl_3$): δ (ppm)

9.79 (s, 2H), 7.61 (s, 2H), 7.14 (s, 2H), 7.12 (d, *J* = 8.5 Hz, 8H), 6.79 (d, *J* = 9 Hz, 8H), 3.78 (d, *J* = 5.5 Hz, 8H), 1.70-1.65 (m, 4H), 1.48-1.34 (m, 16H), 1.31-1.27 (m, 16H), 0.91-0.86 (m, 24H); ^{13}C NMR (125 MHz, $CDCl_3$): δ (ppm) 182.41, 162.94, 158.75, 158.56, 146.19, 144.41, 142.33, 134.41, 133.88, 131.57, 128.52, 120.01, 114.62, 70.47, 61.59, 39.39, 30.53, 29.71, 29.09, 23.87, 23.04, 14.08, 11.11; HRMS (MALDI, $[M]^+$) calcd. for $C_{76}H_{90}O_6S_4$: 1226.5620, Found: 1226.5615.

Synthesis of 4,4,4',4'-tetrakis(4-(octyloxy)phenyl)-4H,4'H-[2,2'-bi(cyclopenta[2,1-b:3,4-b']dithiophene)]-6,6'-dicarbaldehyde diCHO-BCDT-8 (10b): Followed the procedure as compound **10a.** The crude residue was purified by column chromatography (10% ethyl ecetate/hexane), and washed with methanol/hexane to give product as red solid, (yield = 84%); ^1H NMR (500 MHz, $CDCl_3$): δ (ppm) 9.79 (s, 2H), 7.62 (s, 2H), 7.14 (s, 2H), 7.12 (d, *J* = 9 Hz, 8H), 6.79 (d, *J* = 9 Hz, 8H), 3.90 (t, *J* = 6.5 Hz, 8H), 1.77-1.71 (m, 8H), 1.45-1.39 (m, 8H), 1.31-1.26 (m, 32H), 0.89-0.86 (m, 12H); ^{13}C NMR (125 MHz, $CDCl_3$): δ (ppm) 182.33, 162.81, 158.45, 146.11, 144.34, 142.24, 134.36, 133.90, 131.49, 128.48, 119.94, 114.55, 67.98, 61.51, 31.74, 31.53, 29.64, 29.27, 29.19, 29.16, 25.99, 22.59, 14.05, 14.02; HRMS (MALDI, $[M]^+$) calcd. for $C_{76}H_{90}O_6S_4$: 1226.5620 Found: 1226.5615.

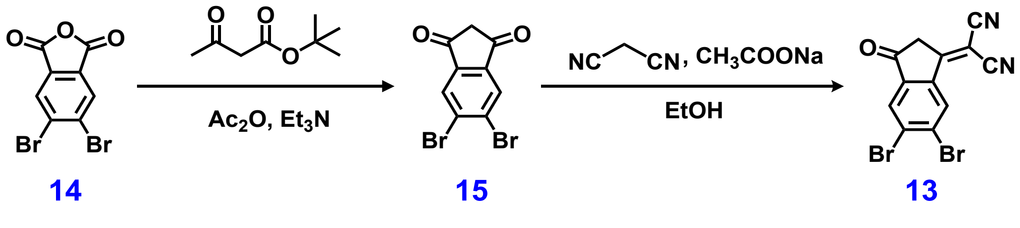

Scheme S4. Synthesis of compound **13**.

Synthesis of 5,6-dibromo-1H-indene-1,3(2H)-dionee (**15**): 4,5-dibromophthalic anhydride (1g, 3.27 mmol) was dissolved in the mixture of acetic anhydride (4 mL) and triethylamine (2 mL). Then, tert-butyl acetoacetate (0.517g, 6.32 mmol) was slowly added to the mixture and the reaction was carried out overnight at room temperature. Later, the reaction was quenched with ice and concentrated hydrochloric acid. The obtained mixture was then stirred and heated at 70~80 °C for 1 hour. The reaction mixture was cooled to room temperature and filtered under reduced pressure to obtain a solid product (0.89g, 89%). ^{1}H NMR (300 MHz, $CDCl_3$): δ (ppm) 8.25 (s, 2H), 3.28 (s, 2H).

2-(5,6-dibromo-3-oxo-2,3-dihydro-1H-inden-1-ylidene)malononitrile (**13**): 5,6-dibromo-1,3-indandione (**15**) (1g, 3.29 mmol) and malononitrile (0.434 mg, 6.58 mmol) was dissolved in ethanol (20 mL). After the addition of anhydrous sodium acetate (0.405g, 4.94 mmol), the mixture was stirred at room temperature for 1 hour and then heated for 3 hours. Next, the reaction mixture was slowly poured into the mixture of water and ethanol (20 mL). Finally, acidified with aqueous hydrochloric acid and precipitated solid was filtered and washed with hexane to get compound **13** (0.903g, 78%). ^{1}H NMR (300 MHz, $CDCl_3$): δ (ppm) 8.90 (s, 1H), 8.23 (s, 1H), 3.76 (s, 2H).

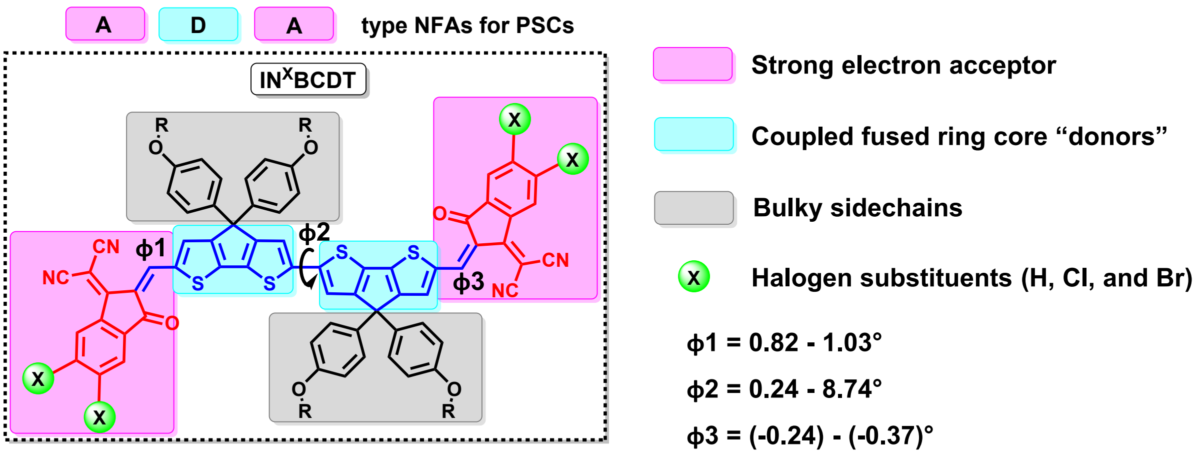

Figure S1. Chemical structure of **IN^{X}BCDT** non-fullerene acceptors (NFAs) explored as an anti-solvent additive in the fabrication of perovskite solar cells (PSCs).

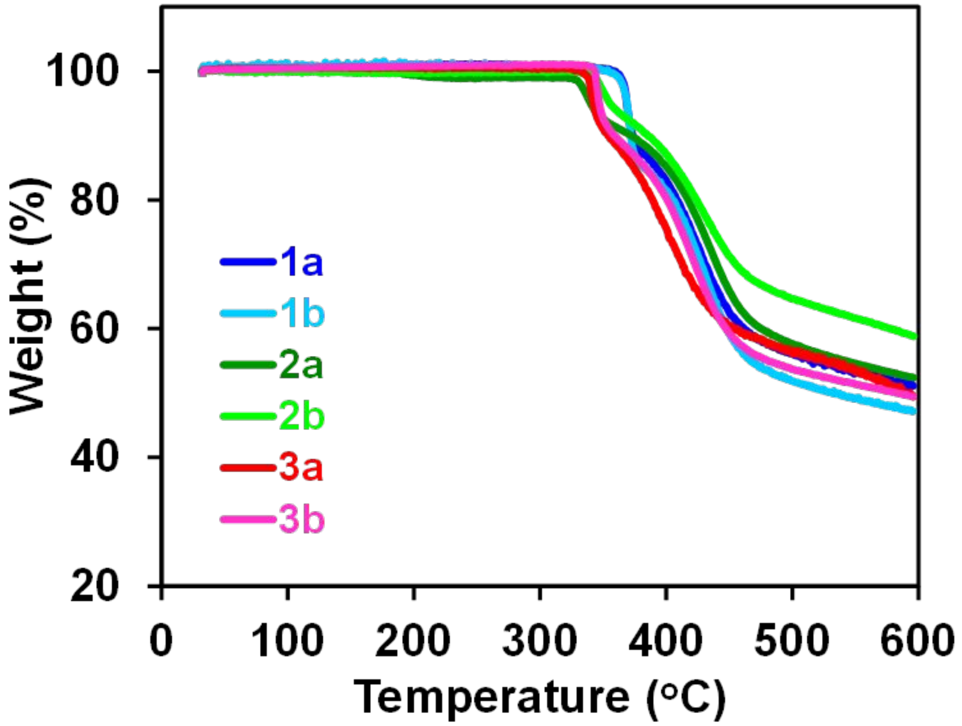

Figure S2. TGA curves of **IN^{X}BCDT** compounds (**1-3**).

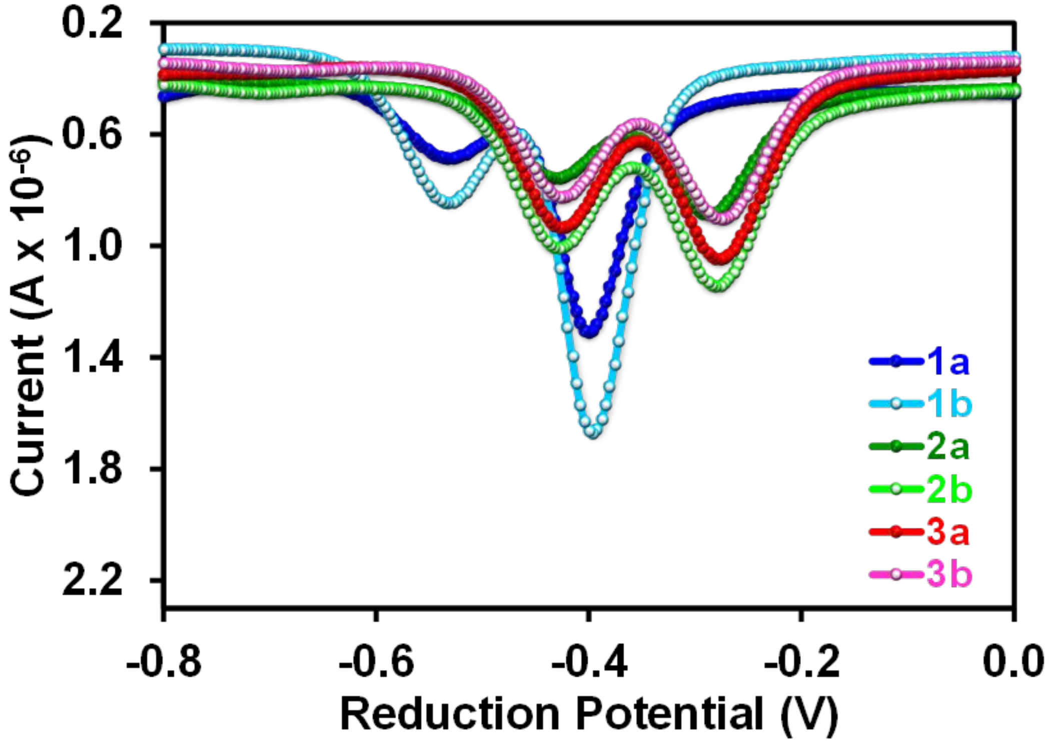

Figure S3. DPV curves of **IN^{X}BCDT** (**1-3**) in *o*-dichlorobenzene.

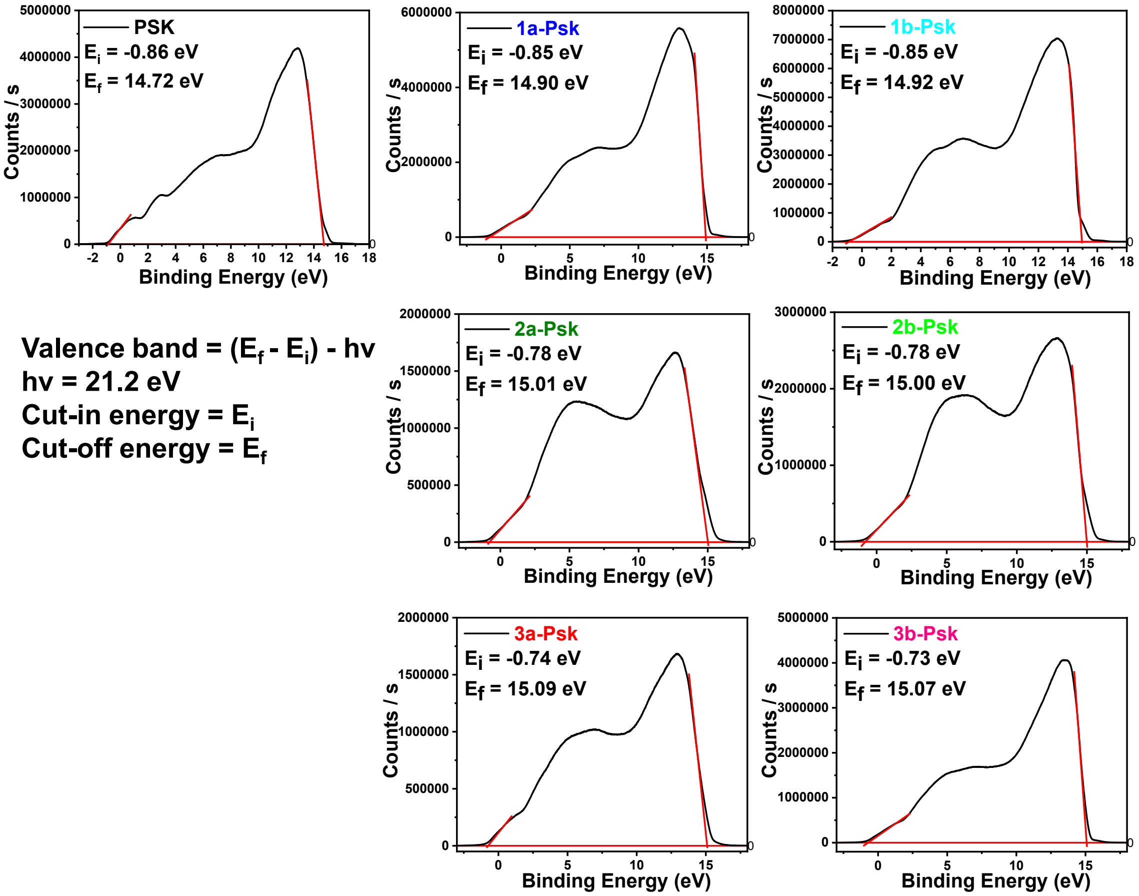

Figure S4. The UPS of the perovskite made by using pure CB (pristine perovskite), 0.2 wt% compounds **1-3** treated perovskite.

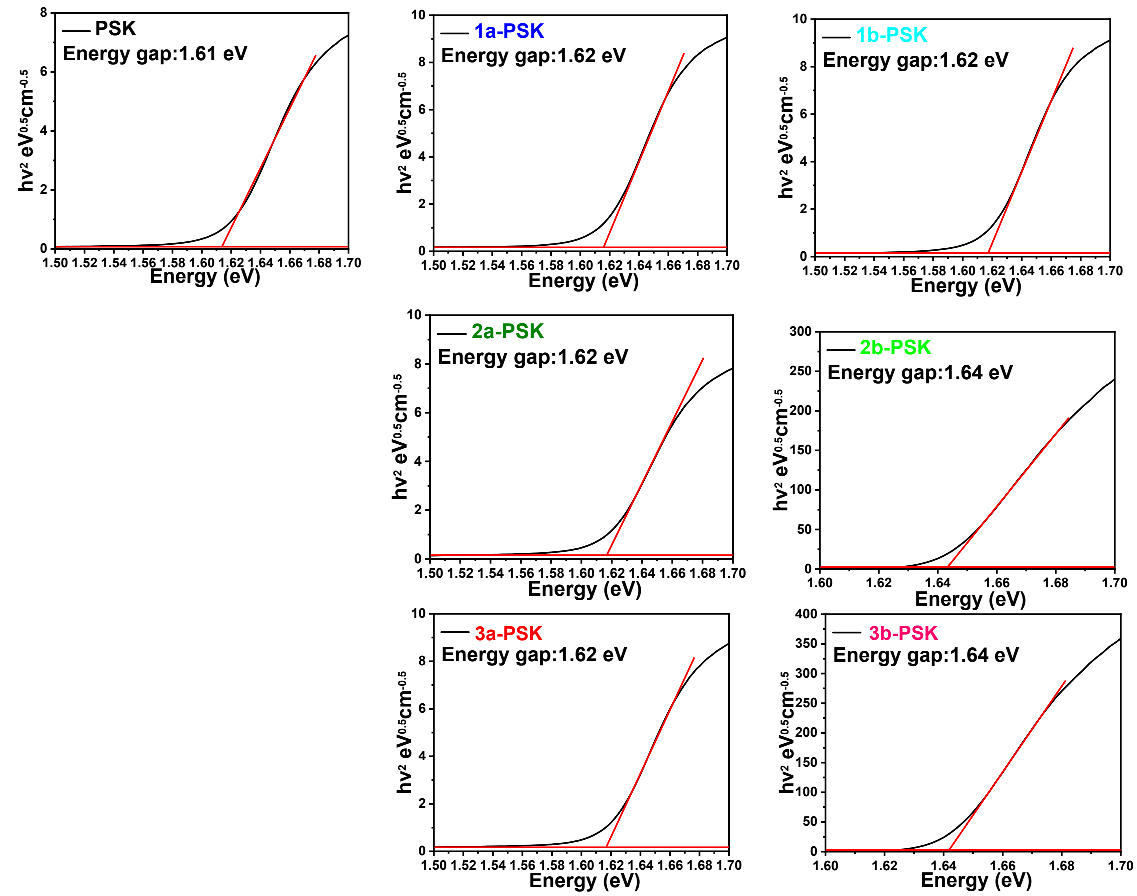

Figure S5. The optical Tauc plots of the pristine perovskite and perovskite films treated with 0.2 wt% compounds **1**-**3**.

Table S1. The energy level of the pristine perovskite and perovskite films treated with 0.2 wt% compounds **1-3**.

Film	E_f (eV)	E_i (eV)	VB (eV)
PSK	14.72	-0.86	-5.47
**1a**-PSK	14.90	-0.85	-5.45
**1b**-PSK	14.92	-0.85	-5.58
**2a**-PSK	15.01	-0.78	-5.41
**2b**-PSK	14.94	-0.78	-5.42
**3a**-PSK	15.09	-0.74	-5.37
**3b**-PSK	15.07	-0.73	-5.40

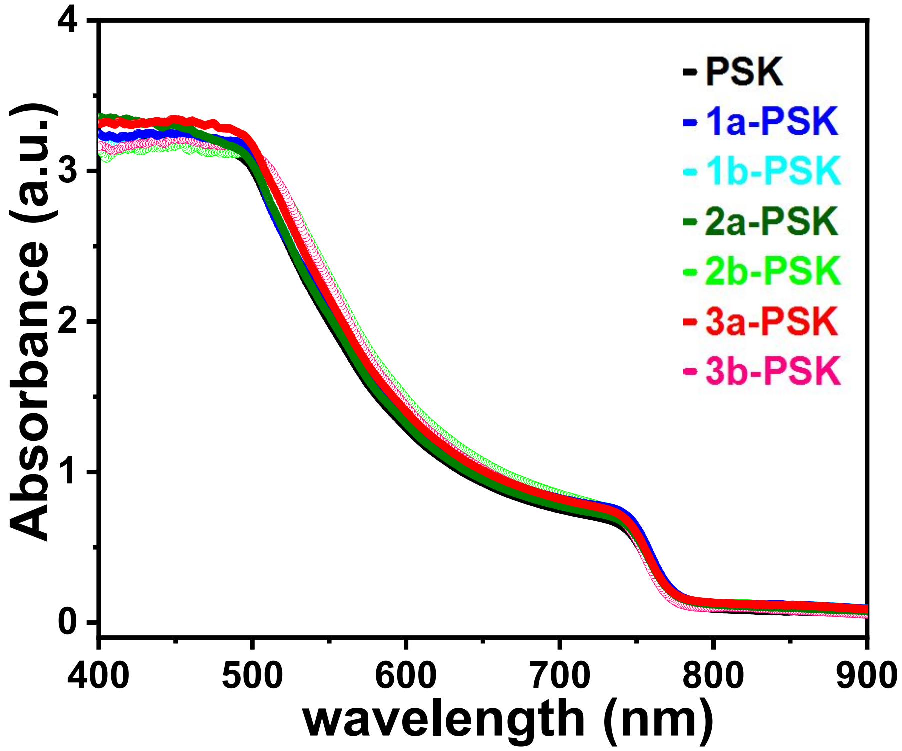

Figure S6. UV-Vis absorption spectra of pristine perovskite and perovskite films treated with 0.2 wt% compounds **1-3**.

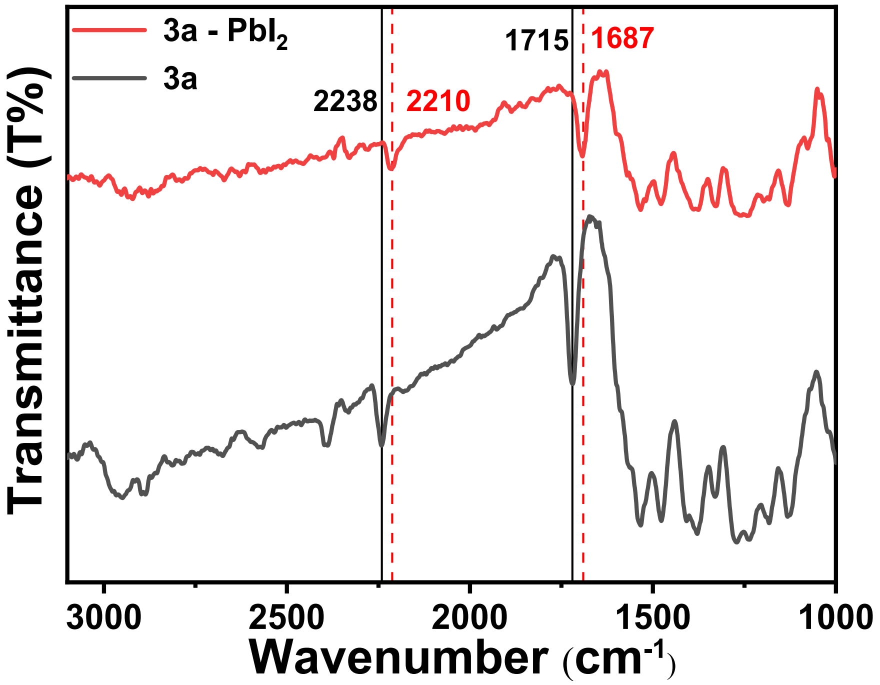

Figure S7. FTIR spectra of compound **3a** and **3a**+PbI_2.

Table S2. Steady-state PL spectra of perovskite films on glass and with Spiro-OMeTAD.

Sample	PL intensity	[a]Charge injection/transfer efficiency (η_{inj}) (%)
Glass/PSK	132	--
Glass/**1a**-PSK	251	--
Glass/**1b**-PSK	223	--
Glass/**2a**-PSK	340	--
Glass/**2b**-PSK	294	--
Glass/**3a**-PSK	375	--
Glass/**3b**-PSK	319	--
Glass/PSK/Spiro-OMeTAD	92	30.3
Glass/**1a**-PSK/Spiro-OMeTAD	47	81.3
Glass/**1b**-PSK/Spiro-OMeTAD	55	75.3
Glass/**2a**-PSK/Spiro-OMeTAD	20	94.1
Glass/**2b**-PSK/Spiro-OMeTAD	34	88.4
Glass/**3a**-PSK/Spiro-OMeTAD	6	98.4
Glass/**3b**-PSK/Spiro-OMeTAD	25	92.2

[a] Charge injection efficiency(η_{inj})= (ΔPL)/PL

Table S3. TRPL spectra of perovskite films on glass.

Films	A1	τ_1 lifetime (ns)	A2	τ_2 lifetime (ns)	τ_{av} (ns)
PSK	0.84	0.49	0.16	0.22	0.45
**1a**-PSK	0.70	0.97	0.30	6.74	2.70
**1b**-PSK	0.66	0.42	0.34	4.39	1.77
**2a**-PSK	0.73	1.37	0.27	13.21	4.57
**2b**-PSK	0.75	0.85	0.25	10.36	3.23
**3a**-PSK	0.42	1.75	0.58	11.69	7.52
**3b**-PSK	0.51	0.56	0.49	6.32	3.38

$y = A_1*\exp(-t/\tau_1) + A_2*\exp(-t/\tau_2)$

Table S4. TRPL spectra of perovskite films with Spiro-OMeTAD overlayer.

Films	A1	τ_1 lifetime (ns)	A2	τ_2 lifetime (ns)	τ_{av} (ns)
PSK/Spiro-OMeTAD	0.55	0.66	0.41	5.42	2.69
**1a**-PSK/Spiro-OMeTAD	0.81	0.27	0.19	2.31	0.66
**1b**-PSK/Spiro-OMeTAD	0.69	0.39	0.30	2.94	1.16
**2a**-PSK/Spiro-OMeTAD	0.85	0.26	0.15	2.00	0.52
**2b**-PSK/Spiro-OMeTAD	0.74	0.42	0.25	3.18	1.12
**3a**-PSK/Spiro-OMeTAD	0.80	0.24	0.20	1.29	0.45
**3b**-PSK/Spiro-OMeTAD	0.72	0.52	0.26	3.76	1.38

$y = A_1*\exp(-t/\tau_1) + A_2*\exp(-t/\tau_2)$

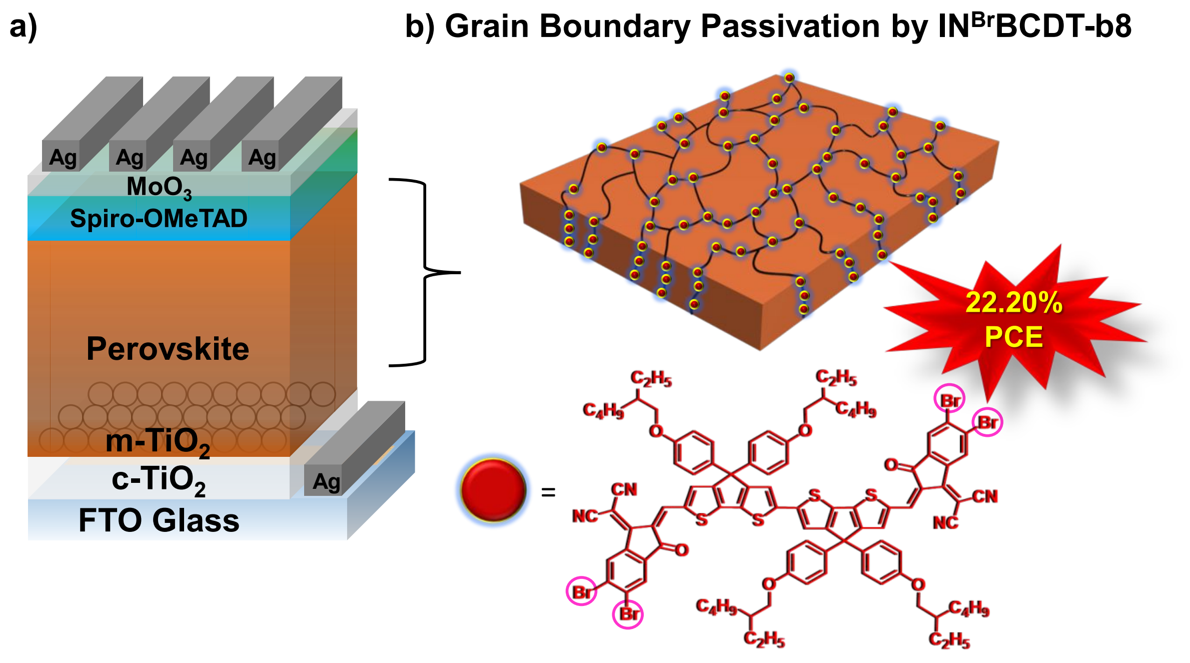

Figure S8. (a) Device architecture of PSCs and (b) Schematic diagram of grain boundary passivation by **IN^{Br}BCDT-b8**.

Table S5. The device performance parameters of PSCs based on various concentration of **IN^{Br}BCDT-b8**.

Antisolvent	J_{sc} (mA/cm^2)	V_{oc} (V)	FF	Max. PCE (%)
Chlorobenzene	24.06	1.03	73	18.09 (17.13±0.88)
0.1 wt% **IN^{Br}BCDT-b8 (3a)**$_{(CB)}$	24.17	1.11	77	20.66 (19.42±0.66)
0.2 wt% **IN^{Br}BCDT-b8 (3a)**$_{(CB)}$	24.44	1.15	79	22.20 (21.37±0.45)
0.3 wt% **IN^{Br}BCDT-b8 (3a)**$_{(CB)}$	24.55	1.14	78	21.83 (20.89±0.57)

Table S6. The *I-V* curves of the cell based on **IN^{Br}BCDT-b8** (**3a**) treated perovskite measured with both voltage scan directions.

Direction	J_{sc} (mA/cm^2)	V_{oc} (V)	FF	PCE (%)	Hysteresis index
Negative to positive bias	24.44	1.15	79	22.20	0%
Positive to negative bias	24.46	1.15	79	22.20	

Hysteresis index = $(PCE_{reverse} - PCE_{forward})/PCE_{reverse}$

Table S7. The *I-V* curves of the cell based on **pristine** perovskite measured with both voltage scan directions.

Direction	J_{sc} (mA/cm^2)	V_{oc} (V)	FF	PCE (%)	Hysteresis index
Negative to positive bias	24.06	1.05	73	18.09	37%
Positive to negative bias	23.29	0.83	59	11.41	

Hysteresis index = $(PCE_{reverse} - PCE_{forward})/PCE_{reverse}$

Table S8. The integrated photocurrent density from IPCE and short-circuit current density from *J-V* of the reference cell and cells based on compounds **1**-**3**-treated perovskite films.

Film	Device Photocurrent Density (Jsc) (mA/cm^2)	Integrated Photocurrent Density from IPCE (mA/cm^2)
Pristine PSK	24.06	21.92
**1a**-PSK	24.71	22.55
**1b**-PSK	24.57	22.44
**2a**-PSK	24.41	22.19
**2b**-PSK	24.21	22.01
**3a**-PSK	24.43	22.34
**3b**-PSK	24.17	21.82

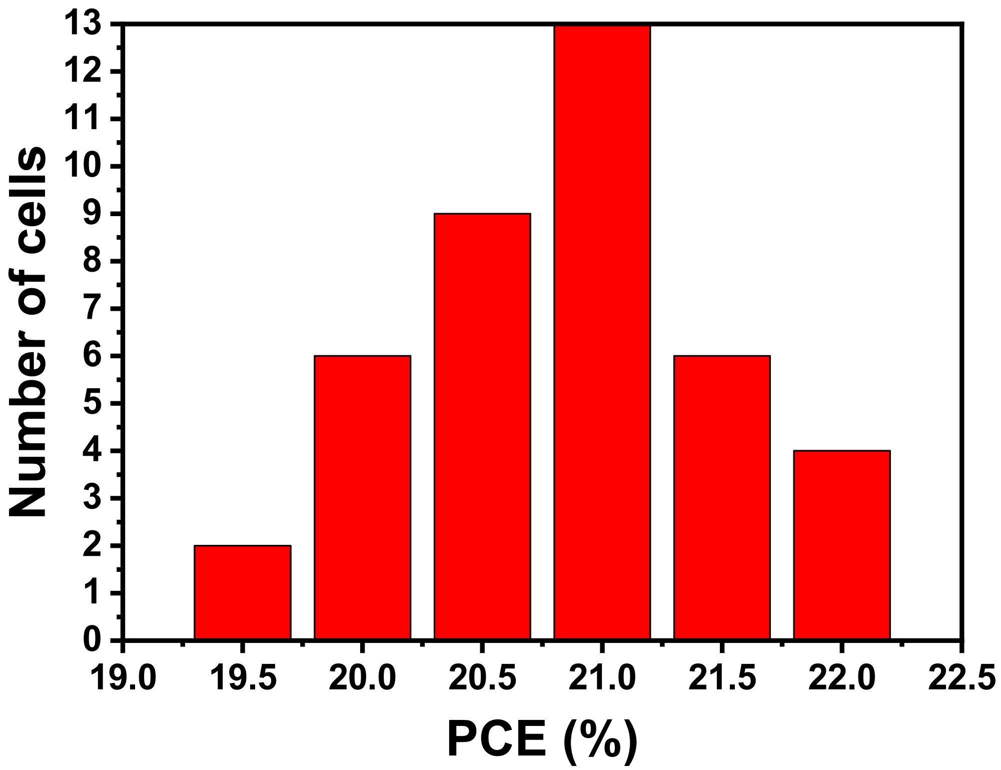

Figure S9. The efficiency distribution for perovskite solar cells based on **IN^{Br}BCDT-b8** (**3a**) treated perovskite absorbers (40 devices, 21.37 ± 0.45%).

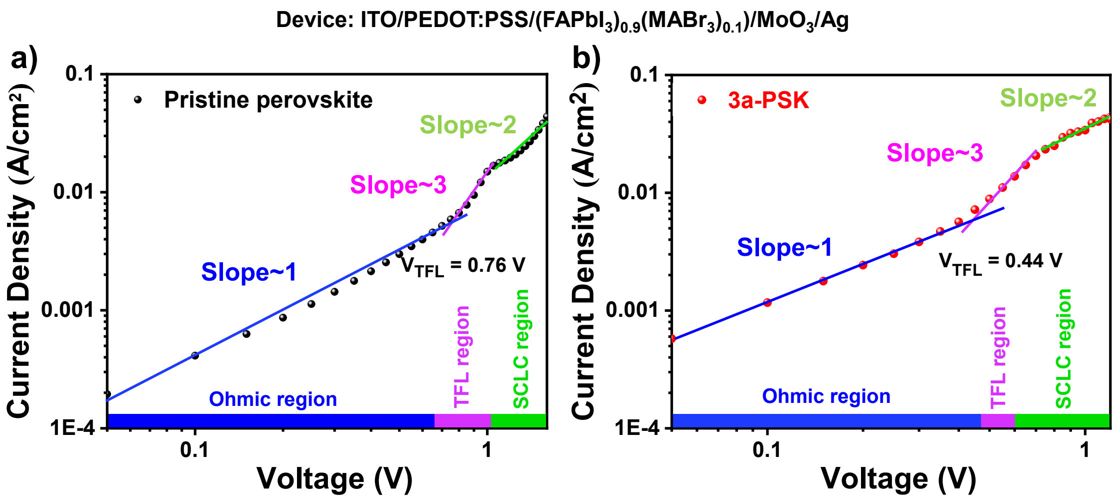

Figure S10. Hole-only device and current-voltage measurement curves of the (a) Pristine perovskite and (b) **IN^{Br}BCDT-b8-PSK** (**3a**-PSK) films.

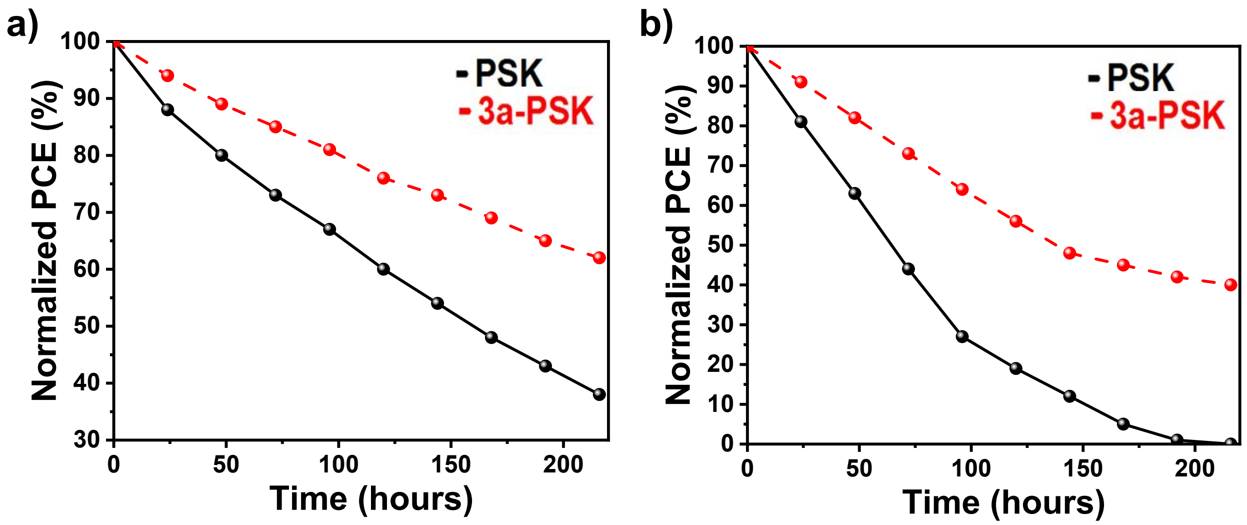

Figure S11. The PCE decay of the PSCs based on PSK and **3a**-PSK absorbers a) By storing at 85 ˚C in a glove box without encapsulation, b) By storing at 50% relative humidity in air with encapsulation.

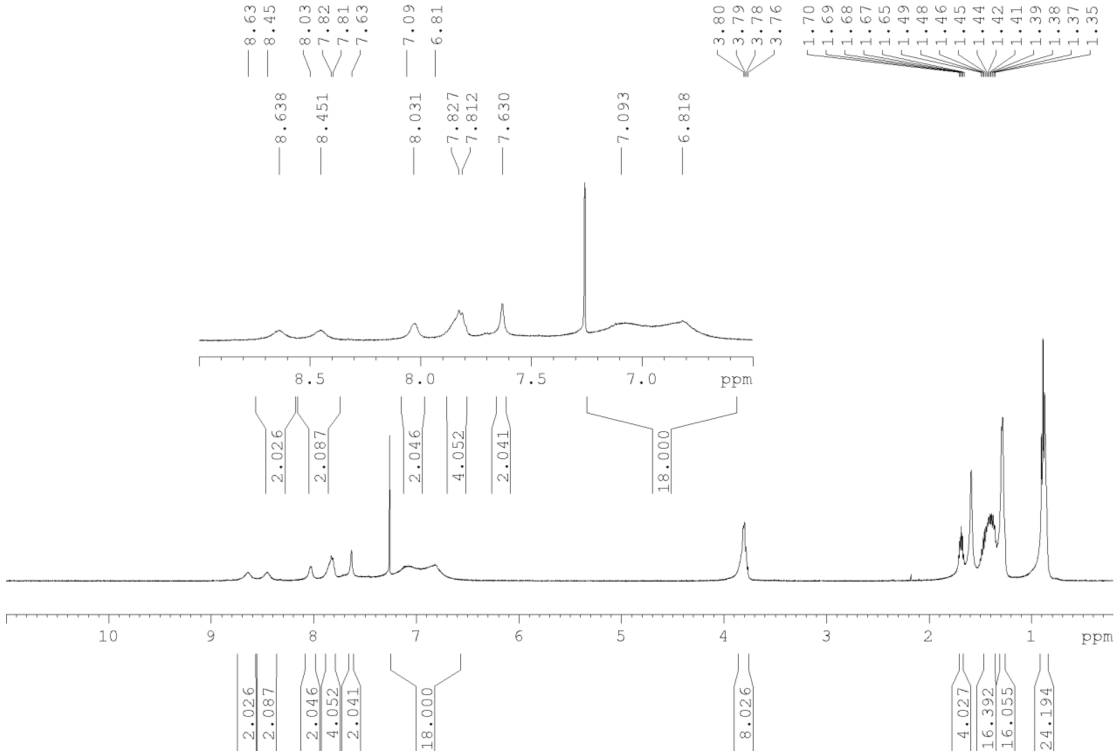

Figure S12. ^{1}H NMR spectrum of **INBCDT-b8 (1a)** in $CDCl_3$

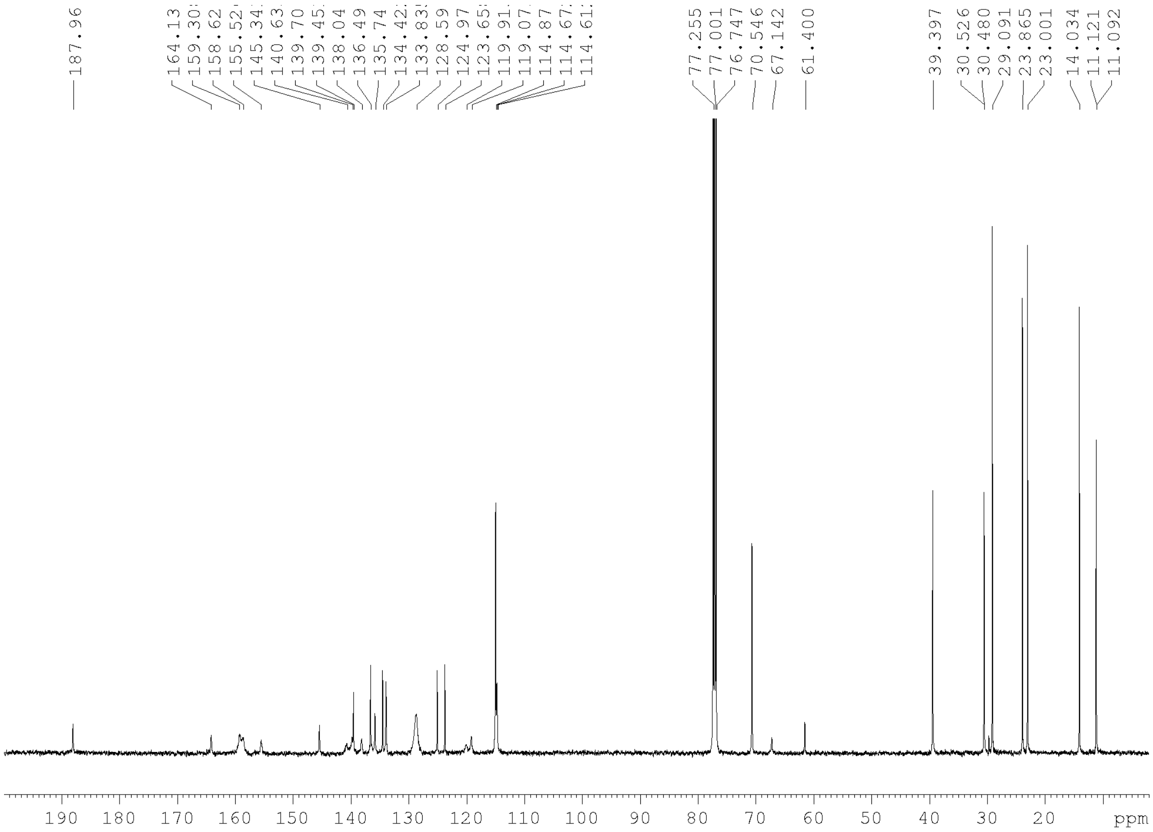

Figure S13. ^{13}C NMR spectrum of **INBCDT-b8 (1a)** in $CDCl_3$

Formula	Mass	Error	mSigma	DblEq	N rule	Electron Configuration
C 100 H 98 N 4 O 6 S 4	1,578.6364	0.9960	68.7393	54.00	ok	odd

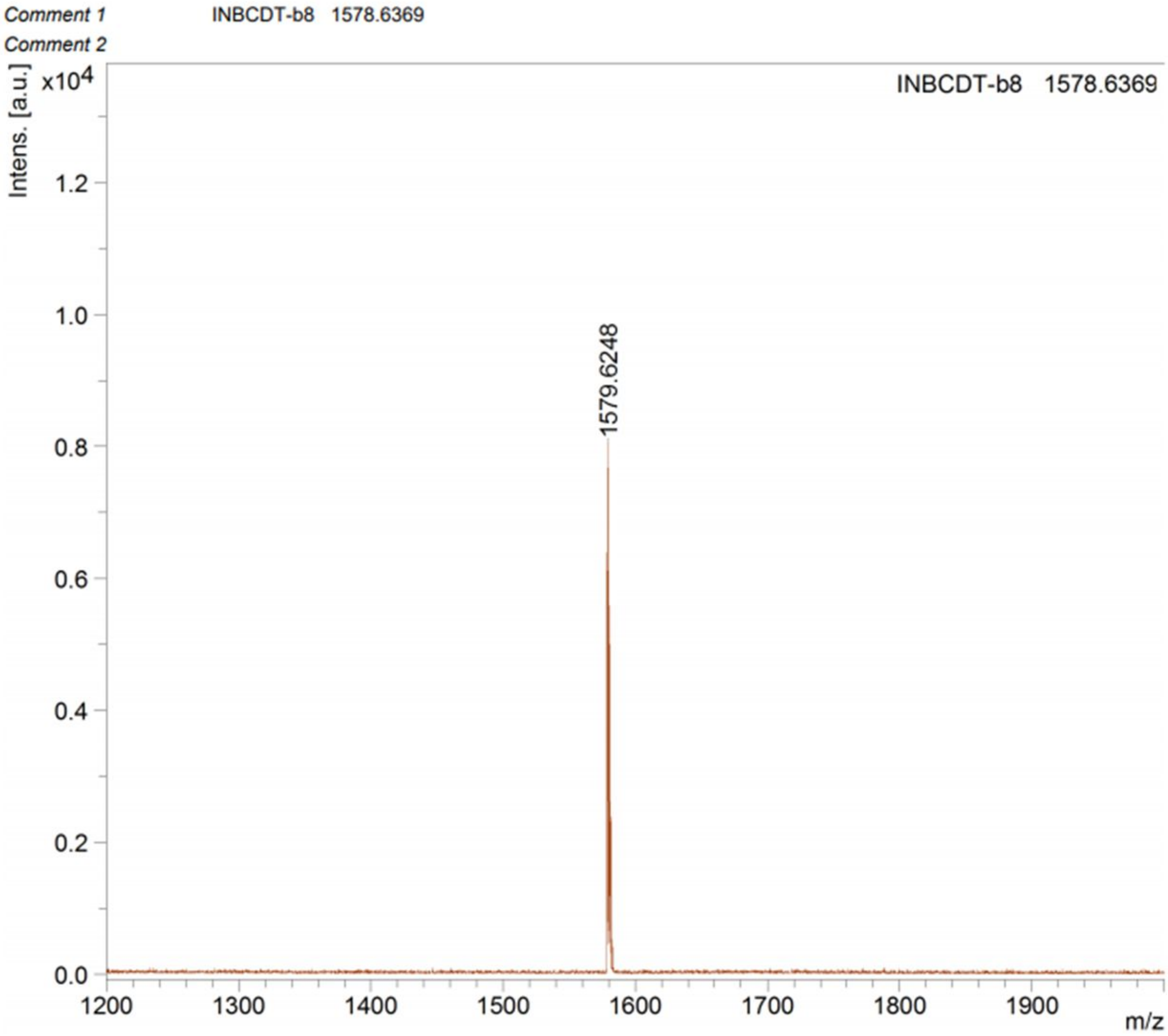

Figure S14. HRMS spectrum of **INBCDT-b8 (1a)**

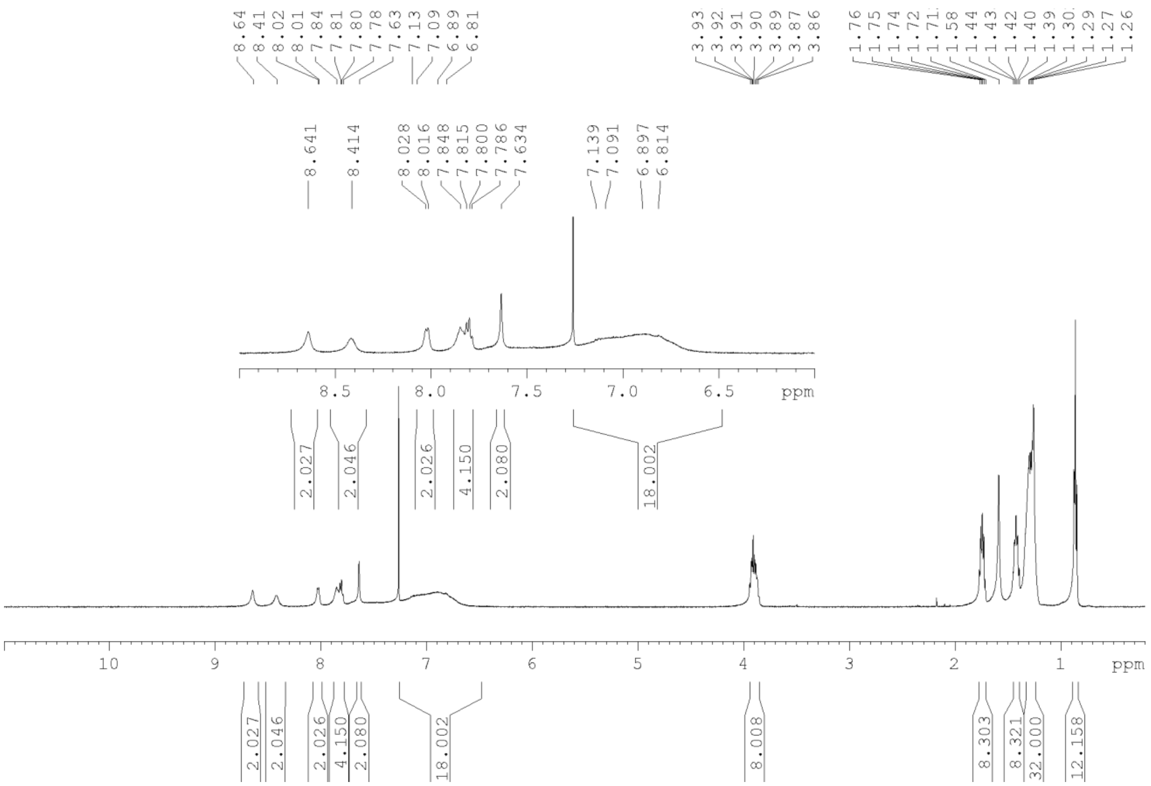

Figure S15. ^{1}H NMR spectrum of **INBCDT-8 (1b)** in $CDCl_3$

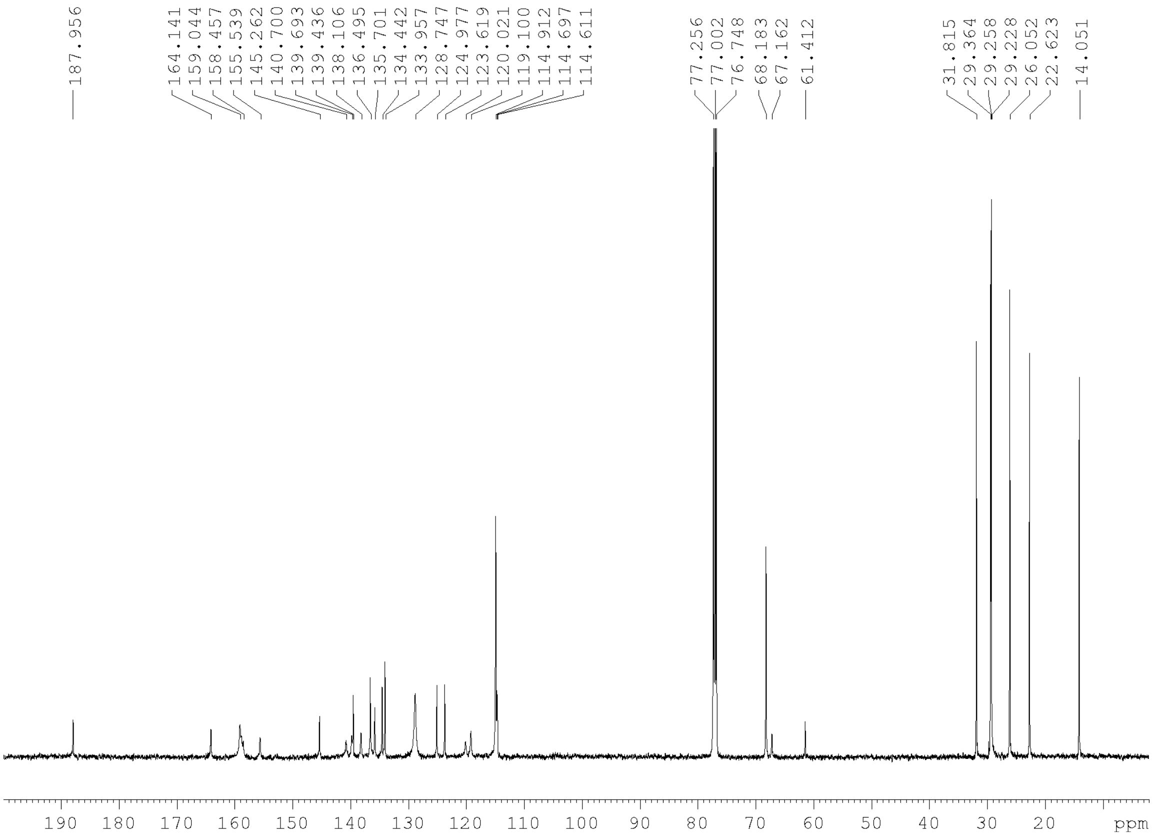

Figure S16. ^{13}C NMR spectrum of **INBCDT-8 (1b)** in $CDCl_3$

Formula	Mass	Error	mSigma	DblEq	N rule	Electron Configuration
C 100 H 98 N 4 O 6 S 4	1,578.6364	2.0729	109.2949	54.00	ok	odd

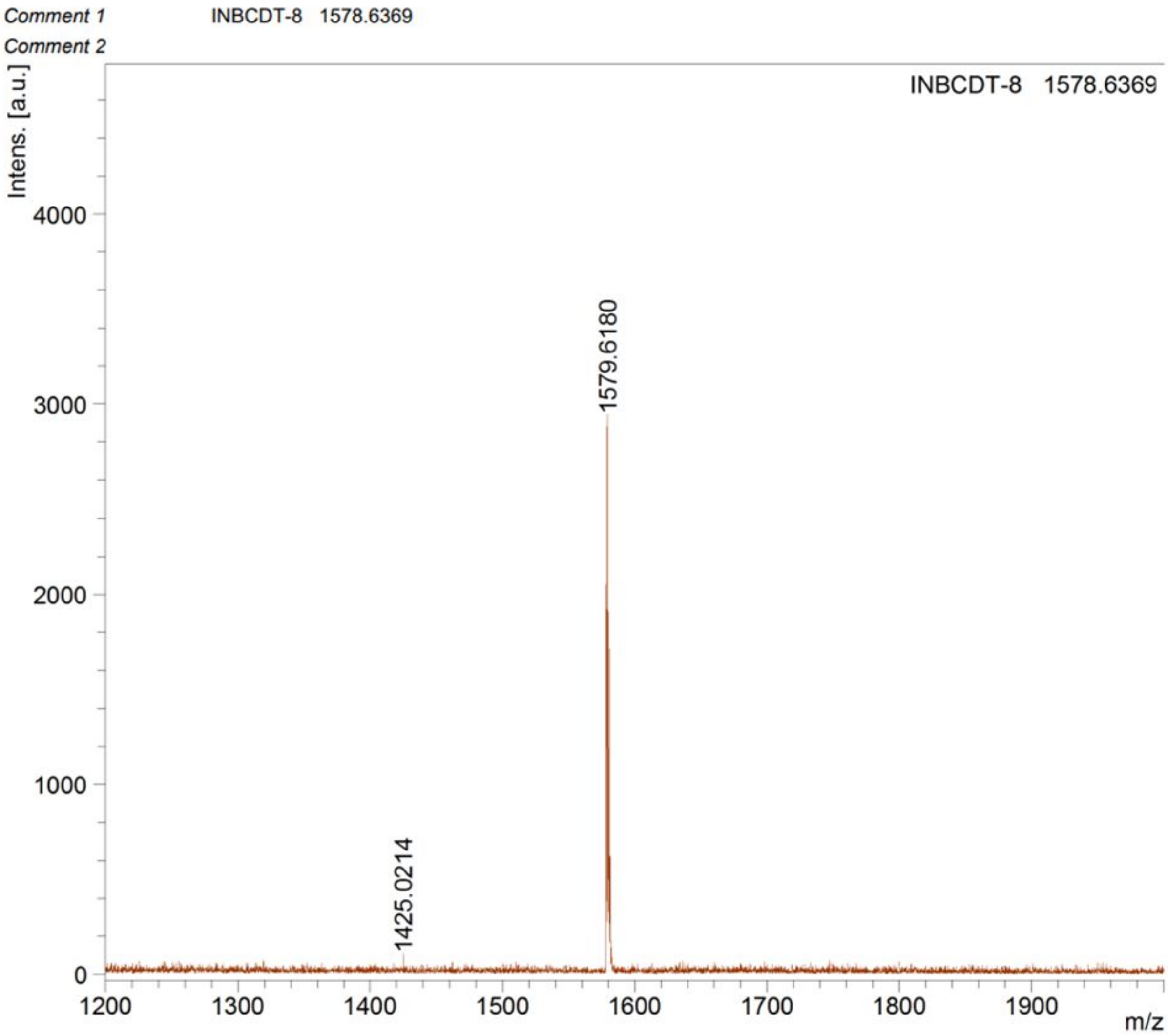

Figure S17. HRMS spectrum of **INBCDT-8 (1b)**

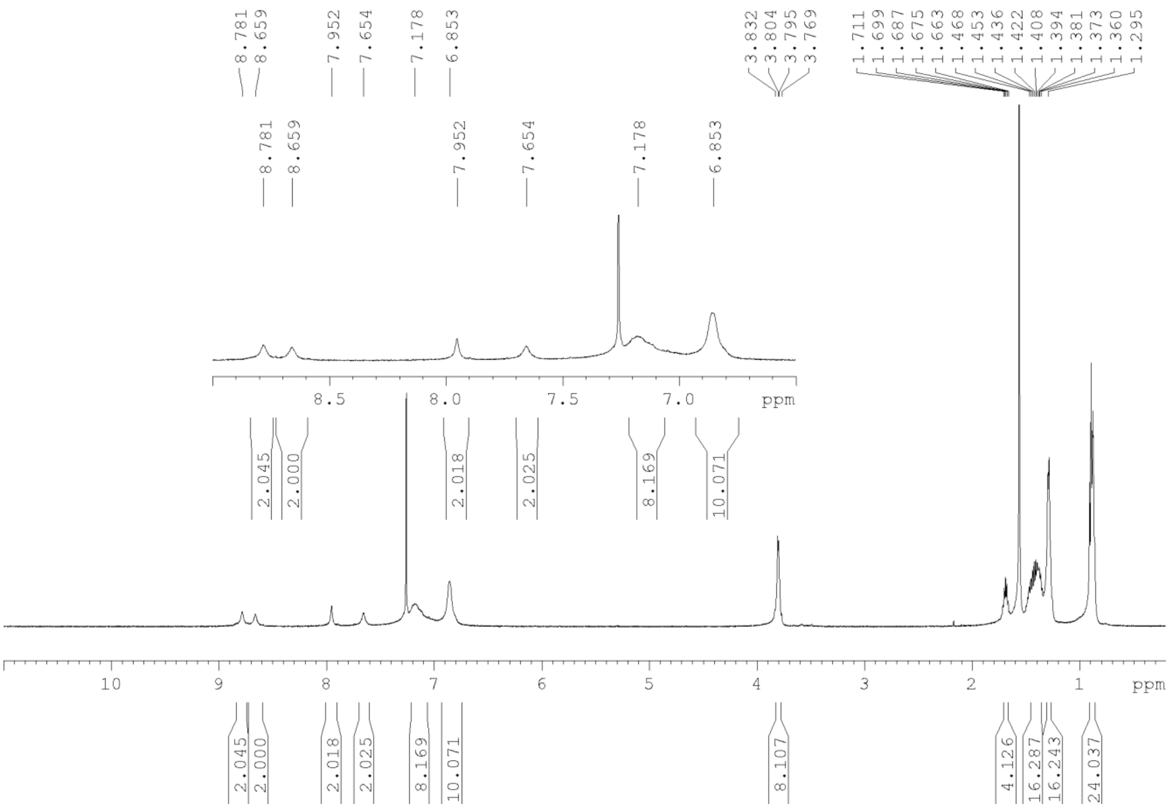

Figure S18. ^{1}H NMR spectrum of **IN^{Cl}BCDT-b8 (2a)** in $CDCl_3$

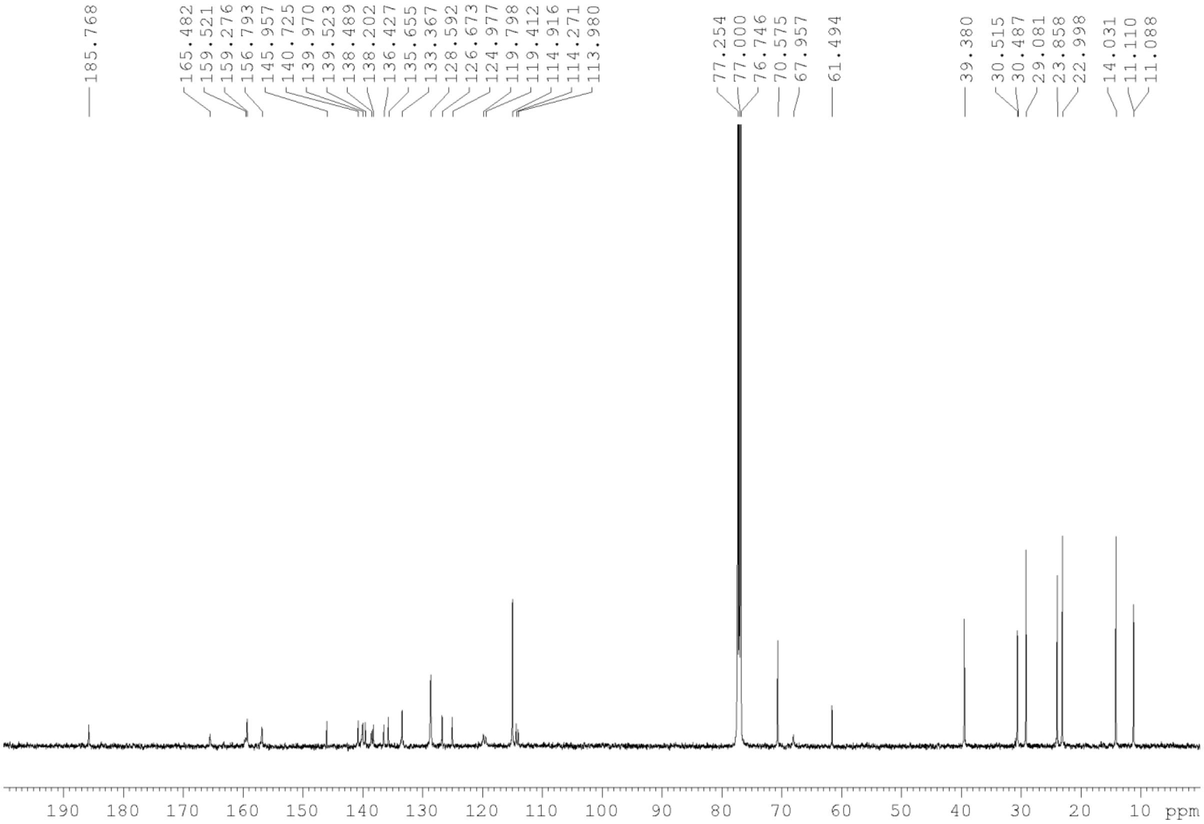

Figure S19. ^{13}C NMR spectrum of **IN^{Cl}BCDT-b8 (2a)** in $CDCl_3$

Formula	Mass	Error	mSigma	DblEq	N rule	Electron Configuration
C 100 H 94 Cl 4 N 4 O 6 S 4	1,714.4805	2.8480	225.2037	54.00	ok	odd

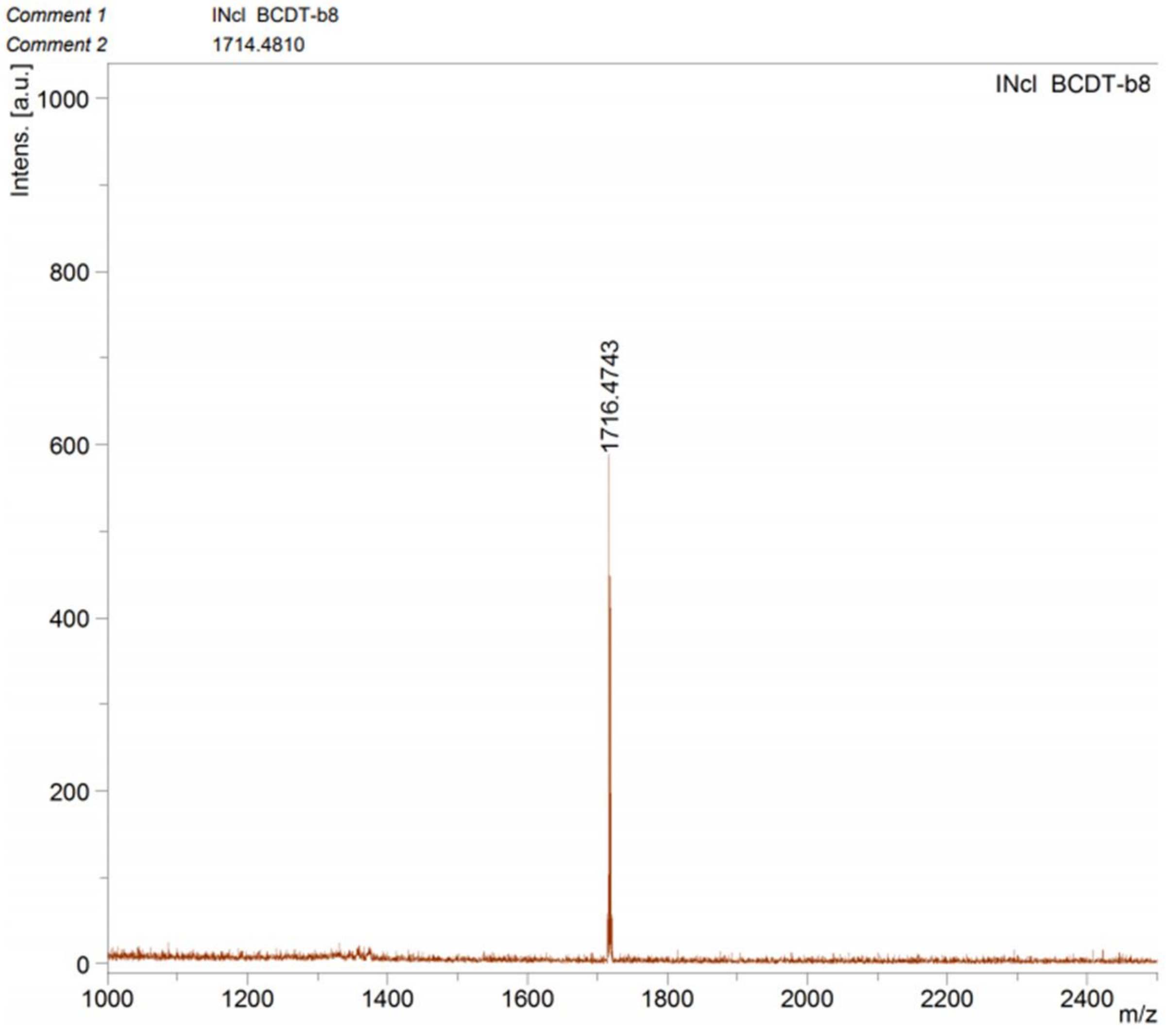

Figure S20. HRMS spectrum of **IN^{Cl}BCDT-b8 (2a)**

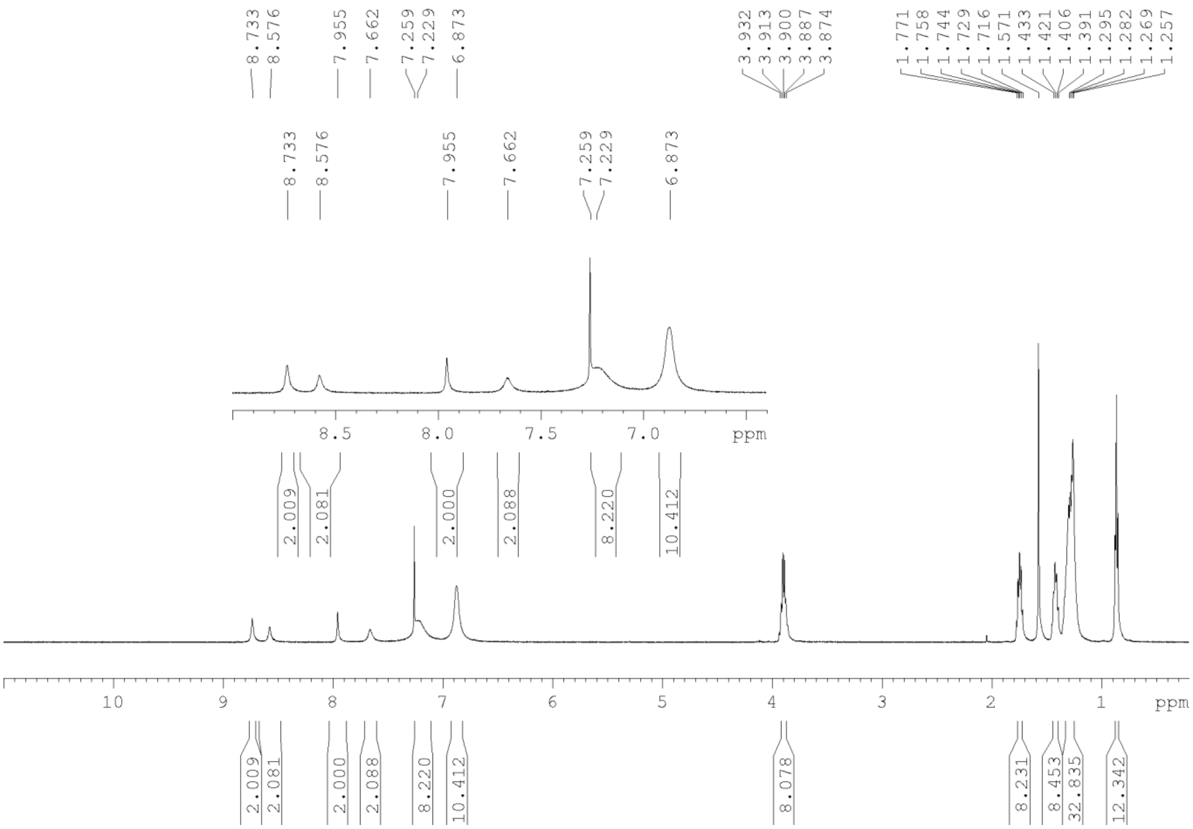

Figure S21. ^{1}H NMR spectrum of **IN^{Cl}BCDT-8 (2b)** in $CDCl_3$

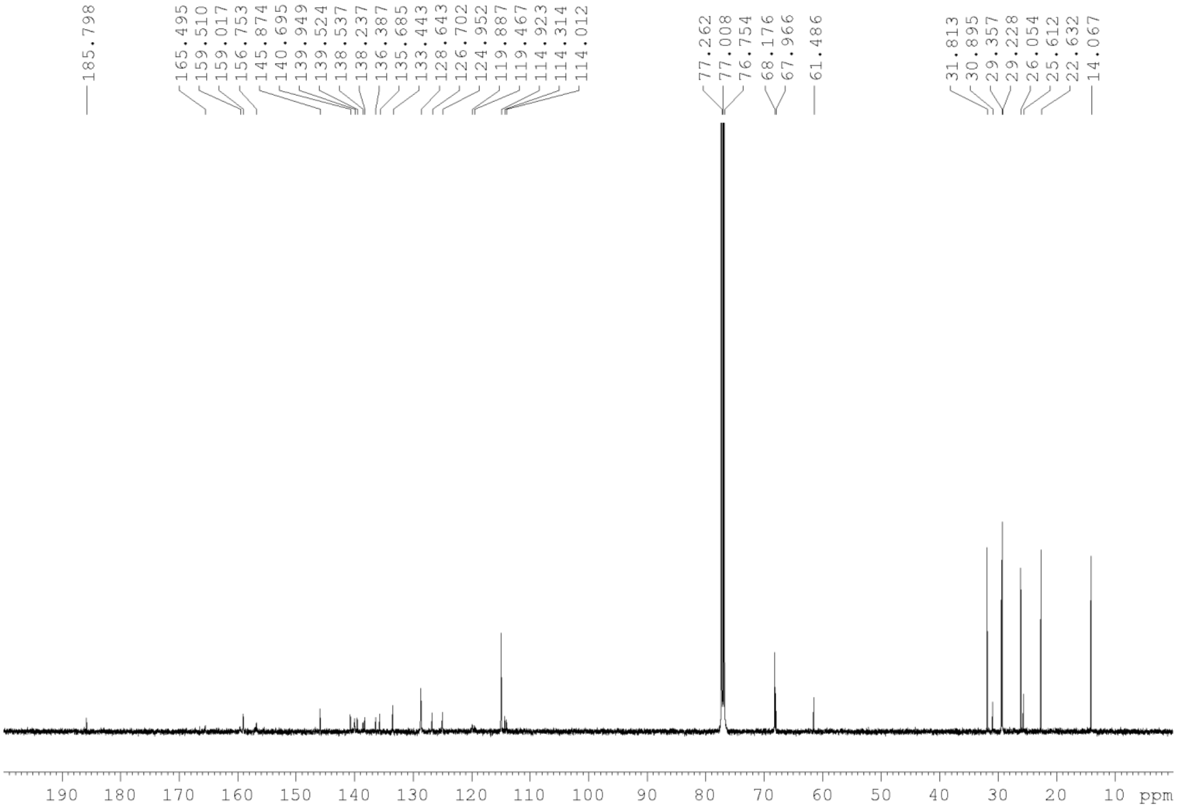

Figure S22. ^{13}C NMR spectrum of **IN^{Cl}BCDT-8 (2b)** in $CDCl_3$

Formula	Mass	Error	mSigma	DblEq	N rule	Electron Configuration
C 100 H 94 Cl 4 N 4 O 6 S 4	1,714.4805	2.9334	200.7649	54.00	ok	odd

Comment 1 INcl BCDT-8

Comment 2 1714.4810

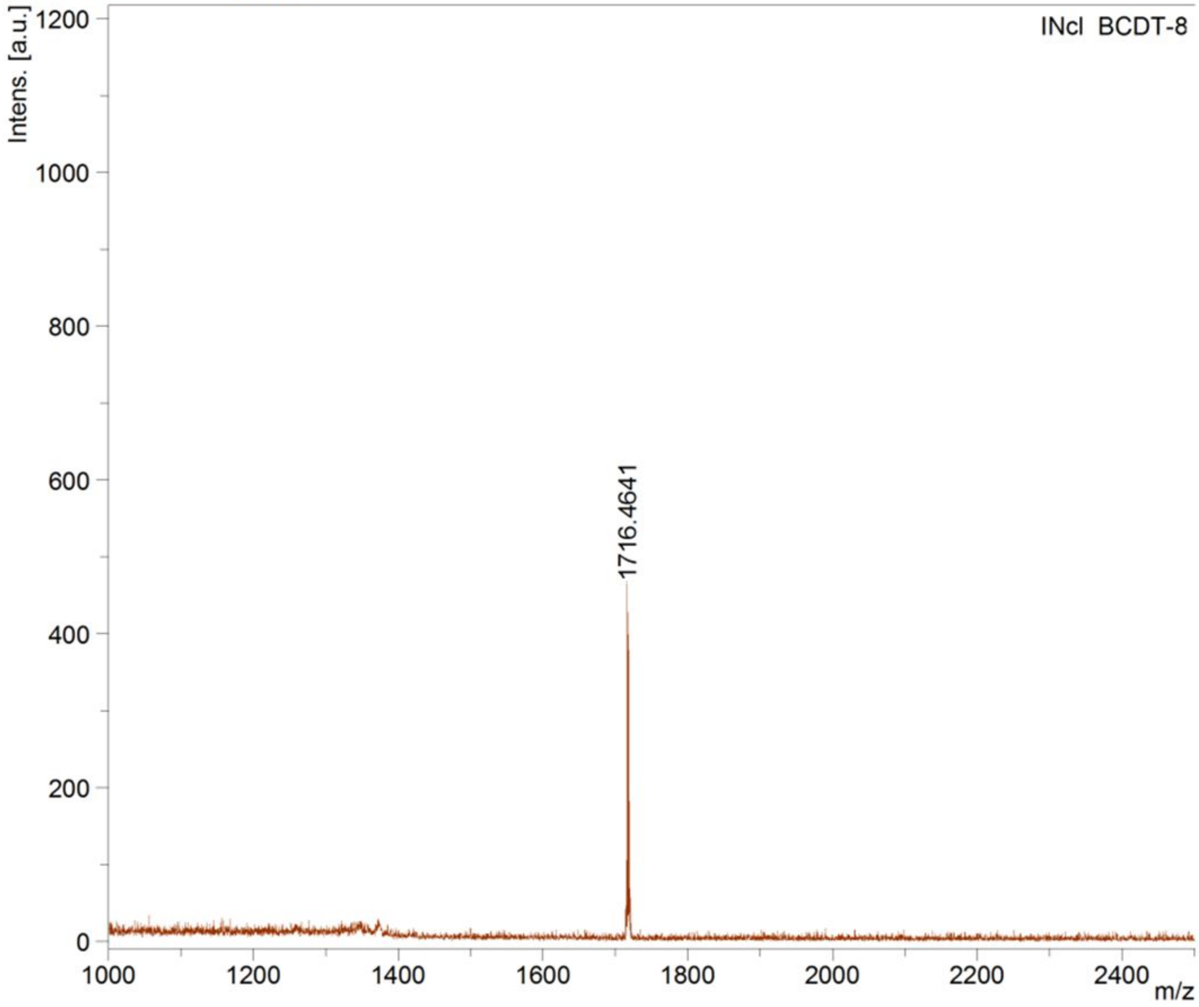

Figure S23. HRMS spectrum of **IN^{Cl}BCDT-8 (2b)**

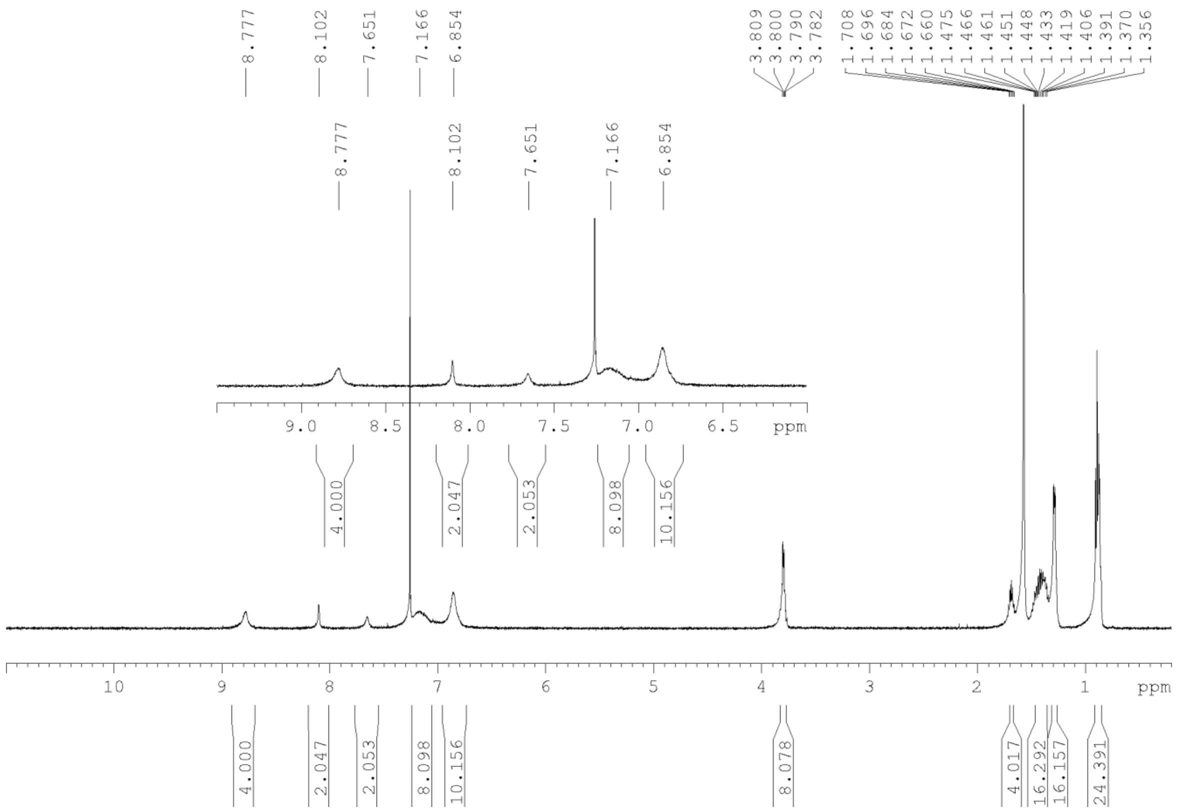

Figure S24. ^{1}H NMR spectrum of **IN^{Br}BCDT-b8 (3a)** in $CDCl_3$

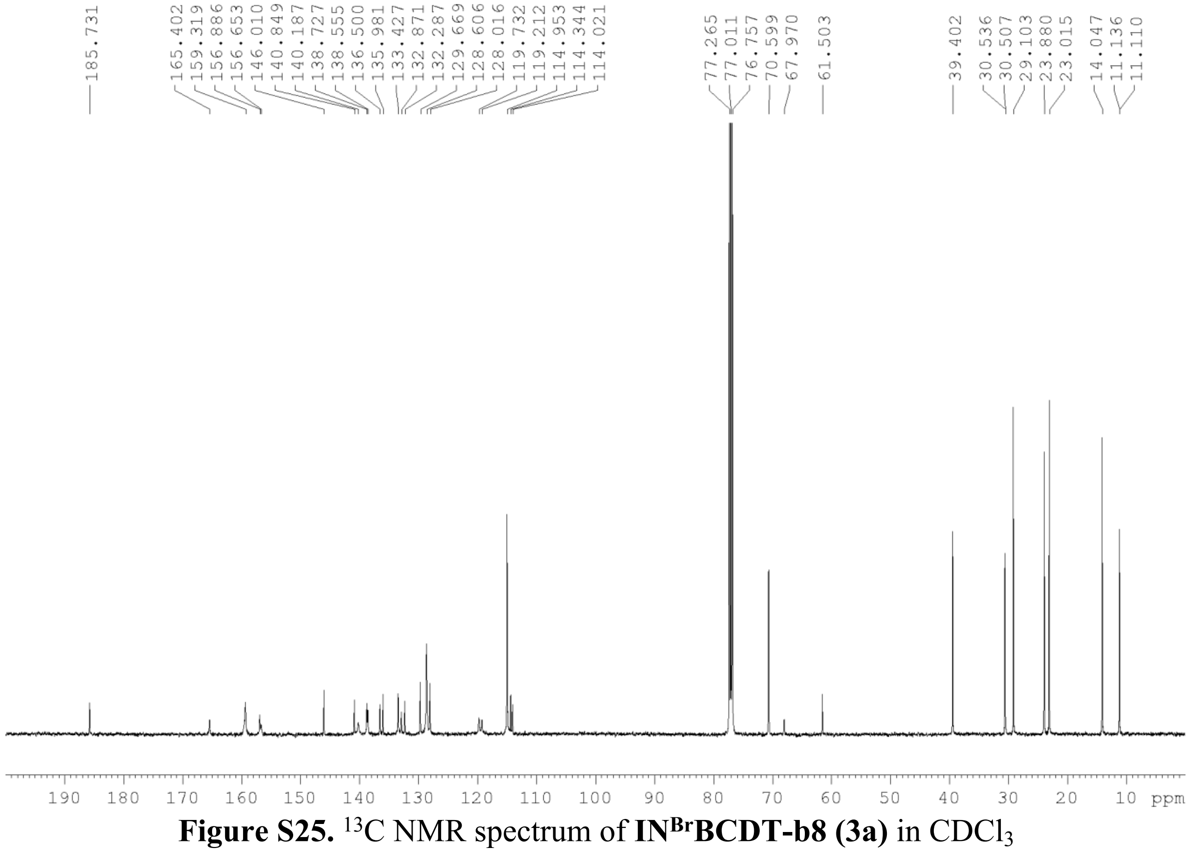

Figure S25. ^{13}C NMR spectrum of **IN^{Br}BCDT-b8 (3a)** in $CDCl_3$

Formula	Mass	Error	mSigma	DblEq	N rule	Electron Configuration
C 100 H 94 Br 4 N 4 O 6 S 4	1,890.2784	0.5748	98.4736	54.00	ok	odd

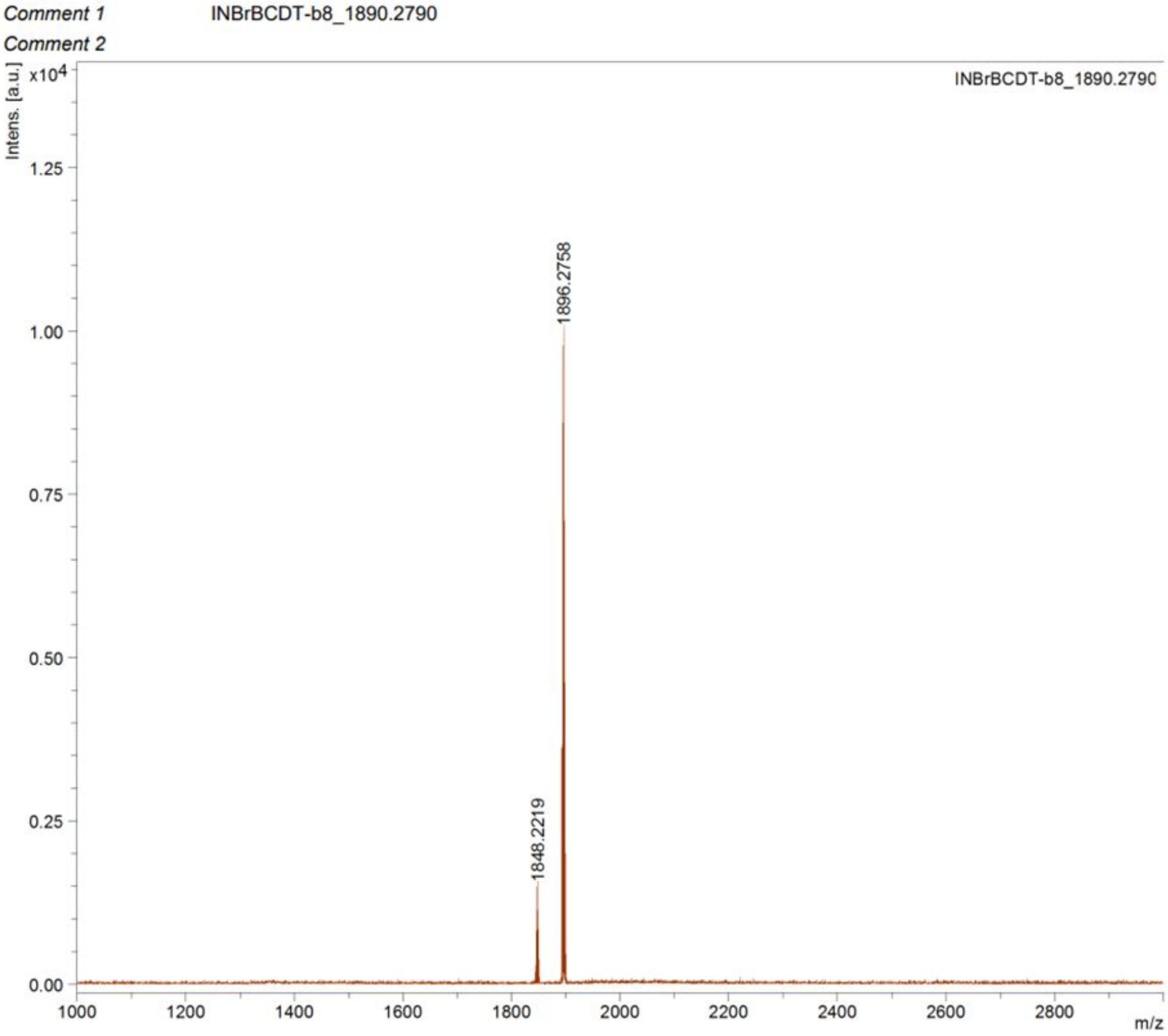

Figure S26. HRMS spectrum of **IN^{Br}BCDT-b8 (3a)**

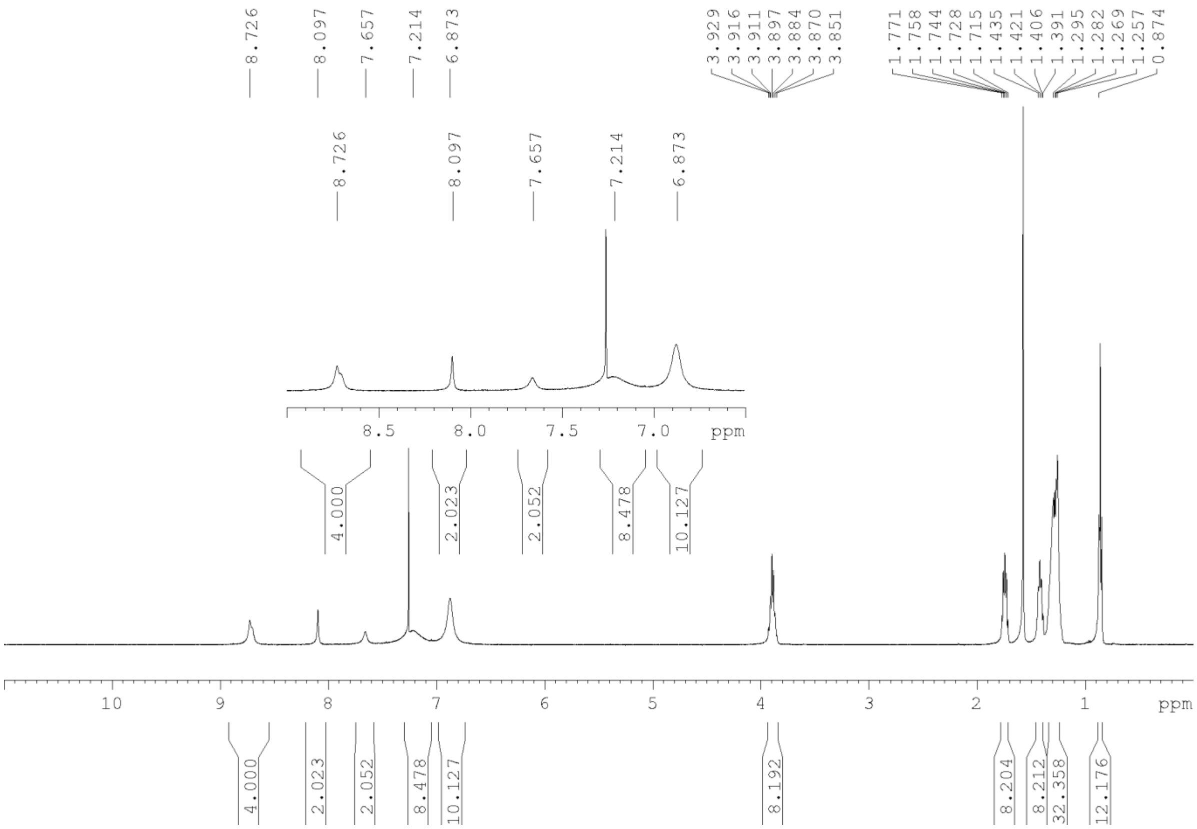

Figure S27. ^{1}H NMR spectrum of **IN^{Br}BCDT-8 (3b)** in $CDCl_3$

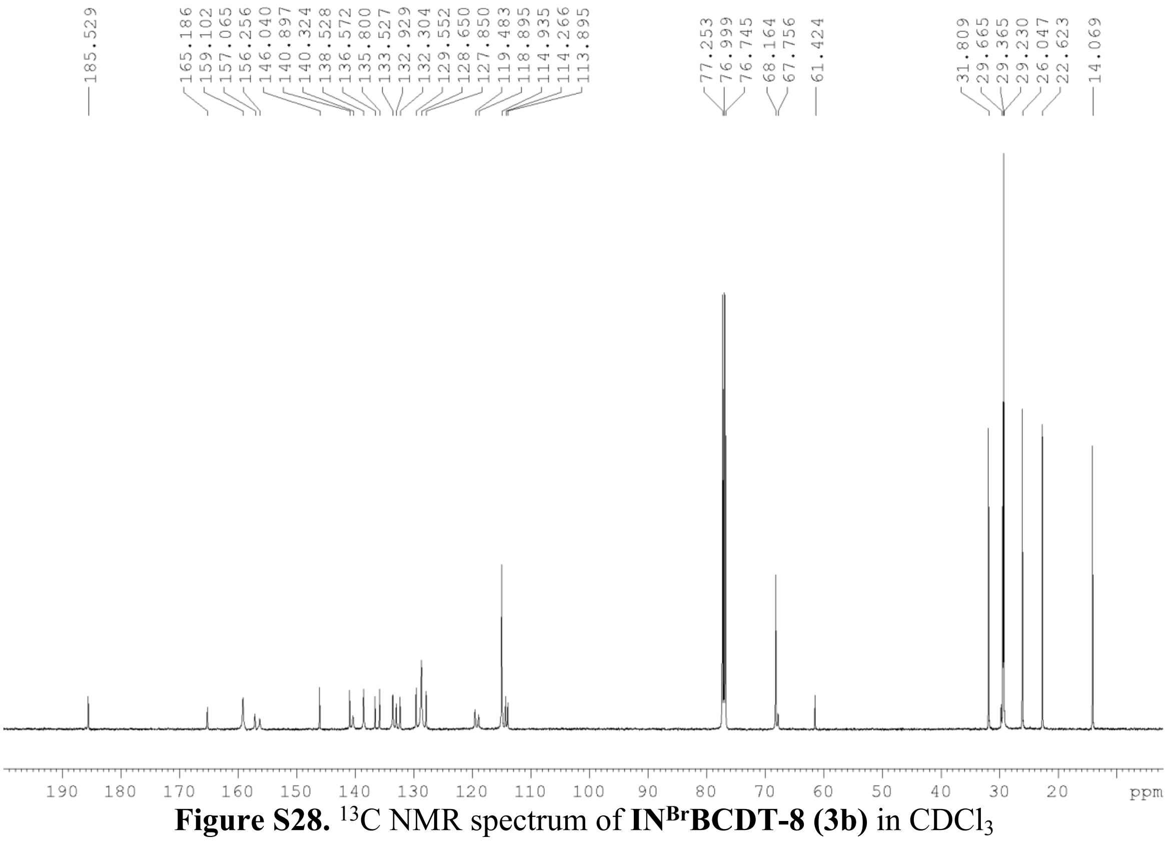

Figure S28. ^{13}C NMR spectrum of **IN^{Br}BCDT-8 (3b)** in $CDCl_3$

Formula	Mass	Error	mSigma	DblEq	N rule	Electron Configuration
C 100 H 94 Br 4 N 4 O 6 S 4	1,890.2784	0.1031	73.2324	54.00	ok	odd

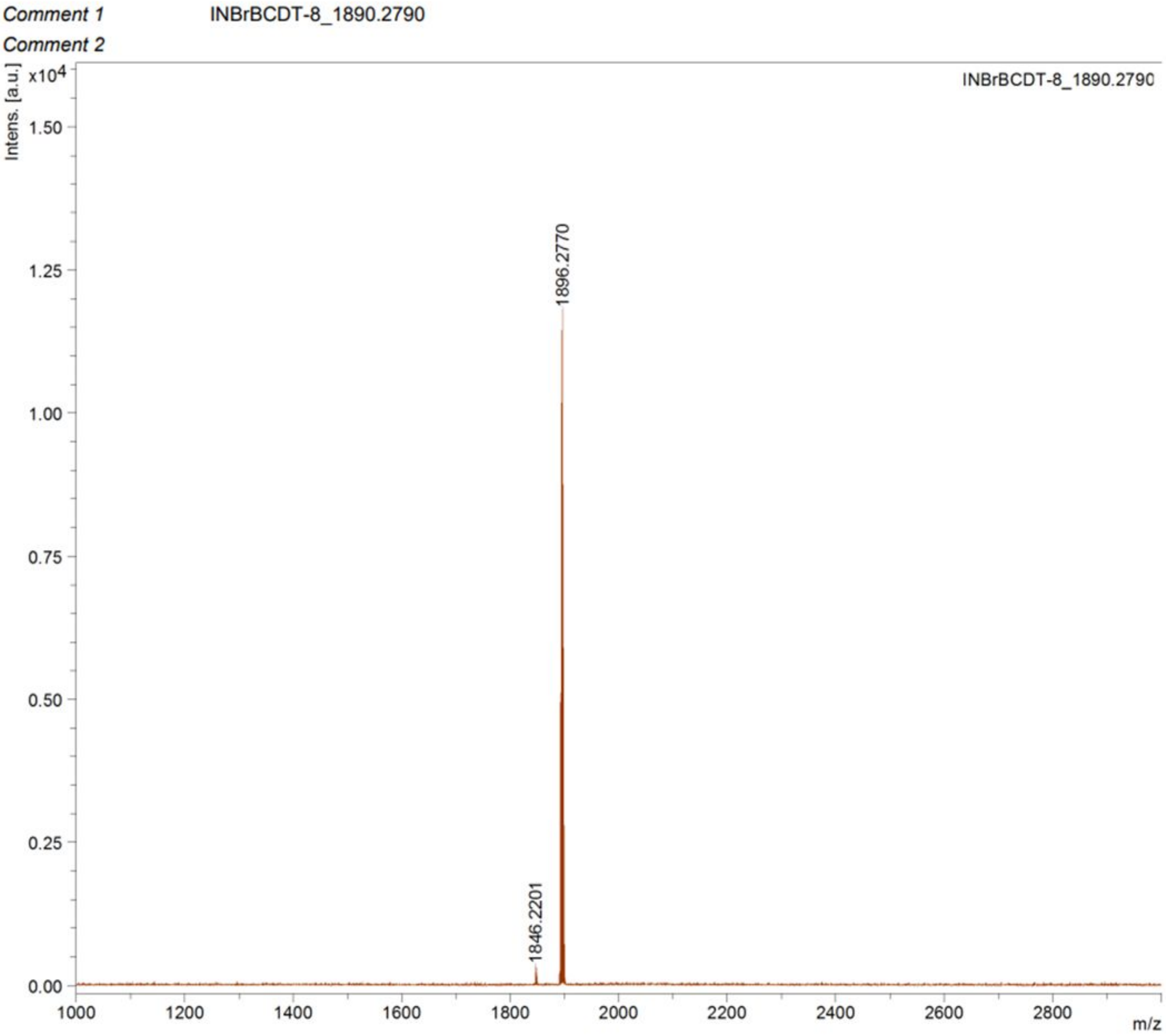

Figure S29. HRMS spectrum of **IN^{Br}BCDT-8 (3b)**

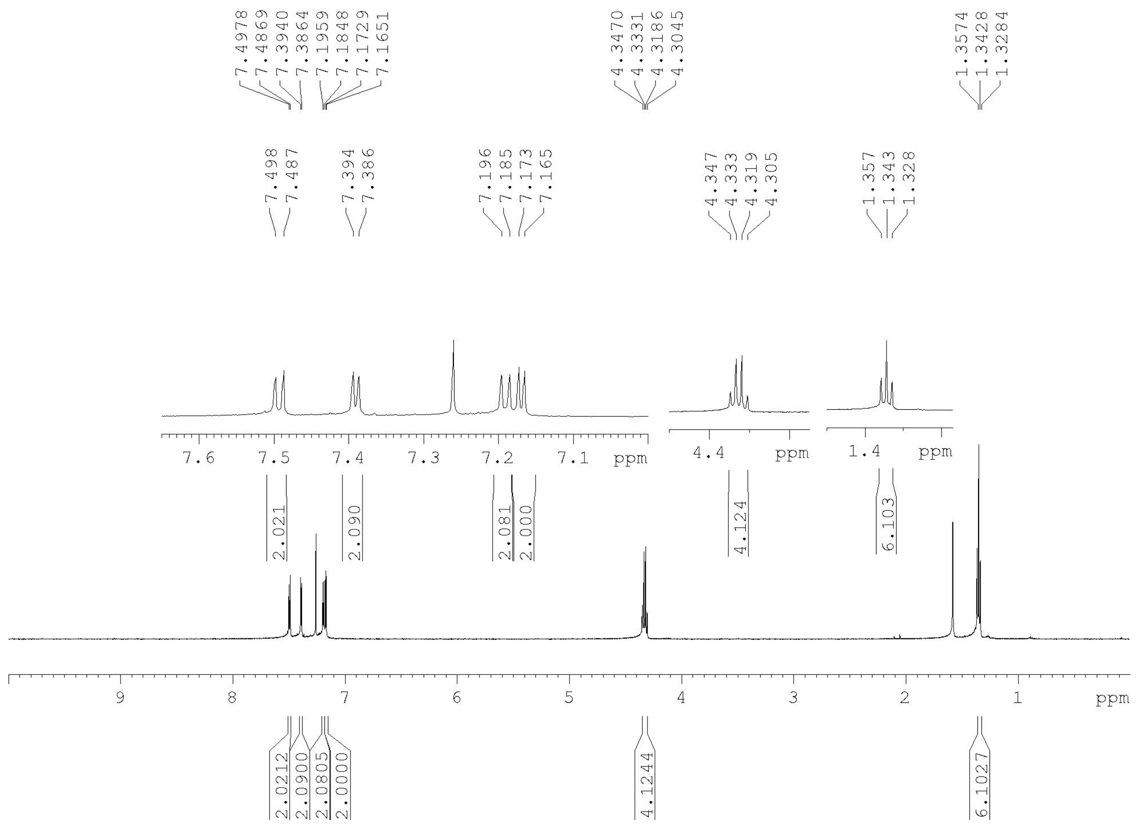

Figure S30. ^{1}H NMR spectrum of compound **6** in $CDCl_3$

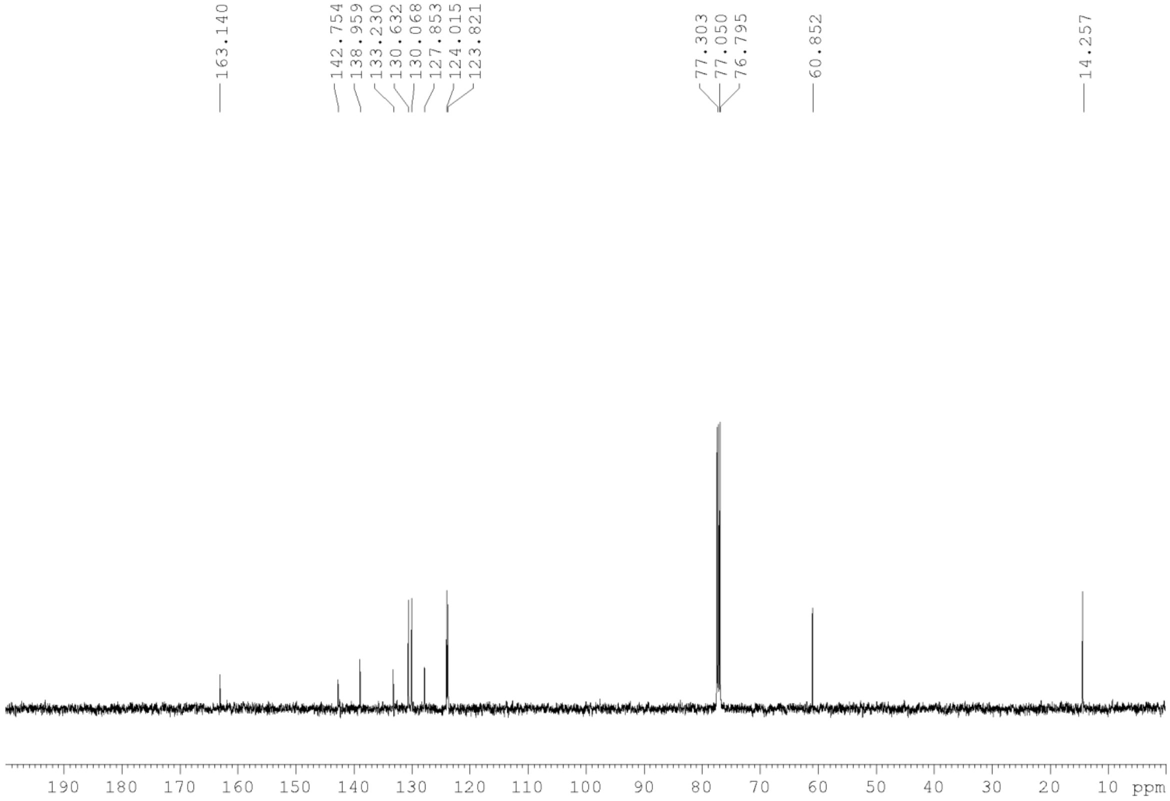

Figure S31. ^{13}C NMR spectrum of compound **6** in $CDCl_3$

Formula	Mass	Error	mSigma	DblEq	N rule	Electron Configuration
C 22 H 18 O 4 S 4	474.0082	0.9140	37.7801	14.00	ok	odd

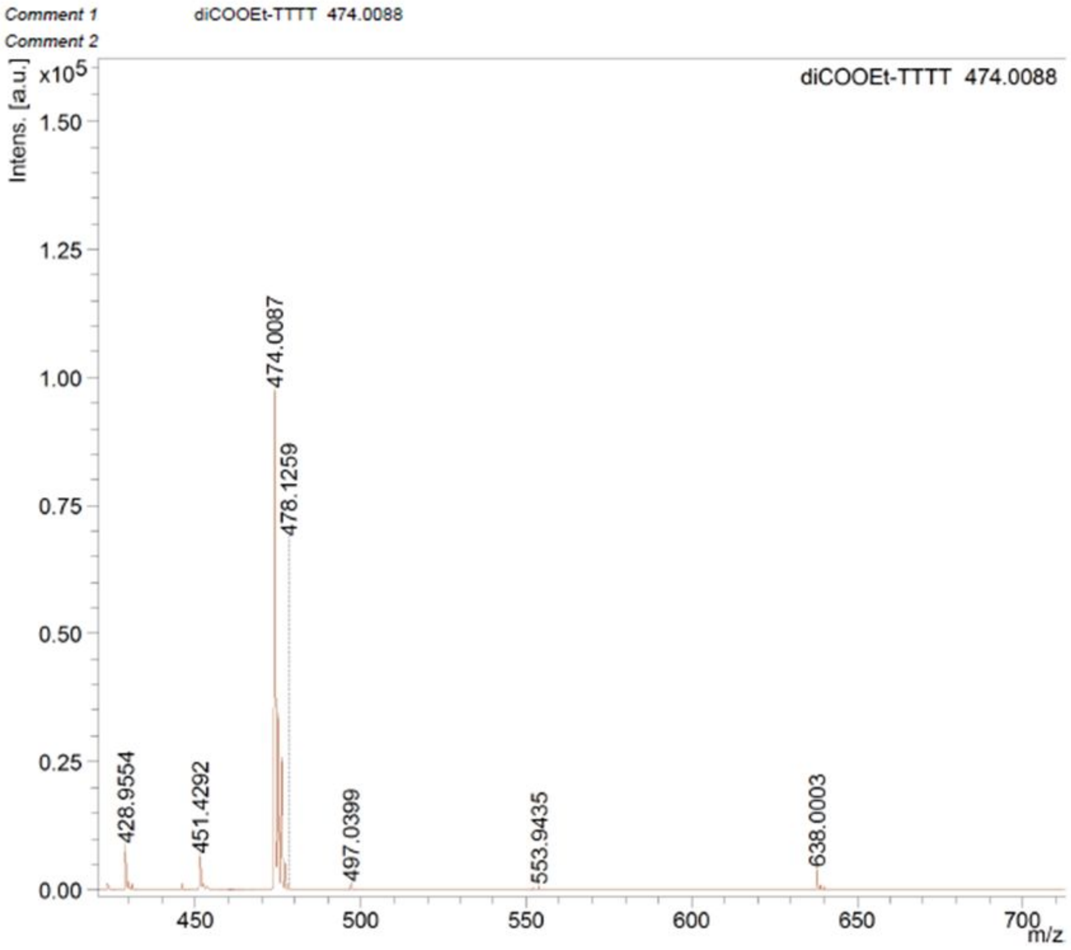

Figure S32. HRMS spectrum of compound **6**

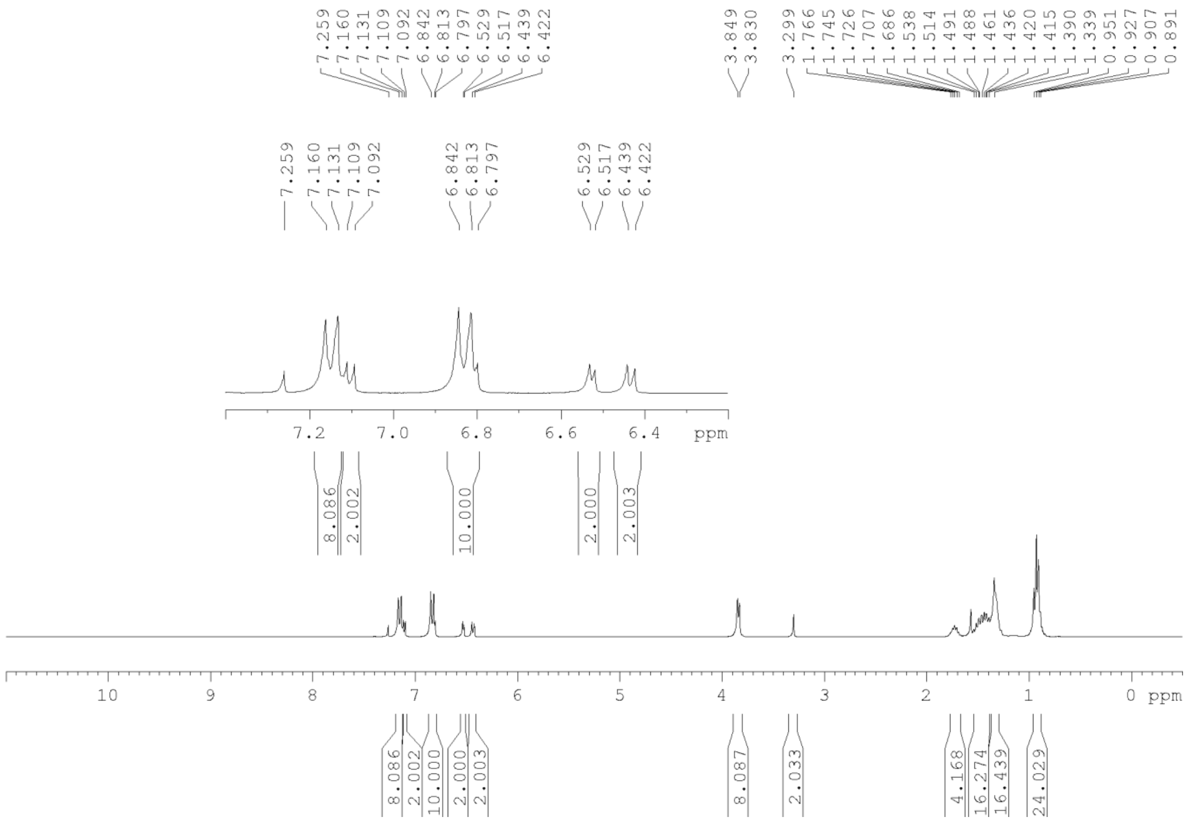

Figure S33. ^{1}H NMR spectrum of compound **8a** in $CDCl_3$

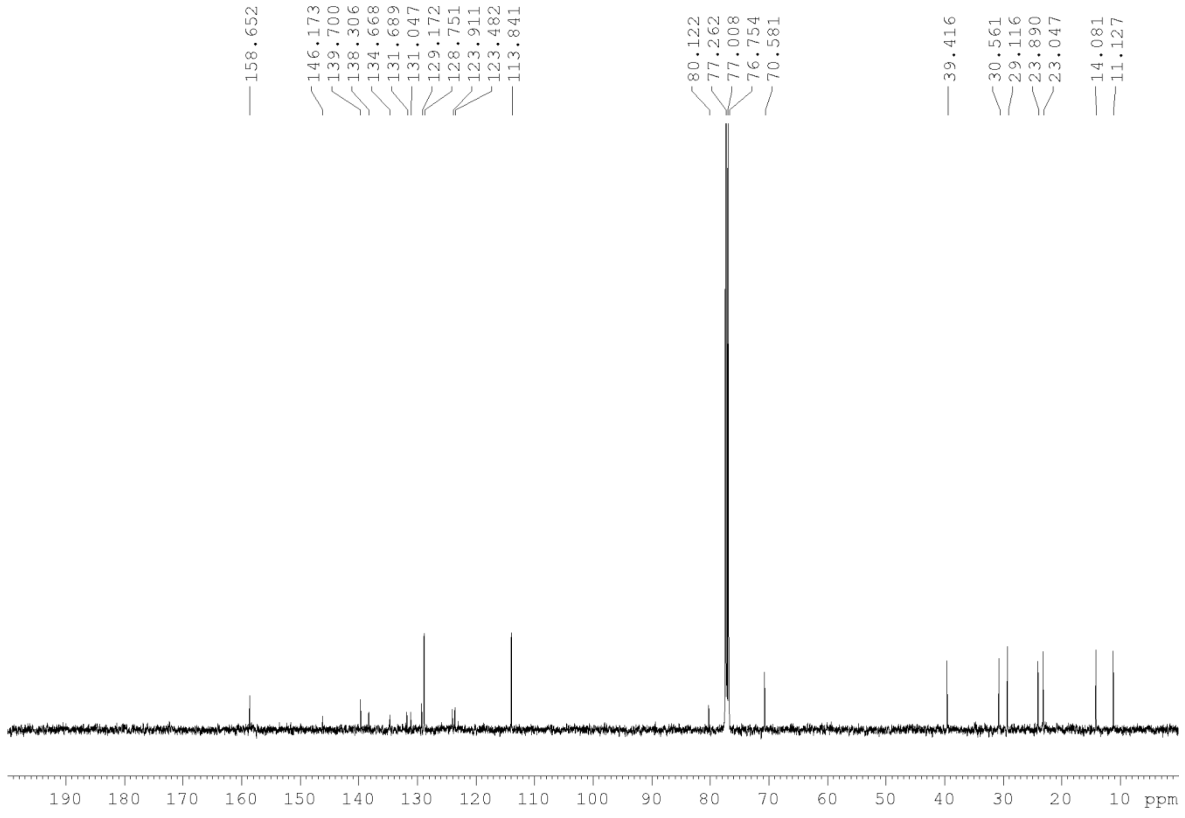

Figure S34. ^{13}C NMR spectrum of compound **8a** in $CDCl_3$

Formula	Mass	Error	mSigma	DblEq	N rule	Electron Configuration
C 74 H 94 O 6 S 4	1,206.5928	0.9079	154.6567	28.00	ok	odd

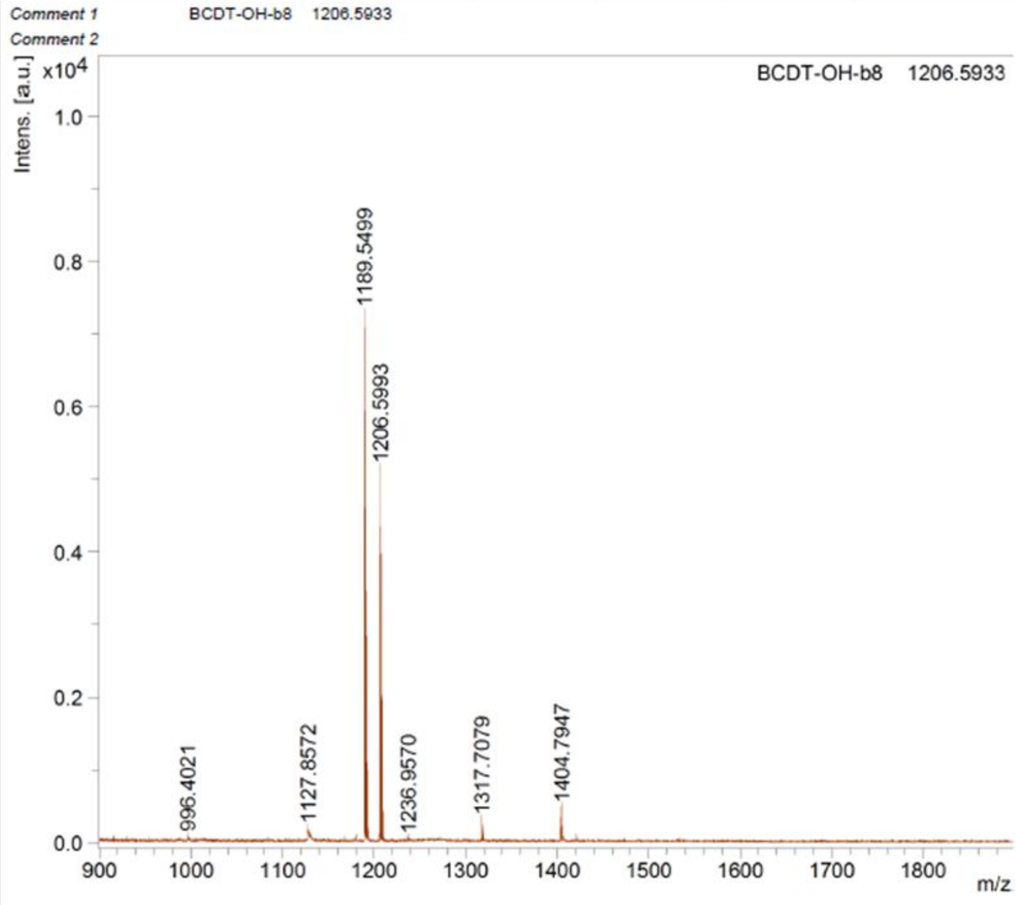

Figure S35. HRMS spectrum of compound **8a**

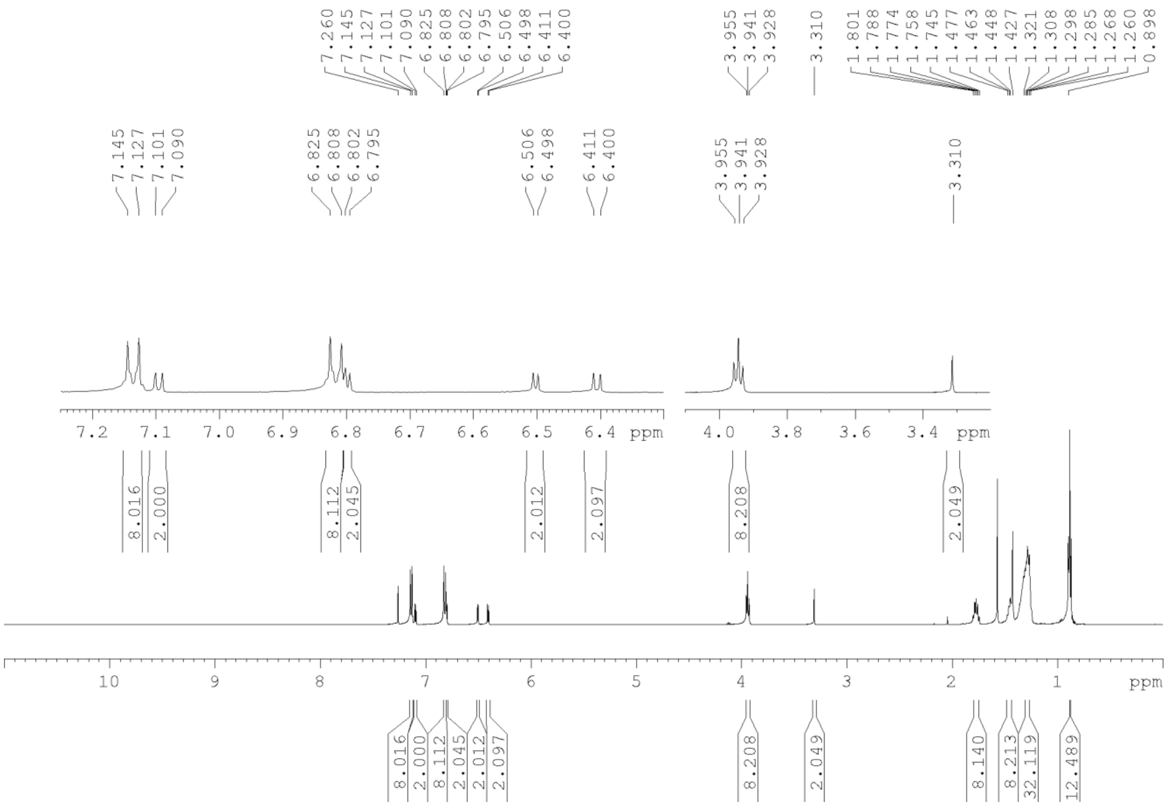

Figure S36. ^{1}H NMR spectrum of compound **8b** in $CDCl_3$

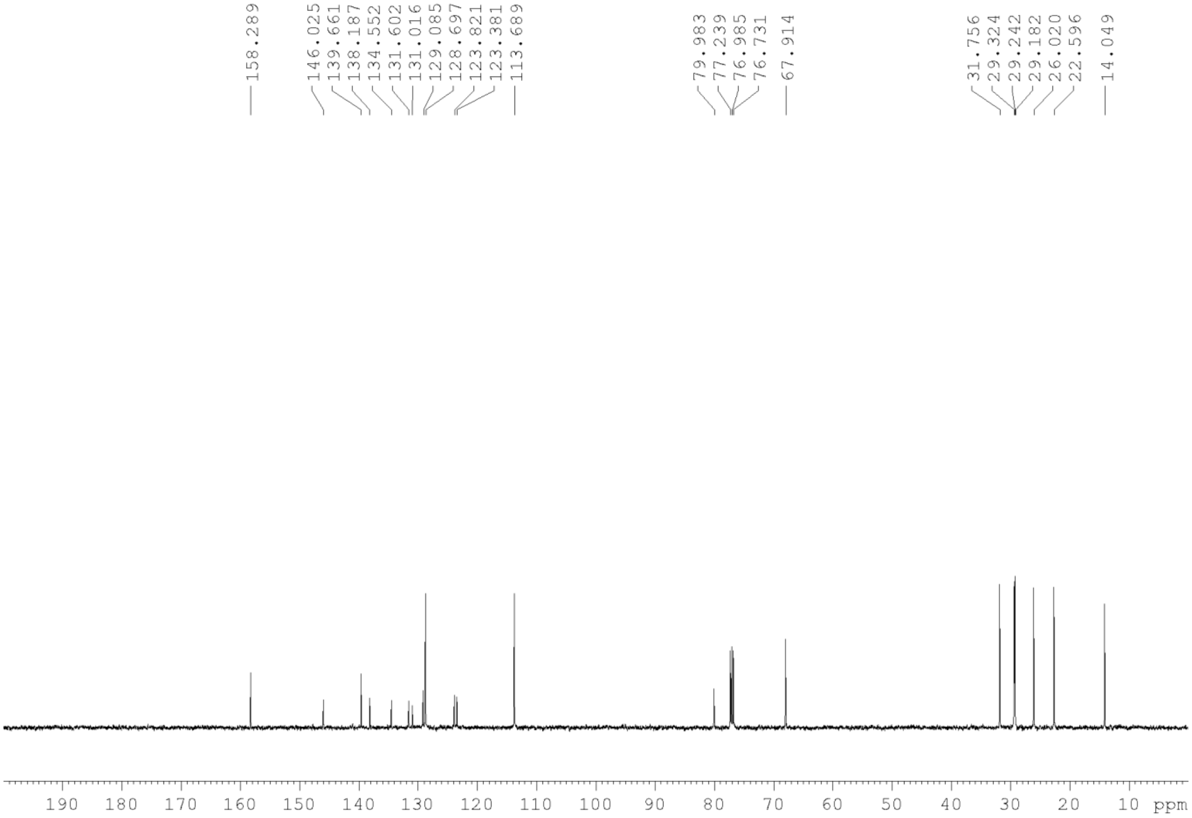

Figure S37. ^{13}C NMR spectrum of compound **8b** in $CDCl_3$

Formula	Mass	Error	mSigma	DblEq	N rule	Electron Configuration
C 74 H 94 O 6 S 4	1,206.5928	0.9079	154.6567	28.00	ok	odd

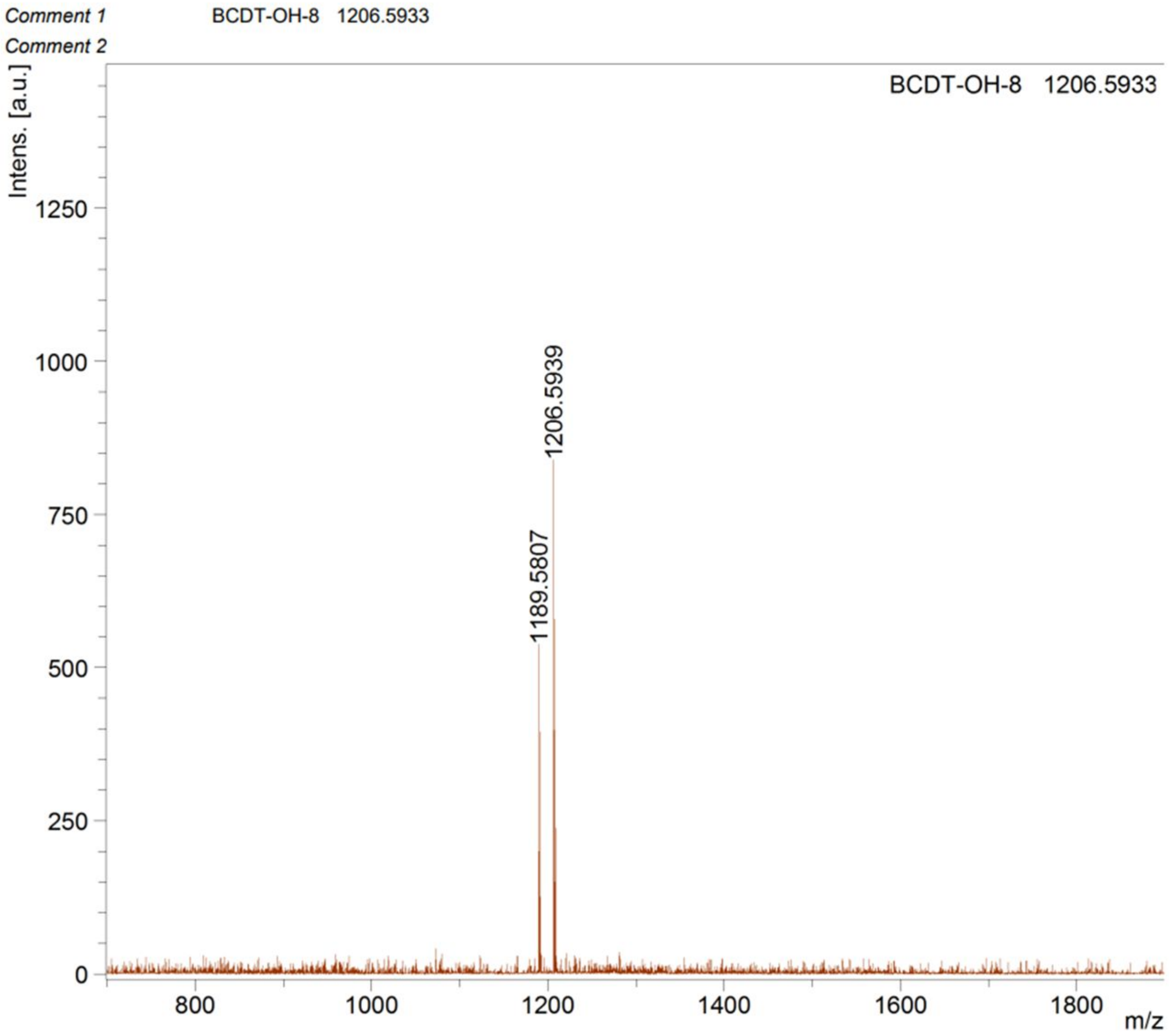

Figure S38. HRMS spectrum of compound **8b**

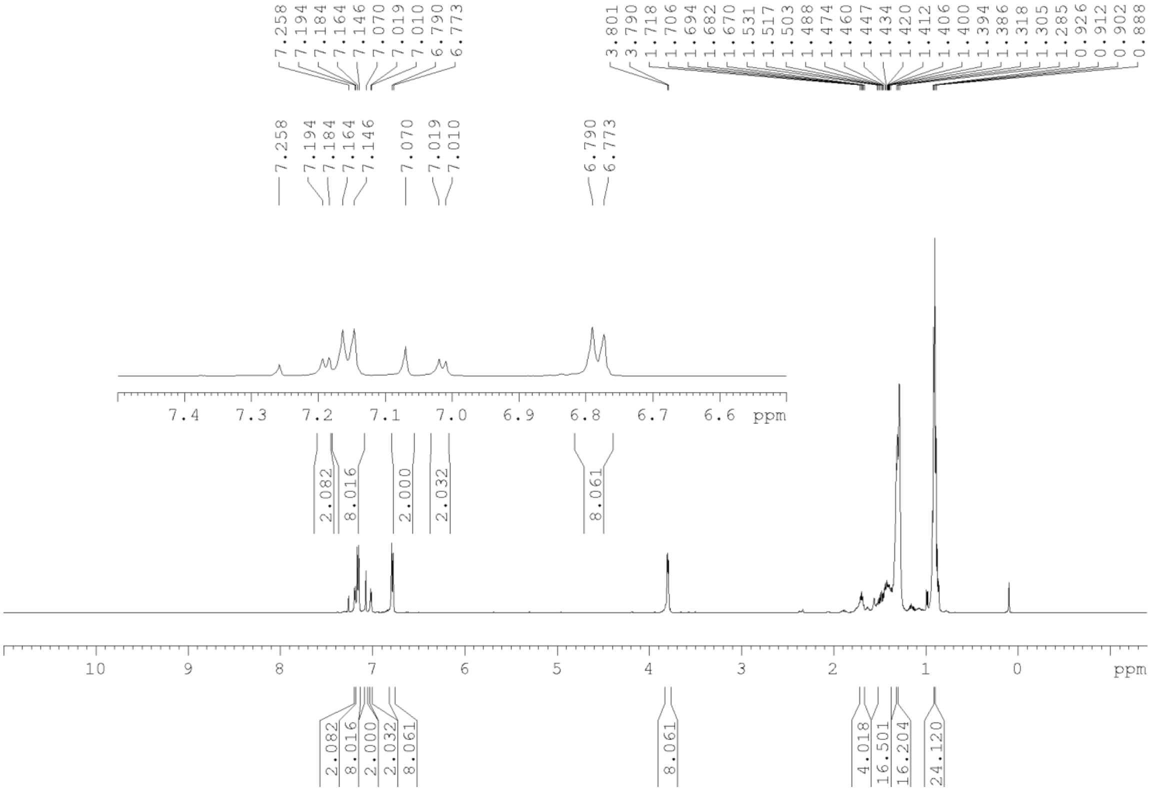

Figure S39. ^{1}H NMR spectrum of compound **9a** in $CDCl_3$

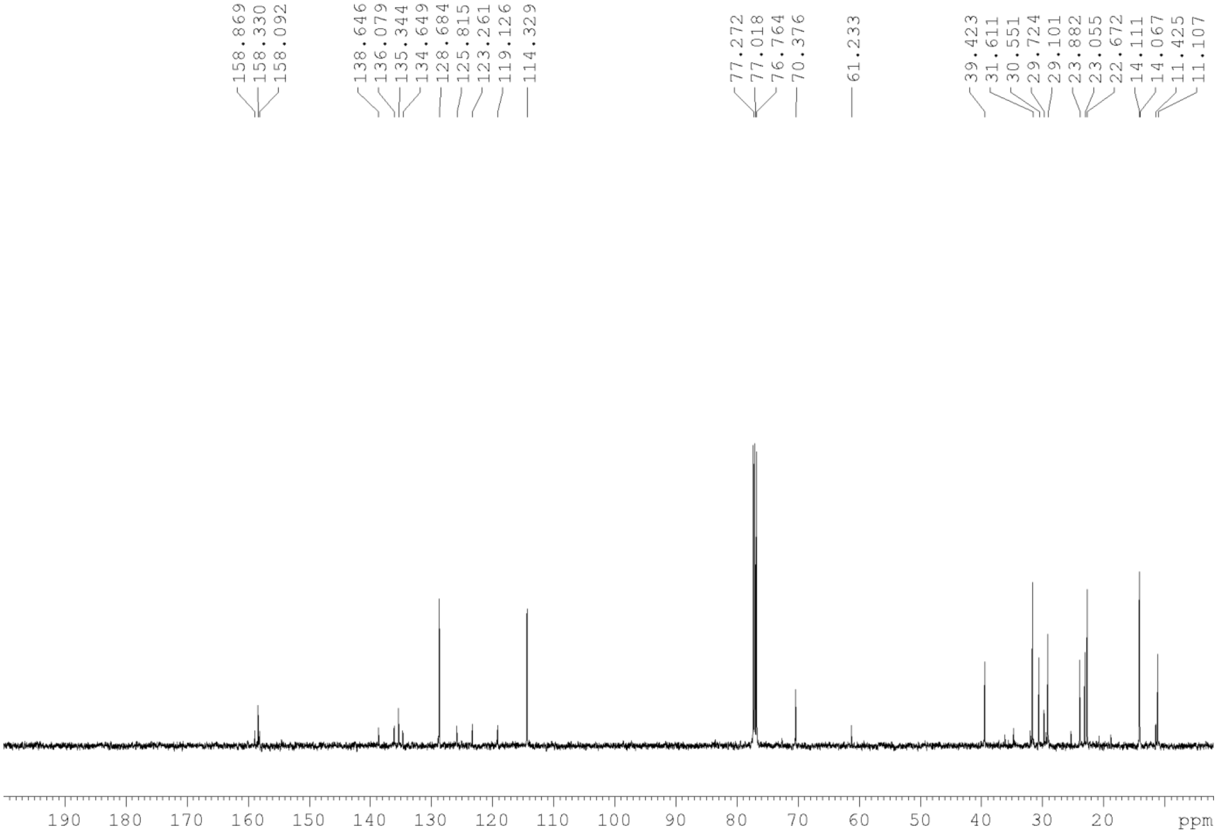

Figure S40. ^{13}C NMR spectrum of compound **9a** in $CDCl_3$

Formula	Mass	Error	mSigma	DblEq	N rule	Electron Configuration
C 74 H 90 O 4 S 4	1,170.5716	3.4483	44.1794	30.00	ok	odd

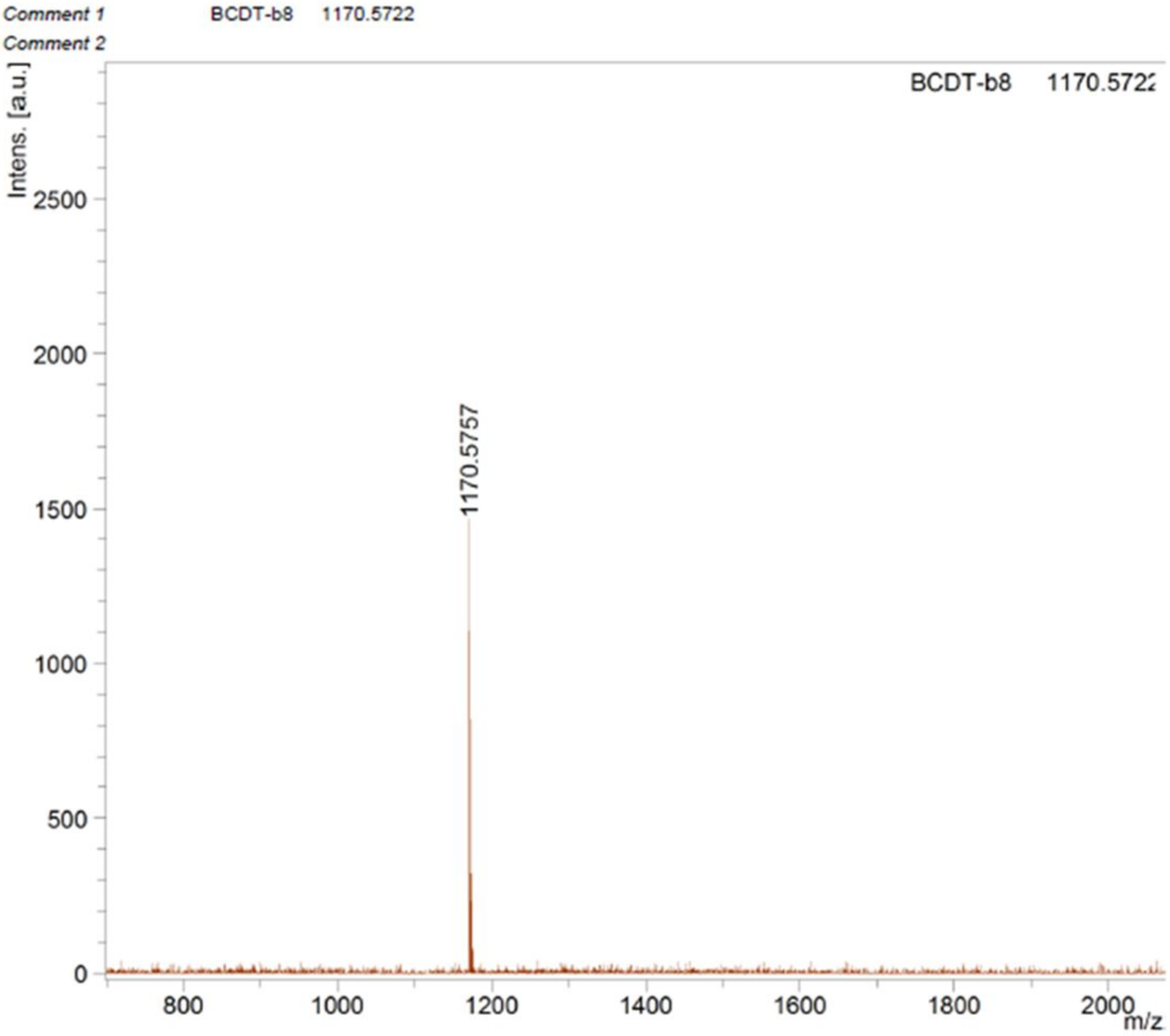

Figure S41. HRMS spectrum of compound **9a**

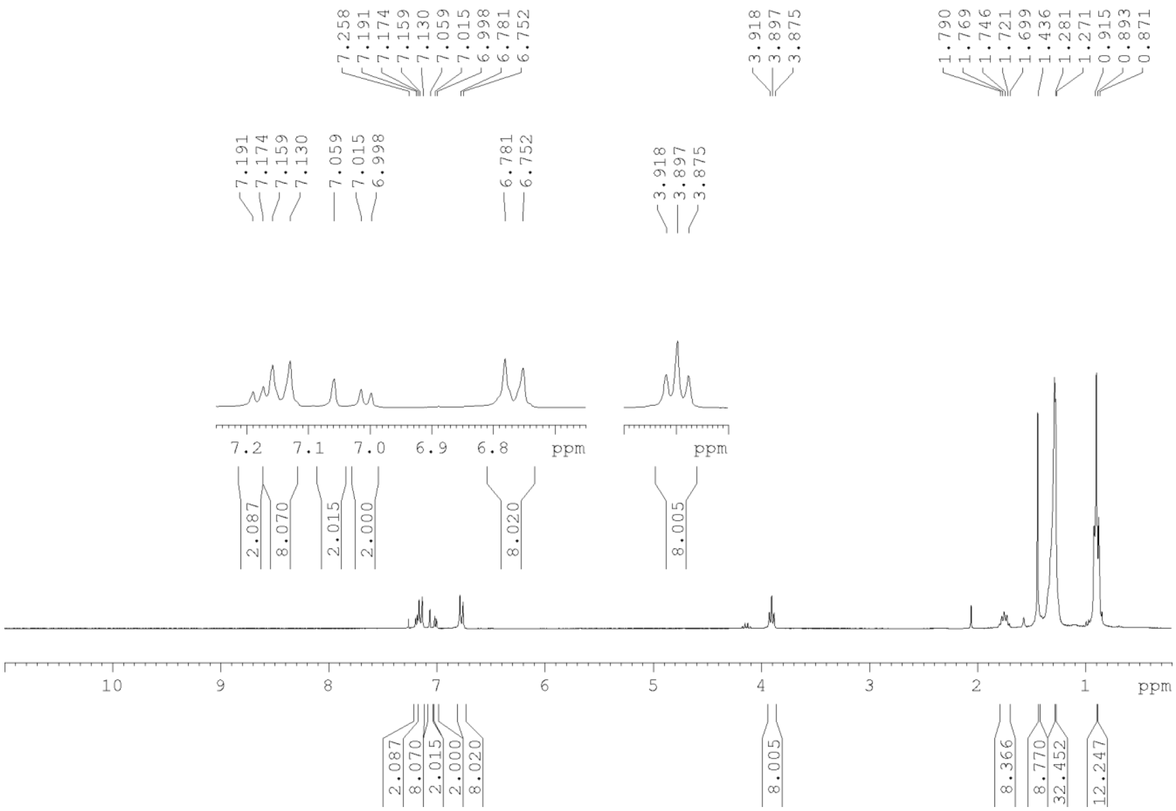

Figure S42. ^{1}H NMR spectrum of compound **9b** in $CDCl_3$

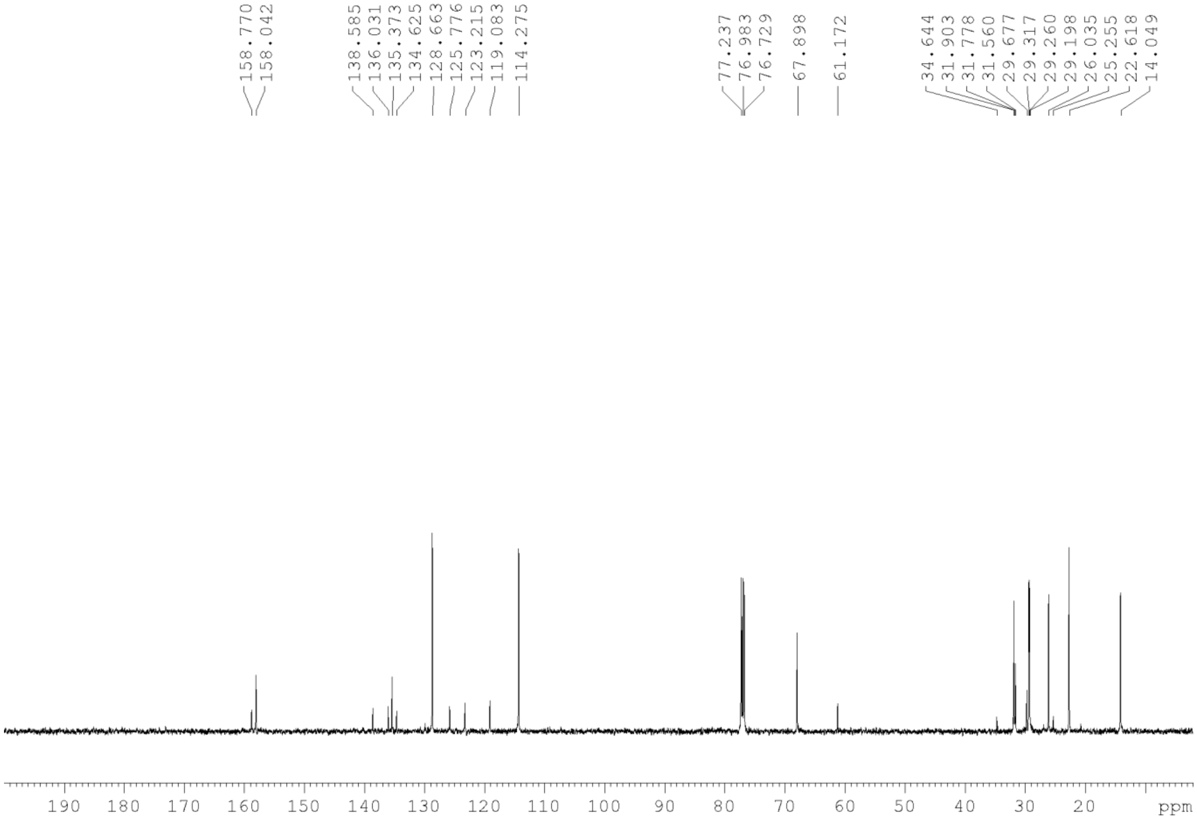

Figure S43. ^{13}C NMR spectrum of compound **9b** in $CDCl_3$

Formula	Mass	Error	mSigma	DblEq	N rule	Electron Configuration
C 74 H 90 O 4 S 4	1,170.5716	3.7635	45.6321	30.00	ok	odd

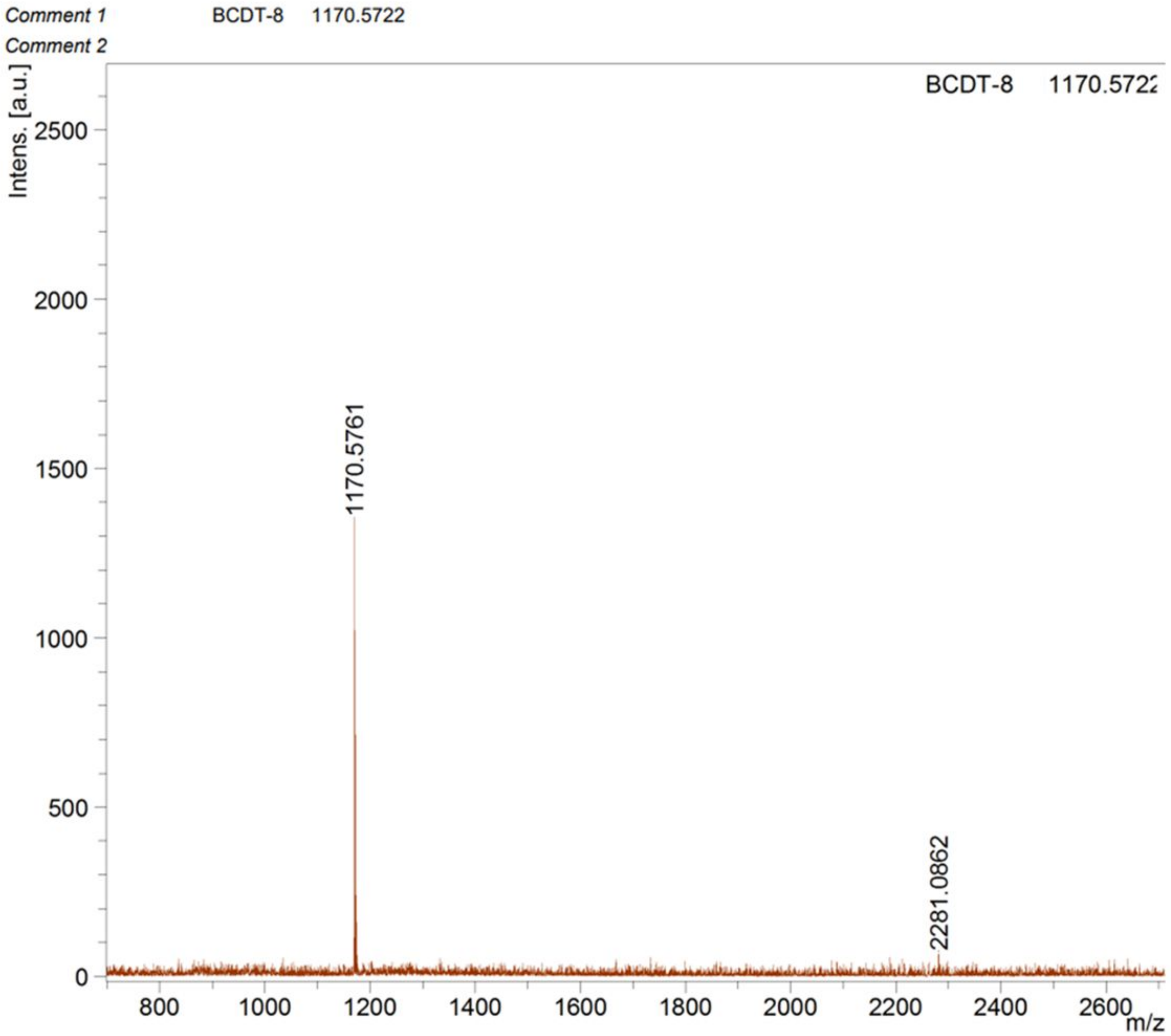

Figure S44. HRMS spectrum of compound **9b**

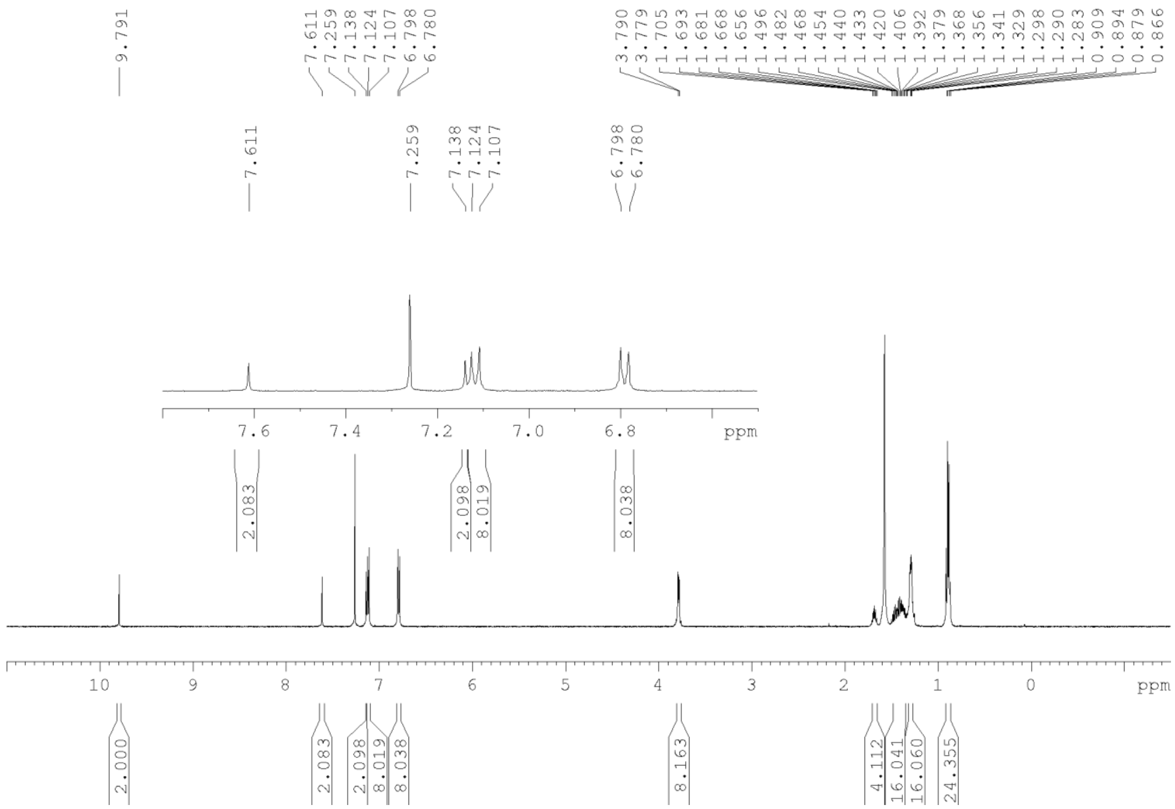

Figure S45. ^{1}H NMR spectrum of compound **10a** in $CDCl_3$

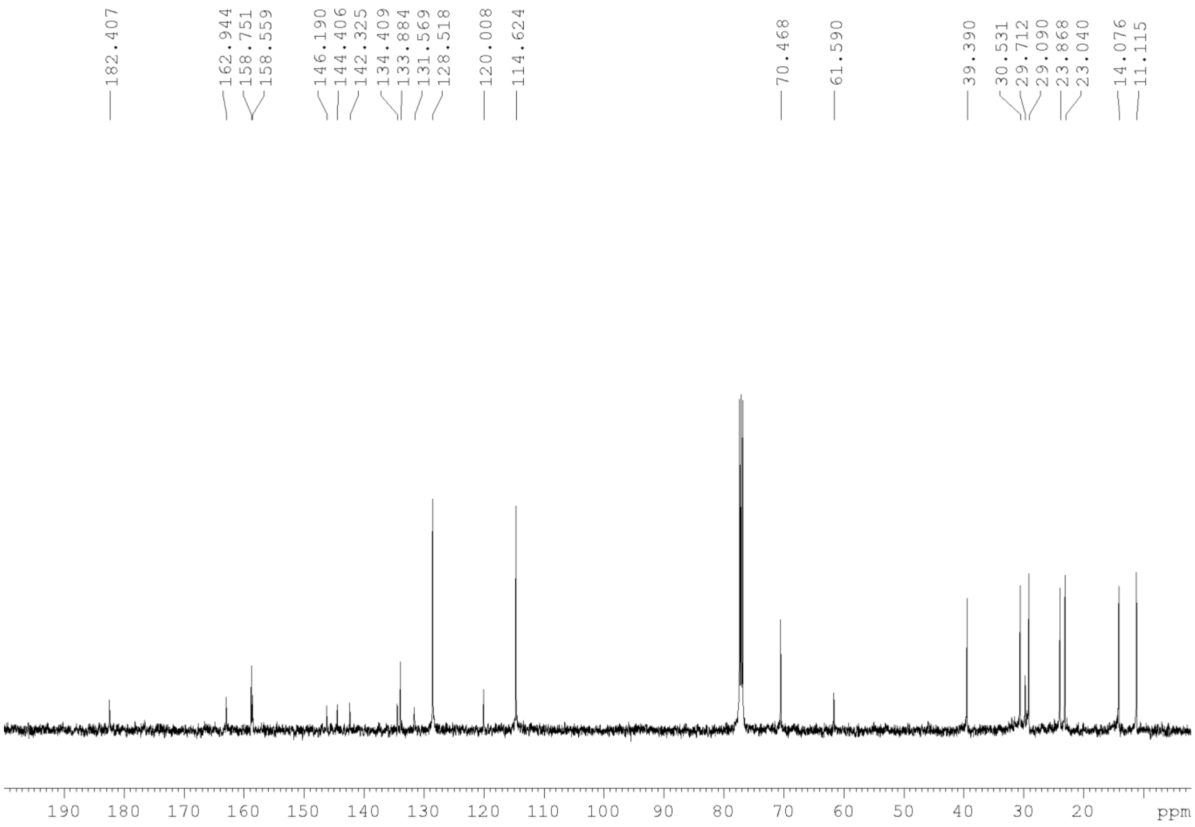

Figure S46. ^{13}C NMR spectrum of compound **10a** in $CDCl_3$

Formula	Mass	Error	mSigma	DblEq	N rule	Electron Configuration
C 76 H 90 O 6 S 4	1,226.5615	7.0310	48.0236	32.00	ok	odd

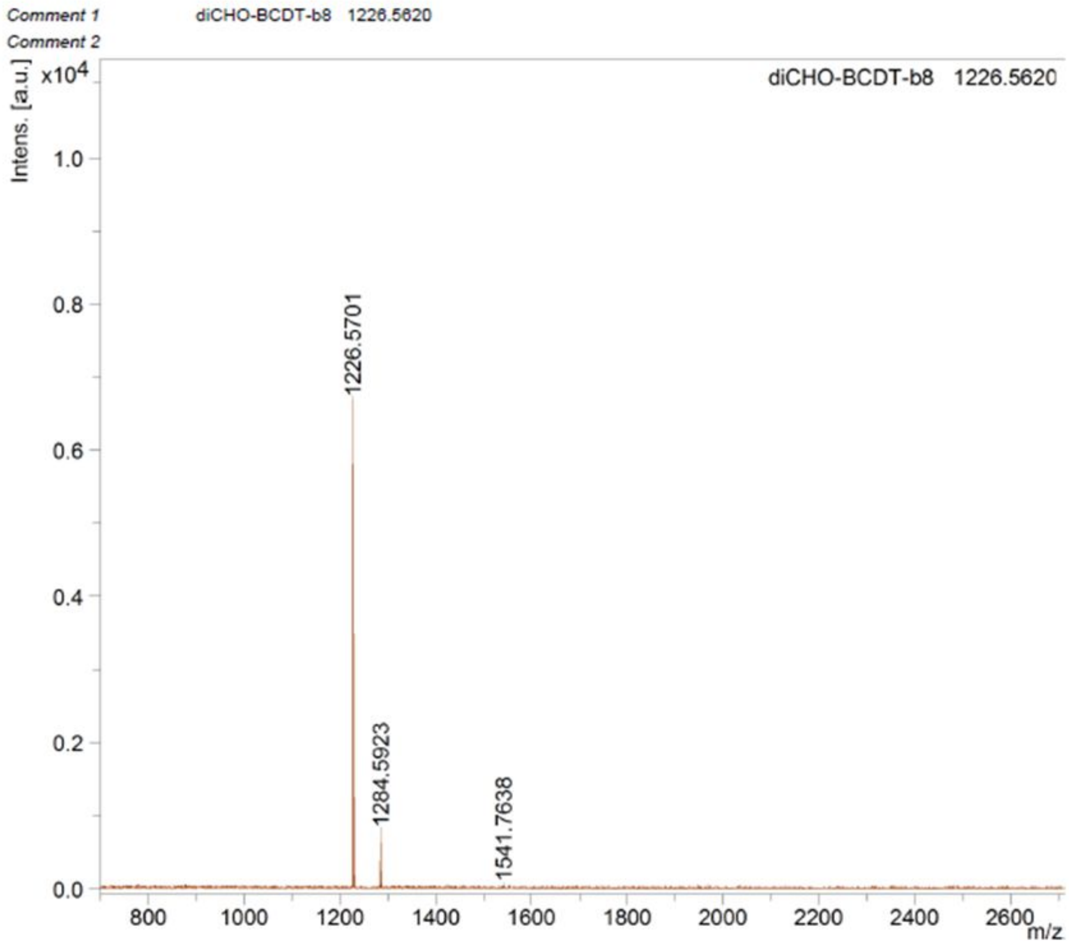

Figure S47. HRMS spectrum of compound **10a**

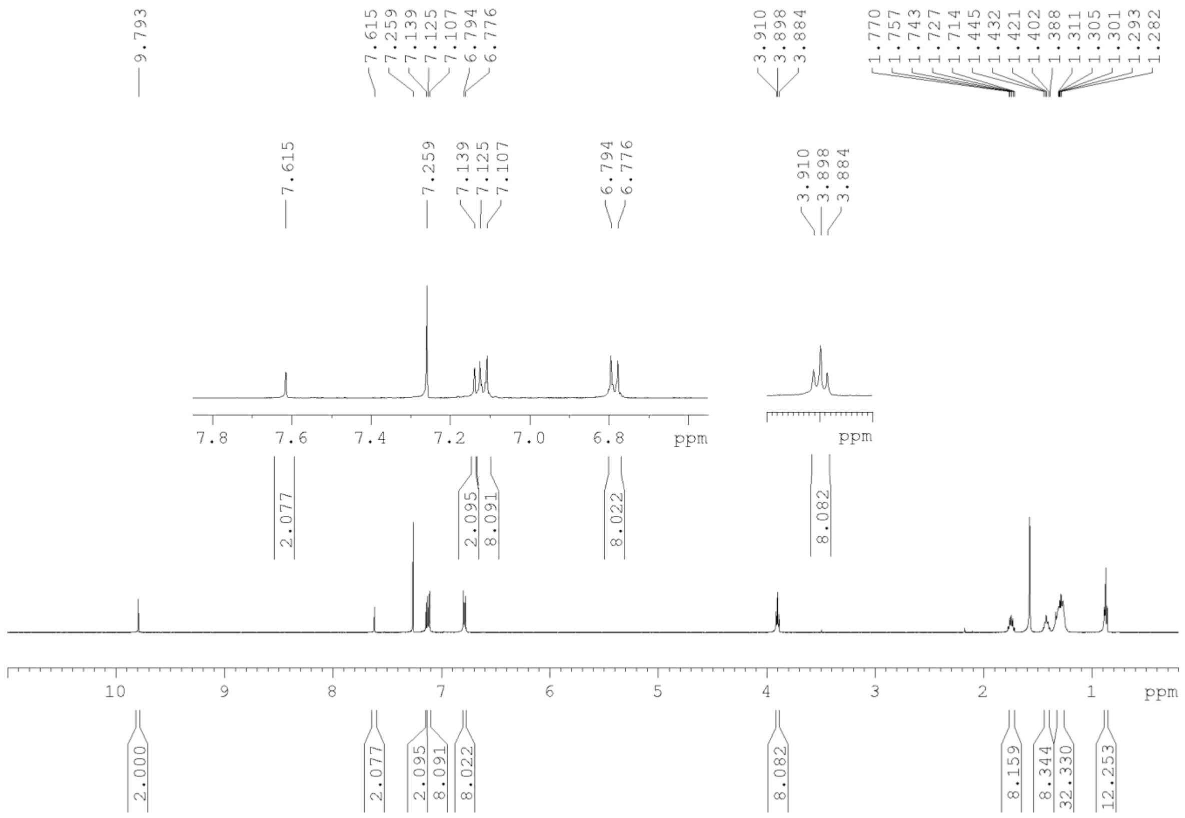

Figure S48. ^{1}H NMR spectrum of compound **10b** in $CDCl_3$

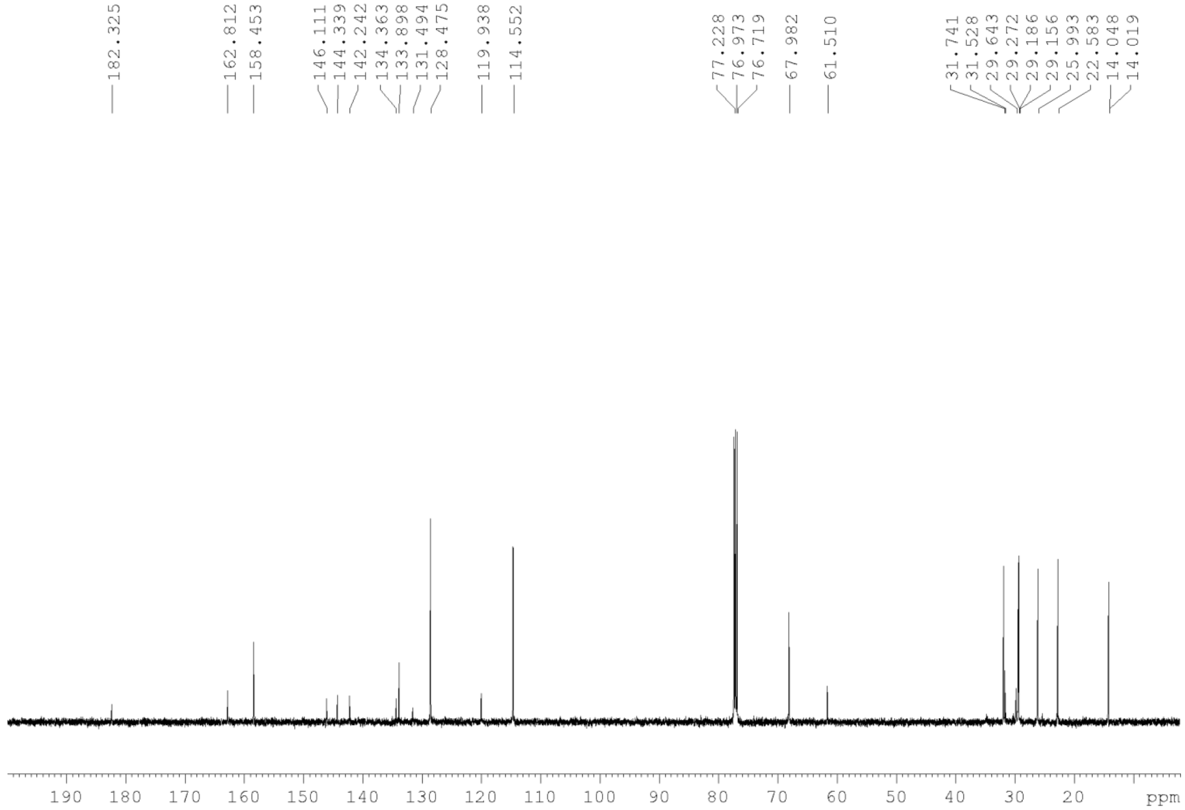

Figure S49. ^{13}C NMR spectrum of compound **10b** in $CDCl_3$

Formula	Mass	Error	mSigma	DblEq	N rule	Electron Configuration
C 76 H 90 O 6 S 4	1,226.5615	6.4207	73.4382	32.00	ok	odd

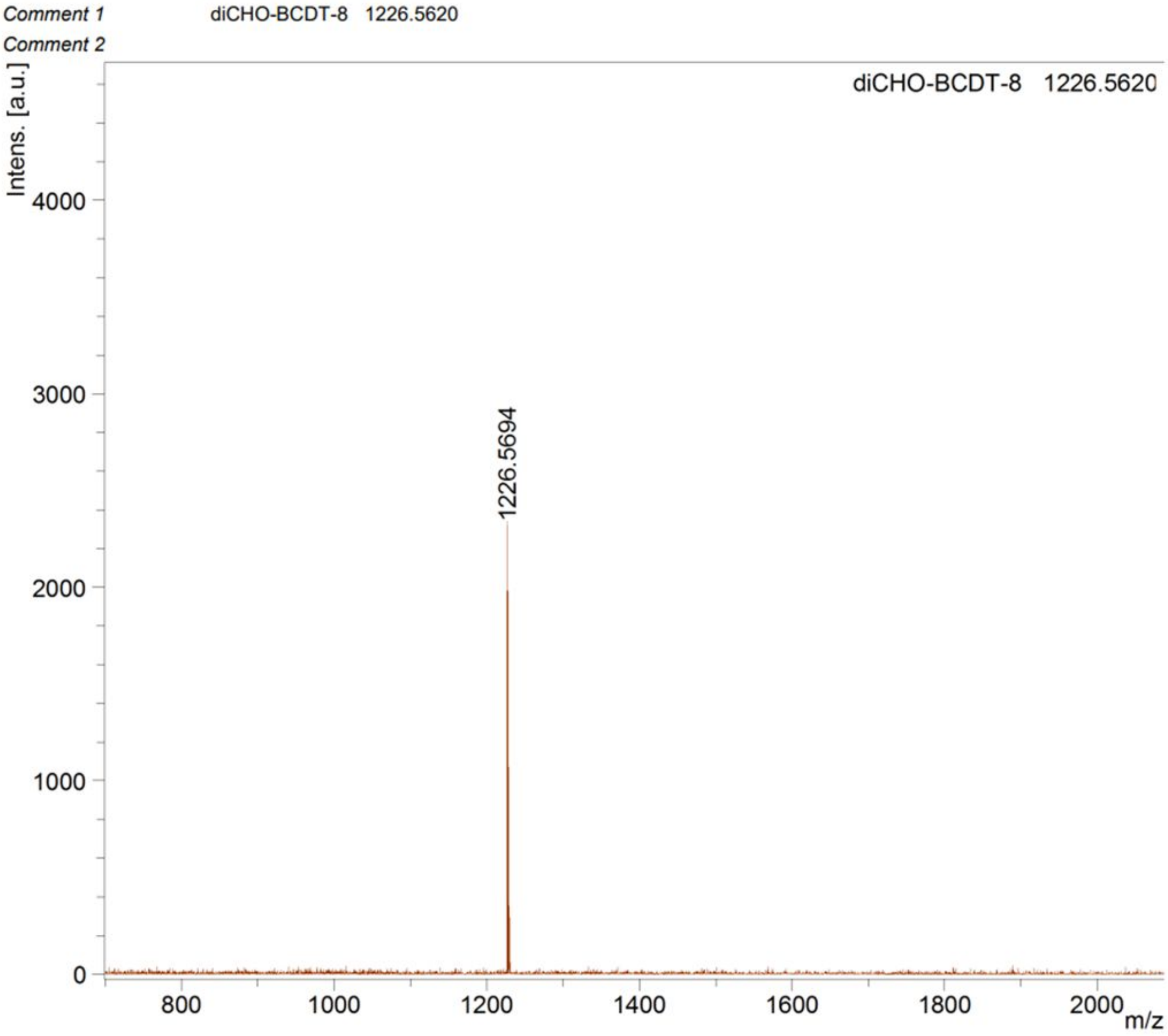

Figure S50. HRMS spectrum of compound **10b**

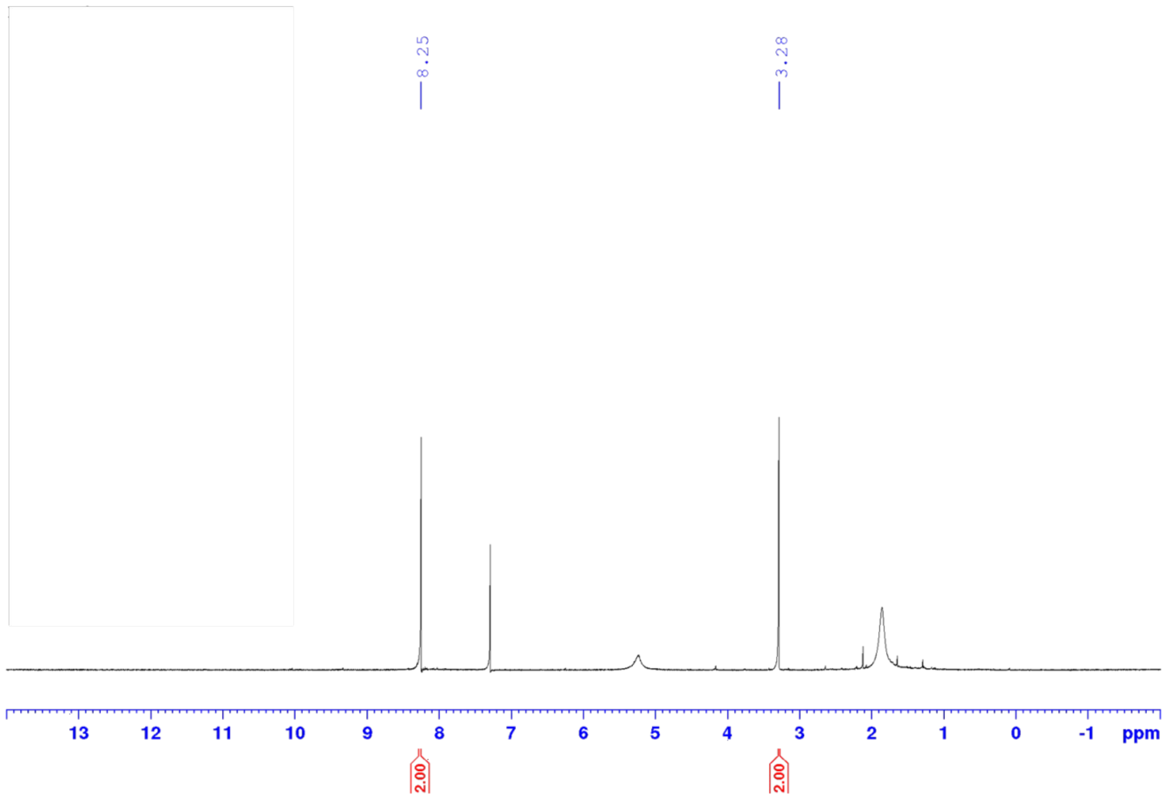

Figure S51. ^{1}H NMR spectrum of compound **15** in $CDCl_3$

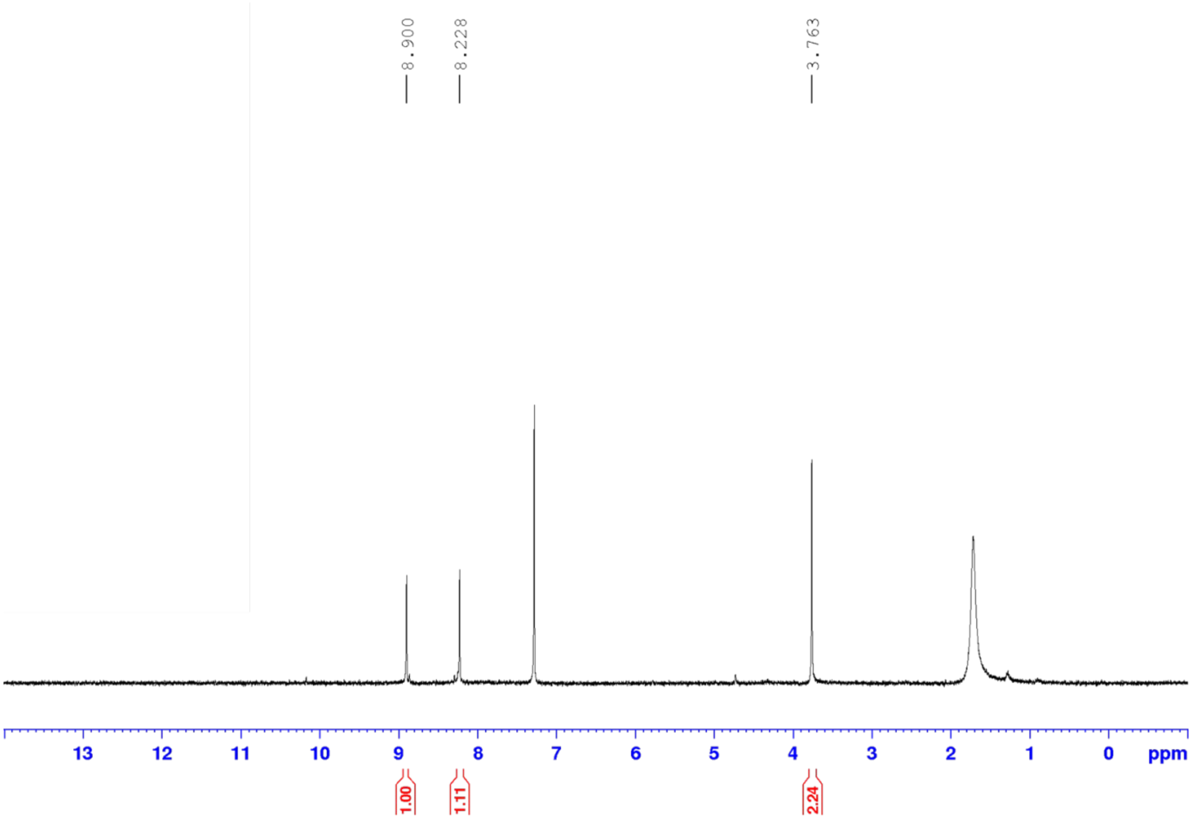

Figure S52. ^{1}H NMR spectrum of compound **13** in $CDCl_3$

References

(1) Du, Z.; Chen, W.; Qiu, M.; Chen, Y.; Wang, N.; Wang, T.; Sun, M.; Yu, D.; Yang, R. Utilizing Alkoxyphenyl Substituents for Side-Chain Engineering of Efficient Benzo[1,2-b:4,5-b´]Dithiophene-Based Small Molecule Organic Solar Cells. *Phys. Chem. Chem. Phys.* **2015**, *17*, 17391-17398.

(2) Hucke, A.; Cava, M. P. Synthesis of Mixed Thiophene/Furan Oligomers by Stille Coupling. *J. Org. Chem.* **1998**, *63*, 7413-7417.

(3) Lee, T.-H.; Wu, K.-Y.; Lin, T.-Y.; Wu, J.-S.; Wang, C.-L.; Hsu, C.-S. Role of the Comonomeric Units in Reaching Linear Backbone, High Solid-State Order and Charge Mobilities in Heptacyclic Arene-Based Alternating Copolymers. *Macromolecules* **2013**, *46*, 7687-7695.

(4) Bai, H.; Wang, Y.; Cheng, P.; Wang, J.; Wu, Y.; Hou, J.; Zhan, X. An Electron Acceptor Based on Indacenodithiophene and 1,1-dicyanomethylene-3-indanone for Fullerene-Free Organic Solar Cells. *J. Mater. Chem. A* **2015**, *3*, 1910-1914.

(5) Cui, Y.; Yang, C.; Yao, H.; Zhu, J.; Wang, Y.; Jia, G.; Gao, F.; Hou, J. Efficient Semitransparent Organic Solar Cells with Tunable Color enabled by an Ultralow-Bandgap Nonfullerene Acceptor. *Adv. Mater.* **2017**, *29*, 1703080.

(6) Huang, X.; Hu, M.; Zhao, X.; Li, C.; Yuan, Z.; Liu, X.; Cai, C.; Zhang, Y.; Hu, Y.; Chen, Y. Subphthalocyanine Triimides: Solution Processable Bowl-Shaped Acceptors for Bulk Heterojunction Solar Cells. *Org. Lett.* **2019**, *21*, 3382-3386.

(7) Singh, M.; Chiang, C.-H.; Boopathi, K. M.; Hanmandlu, C.; Li, G.; Wu, C.-G.; Lin, H.-C.; Chu, C.-W. A Novel Ball Milling Technique for Room Temperature Processing of TiO_2 Nanoparticles Employed as the Electron Transport Layer in Perovskite Solar Cells and Modules.

J. Mater. Chem. A **2018**, *6*, 7114-7122.

(8) Chiang, C.-H.; Nazeeruddin, M. K.; Grätzel, M.; Wu, C.-G. The Synergistic Effect of H_2O and DMF Towards Stable and 20% Efficiency Inverted Perovskite Solar Cells. *Energy Environ. Sci.* **2017**, *10*, 808-817.
